# Atg23 prevents aberrant fusion of Atg9 vesicles during delivery to autophagosome formation sites

**DOI:** 10.1038/s44318-026-00867-0

**Published:** 2026-07-20

**Authors:** Takumi Kimura, Takayuki Shima, Tetsuya Kotani, Hitoshi Nakatogawa

**Affiliations:** 1 Cell Biology Center, Institute of Integrated Research, Institute of Science Tokyo, Yokohama, Japan; 2 School of Life Science and Technology, Institute of Science Tokyo, Yokohama, Japan; 3 Department of Biochemistry, Nara Medical University, Kashihara, Japan; 4 Present Address: Department of Biochemistry, Nara Medical University, Kashihara, Japan

**Keywords:** Autophagy & Cell Death

## Abstract

Autophagosome biogenesis depends on the accurate delivery of membrane lipids to the pre-autophagosomal structure. Golgi/endosome-derived Atg9 vesicles provide the membrane seed for this process, but how they are trafficked through the cytoplasm while avoiding inappropriate fusion remains unclear. Here we show that in *Saccharomyces cerevisiae*, the soluble Atg9-interacting protein Atg23 remains associated with Atg9 vesicles after their biogenesis. This association shields Atg9 vesicles from aberrant SNARE-dependent fusion as they diffuse through the cytoplasm en route to the autophagosome formation site. We further show that upon vesicle arrival at the site, Atg9 phosphorylation by the autophagy initiation kinase Atg1 releases Atg23, facilitating recruitment of the downstream factor Atg2, a lipid transfer protein for membrane expansion. Together, these findings reveal an Atg1-dependent phosphorylation switch that regulates Atg9 vesicle dynamics during autophagosome biogenesis.

## Synopsis

Atg9 vesicles provide the nucleating membrane for autophagosome biogenesis. This study identifies a mechanism that prevents their premature fusion during delivery, and explains how this inhibition is relieved upon arrival at the autophagosome formation site.

- Atg23, which functions in Atg9 vesicle biogenesis, remains associated with Atg9 after vesicle formation.
- Atg23 prevents SNARE-dependent premature fusion of Atg9 vesicles during their delivery to the autophagosome formation site.
- Atg23 dissociates from Atg9 at the autophagosome formation site upon Atg9 phosphorylation by Atg1.
- Timely dissociation of Atg23 promotes Atg2 recruitment and autophagosome formation.

## Abstract (web summary)

Atg23 shields Atg9 vesicles until Atg1-dependent release at autophagosome formation sites.

## Graphical abstract

**Figure:**
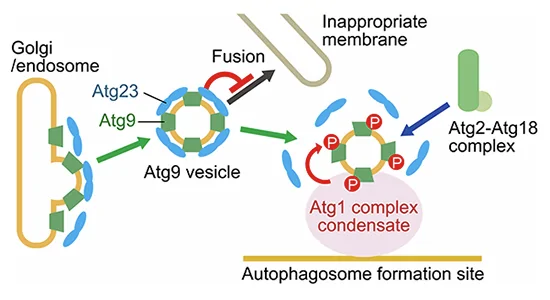

**Figure:**
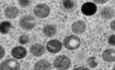

## Introduction

Macroautophagy (hereafter referred to as autophagy) is a major pathway for the degradation of cellular materials in lysosomes or vacuoles in eukaryotes and plays diverse physiological roles, including adaptation to starvation, regulation of cellular metabolism, and quantity and quality control of proteins and organelles, thereby contributing to the prevention of aging and various diseases (Nakatogawa et al, 2009; Ohsumi, 2014; Yang and Klionsky, 2010; Dikic and Elazar, 2018; Levine and Kroemer, 2019; Yamamoto et al, 2023). When autophagy is induced, a membrane cisterna called the isolation membrane (or phagophore) forms, expands into a spherical shape, and finally closes to form a double-membrane vesicle called the autophagosome. During this process, cellular components destined for degradation are enclosed within the autophagosome selectively or non-selectively. Subsequently, the autophagosome fuses with the lysosome or the vacuole, resulting in the degradation of the enclosed components. Autophagosome formation requires a unique set of proteins called core autophagy-related (Atg) proteins (Nakatogawa, 2020). These proteins constitute six functional units, as defined in *Saccharomyces cerevisiae*: (i) the Atg1 kinase and its associated factors (Atg13, Atg17, Atg31, and Atg29) (hereafter referred to as the Atg1 complex), (ii) Atg9 vesicles, (iii) phosphatidylinositol 3-kinase complex I (PI3KCI), (iv) the Atg12 conjugation system, (v) the Atg8 lipidation system, and (vi) the Atg2-Atg18 complex. Upon autophagy induction, these units assemble on the vacuolar membrane to form the pre-autophagosomal structure (PAS), in which phase-separated condensates of the Atg1 complex serve as a scaffold (Suzuki et al, 2007; Fujioka et al, 2020). Atg9 is an integral membrane protein with four transmembrane helices and forms a trimer (Lang et al, 2000; Noda et al, 2000; Matoba et al, 2020; Maeda et al, 2020). The majority of Atg9 exists in the form of Atg9 vesicles, which are single-membrane vesicles with a diameter of 30–60 nm derived from the Golgi or endosomes (Yamamoto et al, 2012; Mari et al, 2010; Shirahama-Noda et al, 2013). Atg9 vesicles diffuse in the cytoplasm and eventually localize to the PAS through the interaction of Atg9 with Atg13, a component of the Atg1 complex (Suzuki et al, 2015; Yamamoto et al, 2012). Previous studies have suggested that several Atg9 vesicles are then converted to an autophagosome precursor at the PAS (Yamamoto et al, 2012; Sawa-Makarska et al, 2020), which expands into the isolation membrane given lipid supply from the endoplasmic reticulum (ER) mediated by the Atg2-Atg18 complex (Osawa et al, 2019; Valverde et al, 2019; Kotani et al, 2018; Maeda et al, 2019). During this process, Atg9 scrambles lipids within the precursor and isolation membranes to facilitate membrane expansion (Matoba et al, 2020; Maeda et al, 2020).

Atg23 is a soluble protein that interacts with Atg9 in *S. cerevisiae* (Meiling-Wesse et al, 2004; Tucker et al, 2003). In cells lacking Atg23, the number of Atg9 vesicles is lower than in wild-type cells, accompanied by Atg9 accumulation in the Golgi and endosomes and a decline in autophagic activity (Yamamoto et al, 2012; Backues et al, 2015). In addition, Atg23 was reported to mediate the targeting of Atg9 vesicles to the PAS through its interaction with the Rab GTPase Ypt1, which is recruited to the PAS via its association with the Atg1 complex (Yao et al, 2023). It was also reported that the reduction of autophagic activity caused by the loss of Atg23 is suppressed by overexpression of Atg9 (Backues et al, 2015). Based on these observations, Atg23 has been considered to be involved in the formation and trafficking of Atg9 vesicles, but the molecular details remain unknown.

In this study, we uncover a novel function of Atg23: it remains associated with Atg9 vesicles during their trafficking toward the PAS to prevent aberrant fusion of the vesicles. Upon reaching the PAS and subsequent phosphorylation of Atg9 by Atg1, Atg23 dissociates from Atg9 vesicles, thereby allowing the recruitment of downstream factors and facilitating the progression of autophagosome formation.

## Results

### Atg23 dissociates from Atg9 vesicles at the PAS in an Atg1 kinase-dependent manner

**Figure 1:**
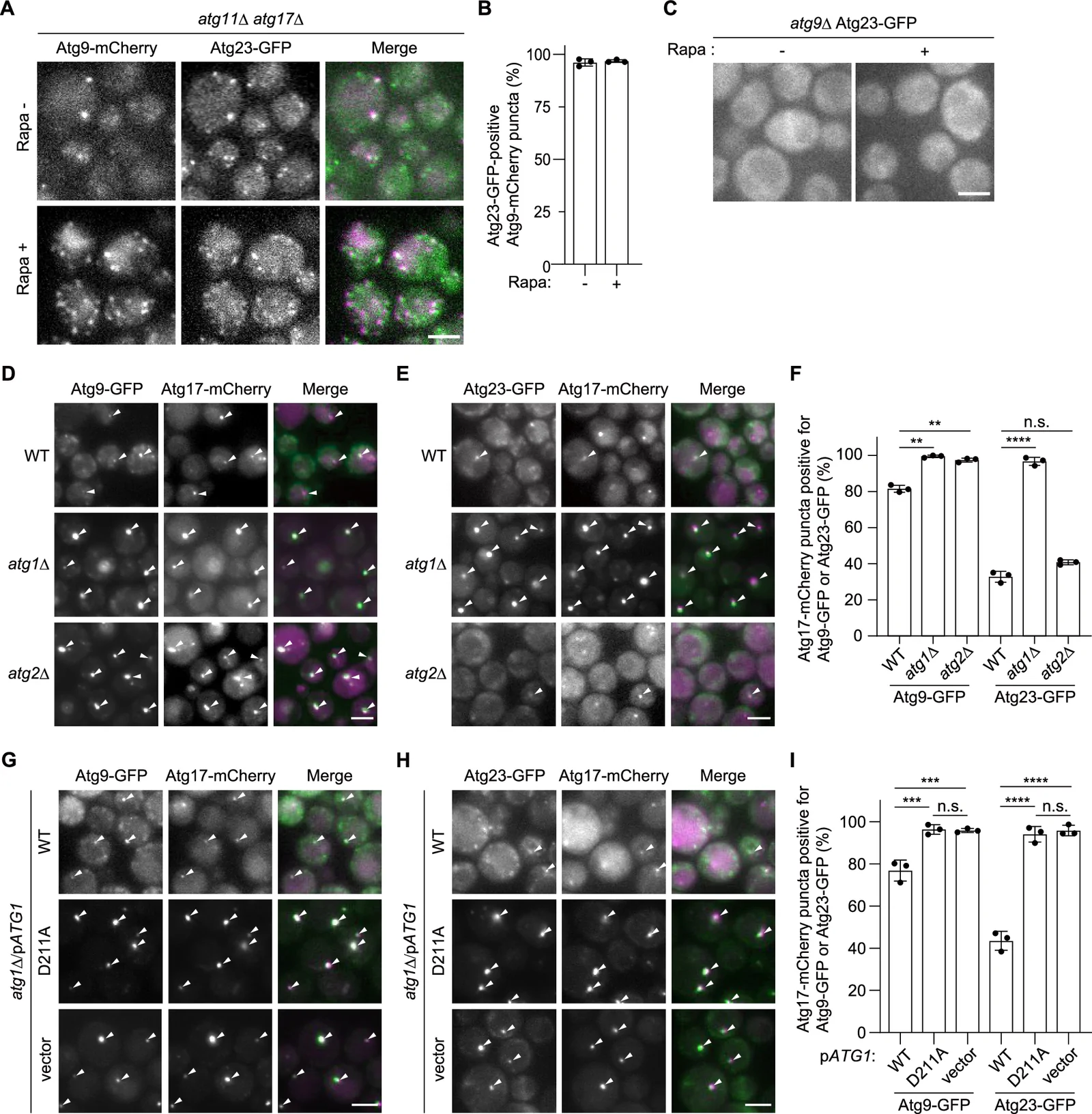
Atg23 is released from Atg9 vesicles at the PAS in an Atg1-dependent manner. (**A**) *atg11*Δ *atg17*Δ cells expressing Atg9-mCherry and Atg23-GFP were treated with rapamycin (Rapa) or ethanol as a vehicle control for 2 h and analyzed by fluorescence microscopy. Scale bar, 5 μm. (**B**) Quantification of the percentage of Atg9-mCherry puncta positive for Atg23-GFP in the experiments shown in (**A**). Bars represent means ± SD (*n* = 3 independent biological replicates). (**C**) *atg9*Δ cells expressing Atg23-GFP were treated with rapamycin or ethanol as a vehicle control for 2 h and analyzed by fluorescence microscopy. Scale bar, 5 μm. (**D**, **E**) Cells expressing Atg17-mCherry and either Atg9-GFP (**D**) or Atg23-GFP (**E**) were treated with rapamycin for 2 h and analyzed by fluorescence microscopy. Scale bars, 5  μm. White arrowheads, Atg17-mCherry puncta positive for Atg9-GFP or Atg23-GFP. (**F**) Quantification of the percentage of Atg17-mCherry puncta positive for Atg9-GFP or Atg23-GFP in the experiments shown in (**D**, **E**). Bars represent means ± SD (*n* = 3 independent biological replicates). **P < 0.01, ****P < 0.0001, n.s., not significant (Tukey’s multiple comparisons test): Atg9 WT vs Atg9 *atg1*Δ *P*  =  2.4 × 10^−3^; Atg9 WT vs Atg9 *atg2*Δ *P*  =  9.3 × 10^−3^; Atg23 WT vs Atg23 *atg1*Δ *P* = 6.1 × 10^−6^; Atg23 WT vs Atg23 *atg2*Δ *P*  =  1.1×10^−1^. (**G**, **H**) Cells expressing Atg17-mCherry and either Atg9-GFP (**G**) or Atg23-GFP (**H**) were treated with rapamycin for 2 h and analyzed by fluorescence microscopy. Scale bars, 5 μm. White arrowheads, Atg17-mCherry puncta positive for Atg9-GFP or Atg23-GFP. (**I**) Quantification of the percentage of Atg17-mCherry puncta positive for Atg9-GFP or Atg23-GFP in the experiments shown in (**G**, **H**). Bars represent means ± SD (*n* = 3 independent biological replicates). ***P < 0.001, ****P < 0.0001, n.s., not significant (Tukey’s multiple comparisons test): Atg9 Atg1^WT^ vs Atg9 Atg1^D211A^ *P*  =  1.7 × 10^−4^; Atg9 Atg1^WT^ vs Atg9 vector *P*  =  2.2 × 10^−4^; Atg9 Atg1^D211A^ vs Atg9 vector *P*  > 0.99; Atg23 Atg1^WT^ vs Atg23 Atg1^D211A^ *P* = 2.6 × 10^−9^; Atg23 Atg1^WT^ vs Atg23 vector *P* = 4.2 × 10^−9^; Atg23 Atg1^D211A^ vs Atg23 vector *P* = 9.8 × 10^−1^. Source data are available online for this figure.

Previous studies suggested that Atg23 associates with Atg9 vesicles (Legakis et al, 2007; Backues et al, 2015; Tucker et al, 2003; Reggiori et al, 2004; Yao et al, 2023). In this study, we began by verifying this model through direct observation of Atg23 localization to Atg9 vesicles using fluorescence microscopy. Cells lacking Atg11 and Atg17 (*atg11*Δ *atg17*Δ cells) do not form Atg1 complex condensates that serve as a PAS scaffold (Cheong et al, 2008; Kawamata et al, 2008), and therefore, most Atg9 exists in the form of Atg9 vesicles in these cells (Yamamoto et al, 2012). In fluorescence microscopy of *atg11*Δ *atg17*Δ cells expressing Atg9 fused with a fluorescent protein, Atg9 vesicles are observed as Atg9-positive puncta diffusing throughout the cytoplasm (Yamamoto et al, 2012). When *atg11*Δ *atg17*Δ cells expressing Atg9-mCherry and Atg23-GFP were observed under a fluorescence microscope, most of Atg9-mCherry puncta were positive for Atg23-GFP, with or without rapamycin treatment that induces autophagy (Fig. 1A,B). In addition, Atg23-GFP did not form puncta and was completely diffused in the cytoplasm in the absence of Atg9, consistent with a previous study (Tucker et al, 2003) (Fig. 1C). These data allowed us to conclude that Atg23 remains associated with Atg9 vesicles after their biogenesis.

**Figure EV1:**
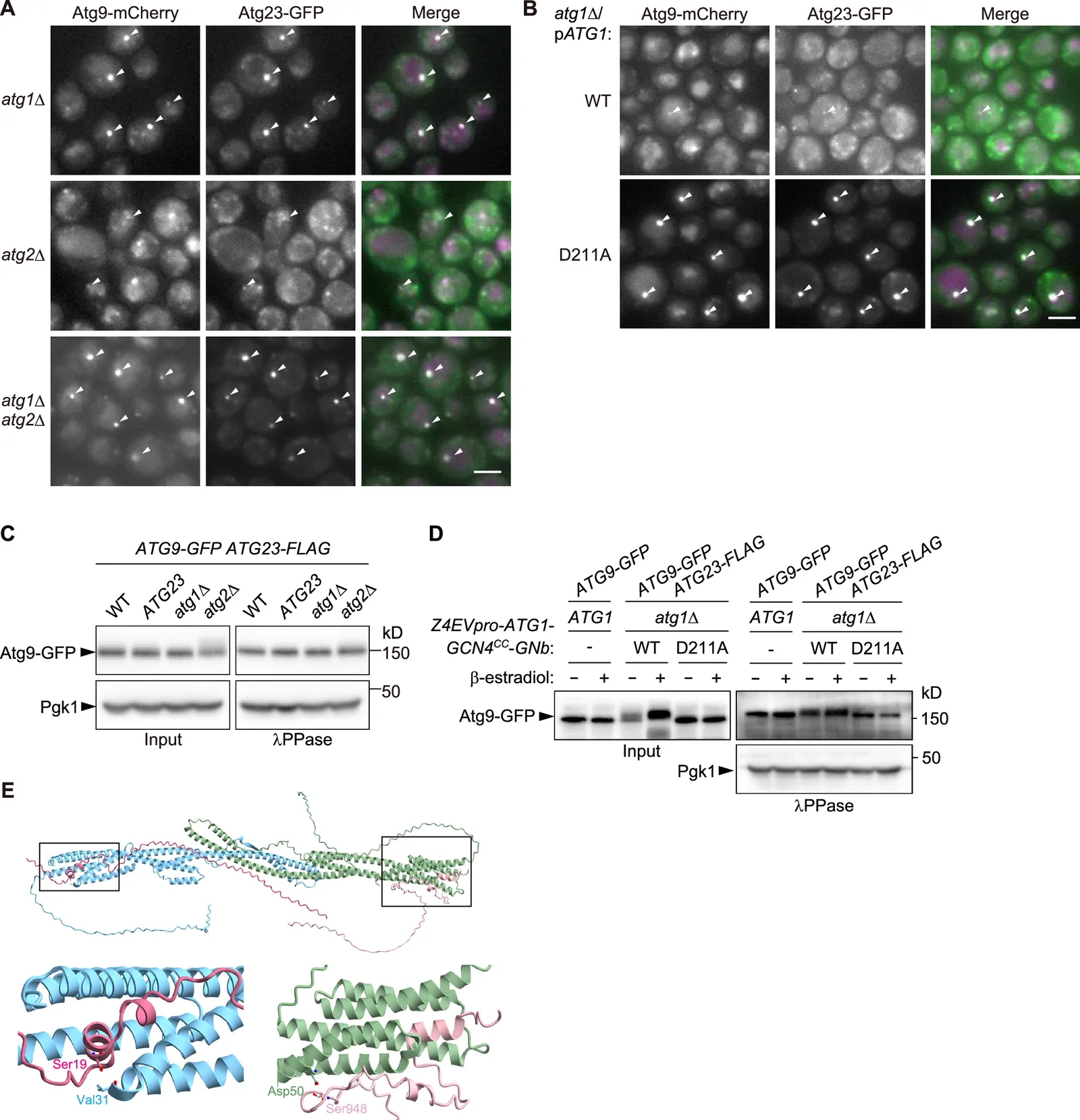
PAS accumulation of Atg9 and Atg23 in cells lacking Atg1 kinase activity. (**A**, **B**) Cells expressing Atg9-mCherry and Atg23-GFP were treated with rapamycin for 2 h and analyzed by fluorescence microscopy. Scale bars, 5 μm. White arrowheads, Atg23-GFP puncta colocalized with high-intensity Atg9-mCherry puncta (Atg9-mCherry localized to the PAS). (**C**) Cells expressing Atg9-GFP and Atg23-FLAG were treated with rapamycin for 2 h, and an aliquot of the cell lysates (Input) was treated with λ phosphatase for 30 min and analyzed by immunoblotting using antibodies against GFP and Pgk1. (**D**) Cells expressing Atg9-GFP and Atg23-FLAG were treated with β-estradiol for 2 h to induce expression of Atg1^WT^-Gcn4^CC^-GNb or Atg1^D211A^-Gcn4^CC^-GNb, and an aliquot of the cell lysates (Input) was treated with λ phosphatase for 30 min and analyzed by immunoblotting using antibodies against GFP and Pgk1. (**E**) AlphaFold 3-predicted structure of the Atg23 dimer in complex with Atg9 fragments (residues 1–50 and 897–997). These fragments are colored magenta and pink, respectively. Source data are available online for this figure.

In fluorescence microscopy of wild-type cells, Atg9-GFP localized to most of the PAS (~ 80%), which were visualized as puncta of Atg17-mCherry (Fig. 1D,F). In contrast, despite its robust association with Atg9 vesicles, Atg23 was only occasionally (~ 30%) observed at the PAS, suggesting that it dissociates from Atg9 vesicles after their arrival at the PAS (Fig. 1E,F). Previous studies reported that Atg9 vesicles accumulate at the PAS in Atg1-deficient cells and that Atg23 forms puncta in these cells (Reggiori et al, 2004) (Fig. 1D–I). We confirmed that puncta of Atg23-GFP formed in cells lacking Atg1 (*atg1*Δ cells) or expressing a kinase-dead mutant of Atg1 (*atg1*^*D211A*^ cells) almost completely colocalized with those of Atg9-mCherry and Atg17-mCherry, suggesting that Atg23-GFP was retained at the PAS together with Atg9 vesicles in these cells (Figs. 1E,F,H,I and EV1A,B). In contrast, Atg23-GFP was not retained at the PAS in cells lacking Atg2 (*atg2*Δ cells), even though Atg9 vesicles accumulated at the PAS, as in *atg1*Δ cells (Figs. 1D–F and EV1A). However, PAS retention of Atg23-GFP was observed when *ATG1* was deleted in *atg2*Δ cells (Fig. EV1A). These results suggest that Atg23 is released from Atg9 vesicles at the PAS in an Atg1 kinase activity-dependent manner.

### Atg1-mediated phosphorylation of Atg9 disrupts its interaction with Atg23

**Figure 2:**
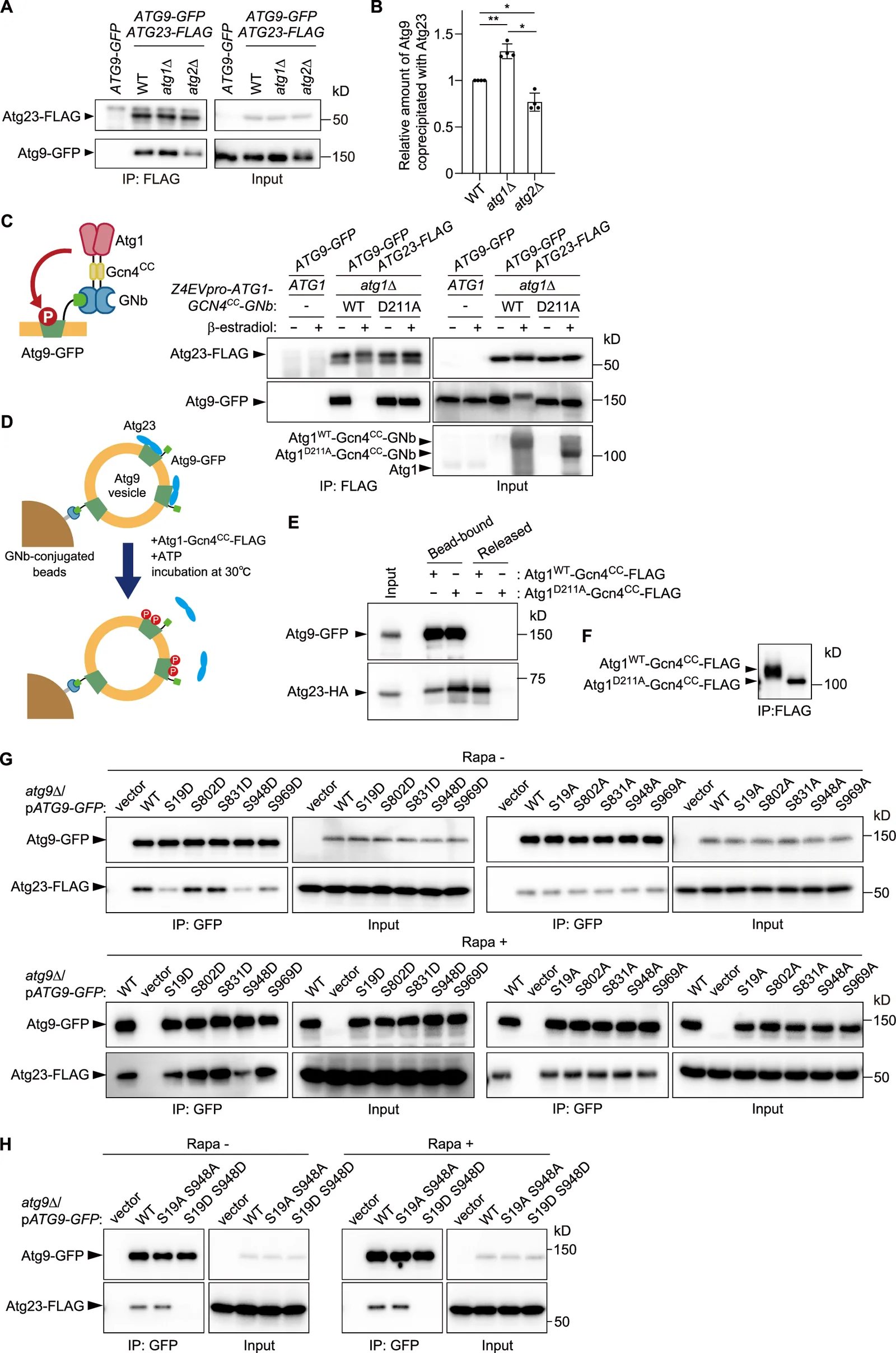
Atg23 dissociates from Atg9 upon Atg1-mediated phosphorylation of Atg9. (**A**) Cells expressing Atg9-GFP and Atg23-FLAG were treated with rapamycin for 2 h, and the cell lysates (Input) were subjected to immunoprecipitation using anti-FLAG antibody. The lysates and immunoprecipitates (IP) were analyzed by immunoblotting using antibodies against FLAG and GFP. (**B**) Quantification of the band intensity of Atg9-GFP coimmunoprecipitated with Atg23-FLAG in the experiments shown in (**A**). Atg9-GFP band intensities were normalized to Atg23-FLAG band intensities in the same samples, and the values are shown relative to the wild-type (set to 1). Bars represent means ± SD. (*n* = 4 independent biological replicates). *P < 0.05, **P < 0.01 (Tukey’s multiple comparisons test): WT vs *atg1*Δ *P*  =  8.7 × 10^−3^; WT vs *atg2*Δ *P*  =  3.5 × 10^−2^; WT vs *atg2*Δ *P*  =  1.1 × 10^−2^. (**C**) Cells expressing Atg9-GFP and Atg23-FLAG were treated with β-estradiol for 2 h to induce expression of Atg1^WT^-Gcn4^CC^-GNb or Atg1^D211A^-Gcn4^CC^-GNb, and the cell lysates (Input) were subjected to immunoprecipitation using anti-FLAG antibody. The lysates and immunoprecipitates (IP) were analyzed by immunoblotting using antibodies against FLAG, GFP, and Atg1. (**D**–**F**) Schematic representation of in vitro phosphorylation of Atg9 by Atg1 on isolated Atg9 vesicles associated with Atg23 (**D**). Atg9 vesicles were isolated from cells expressing Atg9-GFP and Atg23-HA using GNb-conjugated beads (**E**, Input). Atg1^WT^-Gcn4^CC^-FLAG and the D211A mutant were separately isolated using anti-FLAG-antibody-conjugated beads, and the eluates obtained with FLAG peptides were confirmed by immunoblotting with antibodies against FLAG (**F**). These eluates were incubated with the Atg9 vesicle-bound beads in the presence of ATP for 30 min. After bead collection, bead-bound and released fractions were analyzed by immunoblotting with antibodies against GFP and HA. (**G**, **H**) Cells expressing Atg9-GFP and Atg23-FLAG were treated with or without rapamycin for 2 h. Cell lysates (Input) were subjected to immunoprecipitation using GNb-conjugated beads. The immunoprecipitates (IP) were analyzed by immunoblotting with antibodies against FLAG and GFP. Source data are available online for this figure.

The microscopic observations described above suggested that the interaction between Atg23 and Atg9 is weakened by Atg1-mediated phosphorylation. Consistent with this notion, knockout of *ATG1* increased the level of Atg9-GFP coimmunoprecipitated with 6×FLAG-tagged Atg23 (Atg23-FLAG) (Fig. 2A,B). In contrast, in *atg2*Δ cells, the band of Atg9-GFP was upshifted, reflecting the accumulation of phosphorylated Atg9, and its coprecipitation with Atg23-FLAG was less efficient than in wild-type cells. The Atg9 signal appeared reduced in *atg2*Δ cells (Fig. 2A). However, this reduction was abolished by λ phosphatase treatment, showing that the apparent decrease was due to phosphorylation-dependent band smearing rather than a reduction in protein levels (Fig. EV1C). Previous studies showed that Atg9 is phosphorylated by Atg1 (Papinski et al, 2014; Dokládal et al, 2021). To examine whether Atg9 phosphorylation by Atg1 affects its interaction with Atg23, we constructed cells in which Atg9 is forcedly phosphorylated by Atg1. Atg1 becomes constitutively active through autophosphorylation when dimerized by fusion with a coiled-coil region from the transcription factor Gcn4 (Yeh et al, 2011; Landschulz et al, 1988). We added a GFP-binding nanobody (GNb) (Rothbauer et al, 2006) to this construct to generate Atg1^WT^-Gcn4^CC^-GNb, which was expressed under the β-estradiol-inducible promoter *Z4EV* (McIsaac et al, 2013) in cells expressing Atg9-GFP. Through the strong interaction between GFP and GNb, Atg9 was expected to undergo constitutive phosphorylation by Atg1^WT^-Gcn4^CC^-GNb (Fig. 2C). Indeed, upon induction of Atg1^WT^-Gcn4^CC^-GNb expression by β-estradiol, the Atg9-GFP band was markedly upshifted, whereas this band shift was not observed in cells expressing the kinase-inactive Atg1^D211A^-Gcn4^CC^-GNb (Fig. 2C, “Input”). The reduced Atg9-GFP signal in Atg1^WT^-Gcn4^CC^-GNb-expressing cells was also due to phosphorylation-dependent band smearing rather than decreased protein levels, as confirmed by λ phosphatase treatment (Fig. EV1D). In cells without Atg1^WT^-Gcn4^CC^-GNb expression or cells expressing Atg1^D211A^-Gcn4^CC^-GNb, immunoprecipitation of Atg23-FLAG efficiently coprecipitated Atg9-GFP (Fig. 2C). In contrast, coprecipitation of Atg9-GFP with Atg23-FLAG was barely observed in cells expressing Atg1^WT^-Gcn4^CC^-GNb. These results suggest that Atg9 phosphorylation by Atg1 reduces its binding to Atg23.

We further conducted in vitro analysis to examine whether Atg1-mediated phosphorylation can dissociate Atg23 from Atg9 vesicles (Fig. 2D). First, Atg9-GFP vesicles associated with 6×HA-tagged Atg23 (Atg23-HA) were bound to GNb-conjugated beads (Fig. 2E, “Input”). These vesicle-bound beads were incubated with either Atg1^WT^-Gcn4^CC^-FLAG or Atg1^D211A^-Gcn4^CC^-FLAG, which were separately immunoisolated from yeast cell lysates (Fig. 2F), in the presence of ATP to allow Atg9 phosphorylation. The beads were then collected, and proteins that remained bound to the beads and those that were released into the reaction buffer were analyzed by immunoblotting (Fig. 2E). Whereas Atg9-GFP was exclusively recovered from the bead-bound fraction after incubation, a large proportion of Atg23-HA was detected in the released fraction, accompanied by a concomitant decrease in its level in the bead-bound fraction, when the vesicle-bound beads were incubated with Atg1^WT^-Gcn4^CC^-FLAG. In contrast, Atg23-HA remained fully bead-bound after incubation of the beads with Atg1^D211A^-Gcn4^CC^-FLAG. These results suggest that Atg1 can phosphorylate Atg9 bound to Atg23 and disrupt their interaction, resulting in the dissociation of Atg23 from Atg9 vesicles.

A previous study identified Atg9 residues phosphorylated in an Atg1-dependent manner (Papinski et al, 2014; Dokládal et al, 2021), among which we focused on five serine residues located within the Atg1 consensus sequence in regions involved in Atg23 binding (Legakis et al, 2007): Ser19, Ser802, Ser831, Ser948, and Ser969. When each of these residues was replaced with aspartic acid, which potentially mimics a phosphorylated serine residue, a reduction in the coimmunoprecipitation of Atg23 with Atg9 was observed in several mutants, especially the S19D and S948D mutants, both in the presence and absence of rapamycin (Fig. 2G). We also showed that the S19D S948D double mutant almost completely lost its interaction with Atg23 (Fig. 2H). In contrast, alanine substitution of any of the five serine residues in Atg9, including the S19A S948A double mutation, did not affect its interaction with Atg23 (Fig. 2G,H). Structural predictions using AlphaFold 3 (Abramson et al, 2024) suggested that these serine residues are located at the Atg23-binding interface (Fig. EV1E). These results are consistent with the notion that the phosphorylation of Ser19 and Ser948 in Atg9 by Atg1 leads to Atg23 dissociation from Atg9 by directly weakening their interaction.

### Atg23 protects Atg9 vesicles from aberrant fusion during their PAS localization

**Figure EV2:**
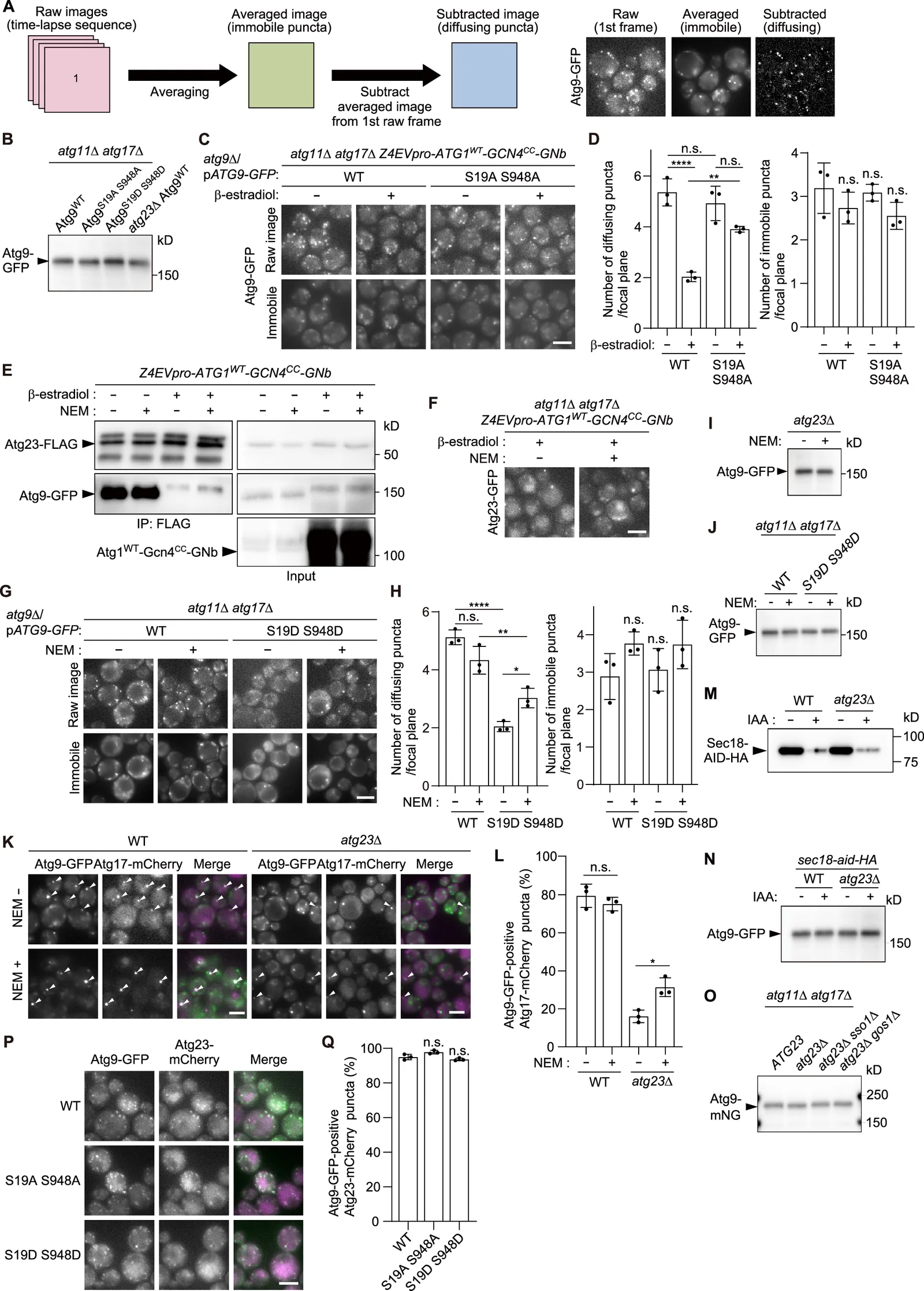
Supporting analyses for Atg23-mediated inhibition of SNARE-dependent fusion of Atg9 vesicles. (**A**) Image processing workflow for quantification of Atg9 vesicles. Time-lapse fluorescence microscopy was performed on cells expressing Atg9-GFP. To extract diffusing Atg9 puncta, 50 frames were stacked and averaged to generate an average intensity projection image representing immobile Atg9-GFP-positive structures such as the Golgi and endosomes. This averaged image was subtracted from the first frame, resulting in a difference image highlighting diffusing puncta. The resulting diffusing puncta image was used to quantify the number of Atg9 vesicles. Details are described in the “Methods” section. (**B**) Immunoblot analysis of Atg9-GFP expression levels in cells used for microscopy in Fig. 3C. The cell lysates were analyzed by immunoblotting using an antibody against GFP. (**C**) *atg11*Δ *atg17*Δ cells expressing Atg9-GFP were treated with β-estradiol for 2 h to induce expression of Atg1^WT^-Gcn4^CC^-GNb and analyzed by fluorescence microscopy as described in Fig. 3A. (**D**) Quantification of the number of diffusing puncta and immobile puncta per focal plane in the experiments shown in (**E**). Bars represent means ± SD (*n* = 3 independent biological replicates). **P < 0.01, ****P < 0.0001, n.s., not significant (Tukey’s multiple comparisons test). For number of Atg9 vesicles, WT β-estradiol- vs WT β-estradiol+ *P* = 7.7 × 10^−5^; WT β-estradiol- vs S19A S948A β-estradiol- *P* = 6.5 × 10^−1^; WT β-estradiol+ vs S19A S948A β-estradiol+ *P* = 3.8 × 10^−3^; S19A S948A β-estradiol- vs S19A S948A β-estradiol+ *P* = 8.9×10^−2^. For number of immobile puncta, WT β-estradiol- vs WT β-estradiol+ *P* = 5.1 × 10^−1^; WT β-estradiol- vs S19A S948A β-estradiol- *P* = 9.9 × 10^−1^; WT β-estradiol- vs S19A S948A β-estradiol+ *P* = 2.6 × 10^−1^. (**E**) Cells expressing Atg9-GFP and Atg23-FLAG were treated with β-estradiol to induce the expression of Atg1^WT^-Gcn4^CC^-GNb for 1.5 h and then with NEM for 30 min. The cell lysates (Input) were subjected to immunoprecipitation using anti-FLAG antibody, and the lysates and the immunoprecipitates (IP) were analyzed by immunoblotting using antibodies against FLAG, GFP, and Atg1. (**F**) Cells expressing Atg23-GFP were treated with β-estradiol and NEM as described in (**E**) and analyzed by fluorescence microscopy. Scale bar, 5 μm. (**G**) *atg11*Δ *atg17*Δ cells expressing Atg9-GFP were treated with NEM for 30 min and analyzed by fluorescence microscopy as described in Fig. 3A. Scale bar, 5 μm. (**H**) Quantification of the number of diffusing puncta and immobile puncta per focal plane in the experiments shown in (**G**). Bars represent means ± SD (*n* = 3 independent biological replicates). **P < 0.01, ****P < 0.0001, n.s., not significant (Tukey’s multiple comparisons test). For number of Atg9 vesicles, WT ΝΕΜ- vs WT NEM + *P* = 7.3 × 10^−2^; WT ΝΕΜ- vs S19D S948D NEM- *P* = 1.5 × 10^−5^; WT ΝΕΜ+ vs S19D S948D NEM + *P* = 5.7 × 10^−3^; S19D S948D ΝΕΜ- vs S19D S948D NEM + *P* = 2.7 × 10^−2^. For number of immobile puncta, WT ΝΕΜ- vs WT NEM + *P* = 2.8 × 10^−1^; WT ΝΕΜ- vs S19D S948D ΝΕΜ- *P* = 9.8 × 10^−1^; WT ΝΕΜ- vs S19D S948D ΝΕΜ + *P* = 3.0 × 10^−1^. (**I**) Immunoblot analysis of Atg9-GFP expression levels in cells used for microscopy in Fig. 3G. The cell lysates were analyzed by immunoblotting using an antibody against GFP. (**J**) Immunoblot analysis of Atg9-GFP expression levels in cells used for microscopy in Fig. EV2G. The cell lysates were analyzed by immunoblotting using an antibody against GFP. (**K**) Cells expressing Atg9-GFP and Atg17-mCherry were treated with rapamycin for 1 h and then with NEM for 30 min and analyzed by fluorescence microscopy. Scale bars, 5 μm. White arrowheads, Atg17-mCherry puncta positive for Atg9-GFP. (**L**) Quantification of the percentage of Atg9-GFP-positive Atg17-mCherry puncta in the experiments shown in (**K**). Bars represent means ± SD (*n* = 3 independent biological replicates). *P < 0.05, n.s., not significant (Tukey’s multiple comparisons test): WT NEM- vs WT NEM + *P* = 7.1 × 10^−1^; *atg23*Δ NEM- vs *atg23*Δ NEM + *P* = 3.4 × 10^−2^. (**M**) Immunoblot analysis of Sec18-AID-HA expression levels in cells used for microscopy in Fig. 3I. Cells expressing Sec18-AID-HA were treated with β-estradiol for 15 min and then with IAA for 2 h, inducing OsTIR1 expression and Sec18-AID-HA degradation, respectively. The levels of Sec18-AID-HA were analyzed by immunoblotting using an antibody against HA. (**N**) Immunoblot analysis of Atg9-GFP expression levels in cells used for microscopy in Fig. 3I. The cell lysates were analyzed by immunoblotting using an antibody against GFP. (**O**) Immunoblot analysis of Atg9-mNeonGreen (Atg9-mNG) expression levels in cells used for microscopy in Fig. 3K. The cell lysates were analyzed by immunoblotting using an antibody against mNeonGreen. (**P**) Cells expressing Atg9-GFP and Atg23-mCherry were treated with rapamycin for 2 h and analyzed by fluorescence microscopy. Scale bar, 5 μm. (**Q**) Quantification of the percentage of Atg23-mCherry puncta positive for Atg9-GFP in the experiments shown in (**P**). Bars represent means ± SD (*n* = 3 independent biological replicates). n.s., not significant (Tukey’s multiple comparisons test): Atg9^WT^ vs Atg9^S19A S948A^ *P* = 5.7 × 10^−2^; Atg9^WT^ vs Atg9^S19D S948D^ *P* = 4.0 × 10^−1^. Source data are available online for this figure.

**Figure 3:**
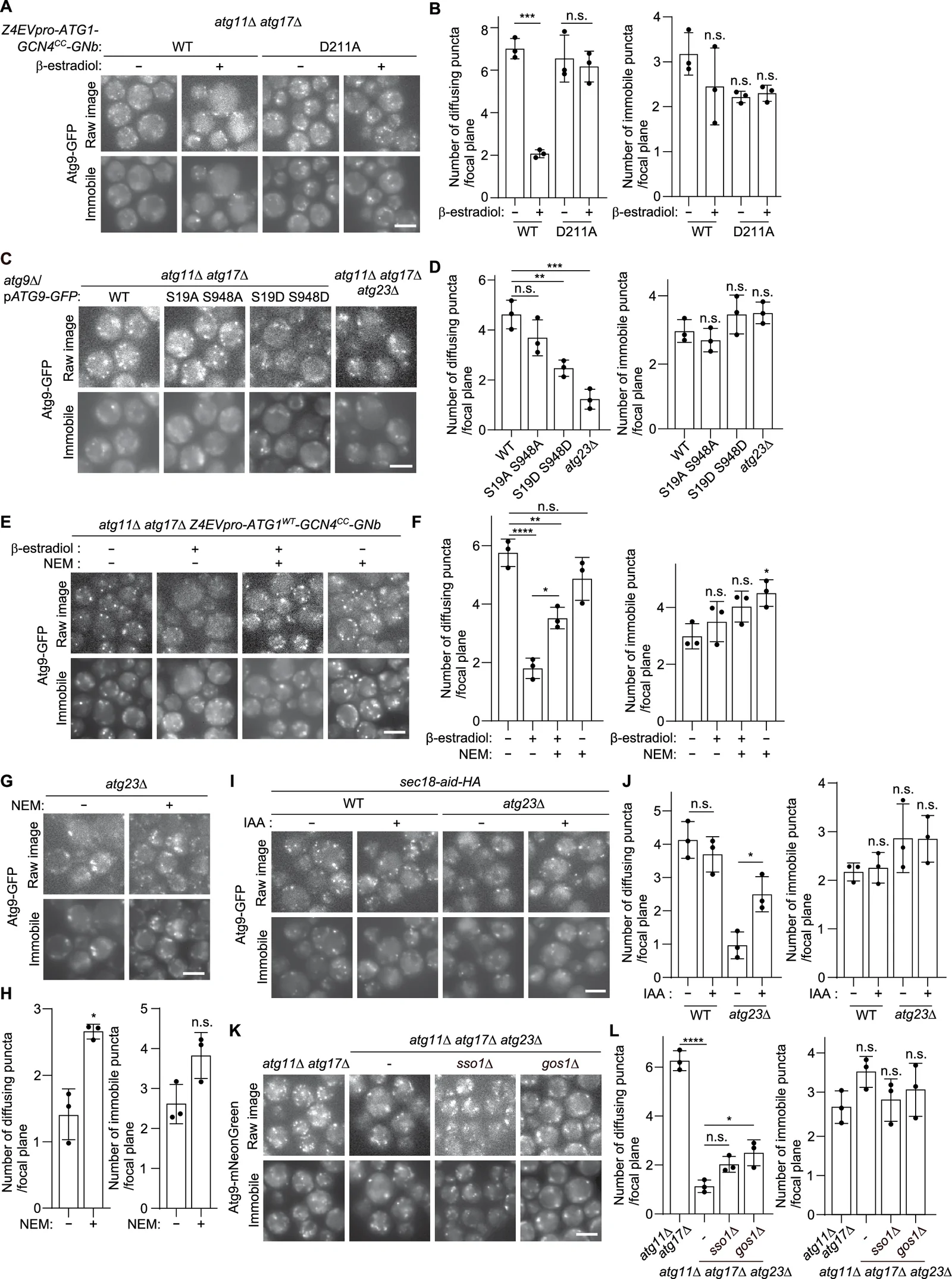
Atg23 prevents SNARE-dependent fusion of Atg9 vesicles with non-autophagic membranes. (**A**) *atg11*Δ *atg17*Δ cells expressing Atg9-GFP were treated with β-estradiol for 2 h to induce expression of Atg1^WT^-Gcn4^CC^-GNb or the D211A mutant and analyzed by fluorescence microscopy. The raw images show the first frame of the original time-lapse imaging, and the images of diffusing puncta were obtained by subtracting immobile puncta from the raw images, and immobile puncta images were obtained by averaging the raw images (see Methods for details). Scale bar, 5 μm. (**B**) Quantification of the number of diffusing puncta and immobile puncta per focal plane in the experiments shown in (**A**). Bars represent means ± SD (*n *= 3 independent biological replicates). ***P < 0.001, n.s., not significant (Tukey’s multiple comparisons test). For the number of diffusing puncta: WT β-estradiol- vs β-estradiol+ *P*  =  1.0 × 10^−4^; D211A β-estradiol- vs β-estradiol+ *P*  =  8.6 × 10^−1^. For the number of immobile puncta: WT β-estradiol- vs β-estradiol+ *P*  =  3.5 × 10^−1^; D211A β-estradiol- vs β-estradiol+ *P*  >  0.99. (**C**) *atg11*Δ *atg17*Δ cells expressing Atg9-GFP were treated with rapamycin for 2 h and analyzed by fluorescence microscopy as described in (**A**). Scale bar, 5 μm. (**D**) Quantification of the number of diffusing puncta and immobile puncta per focal plane in the experiments shown in (**C**). Bars represent means ± SD (*n* = 3 independent biological replicates). **P < 0.01, ***P < 0.001, n.s., not significant (Tukey’s multiple comparisons test). For the number of diffusing puncta: WT vs S19A S948A *P*  =  2.1 × 10^−1^; WT vs S19D S948D *P*  =  4.7 × 10^−3^; WT vs *atg23*Δ *P*  =  2.0 × 10^−4^. For the number of immobile puncta: WT vs S19A S948A *P*  =  8.5 × 10^−1^; WT vs S19D S948D *P*  =  5.0 × 10^−1^; WT vs *atg23*Δ *P*  =  4.3 × 10^−1^. (**E**) *atg11*Δ *atg17*Δ cells expressing Atg9-GFP were treated with β-estradiol for 1.5 h for Atg1^WT^-Gcn4^CC^-GNb expression and then with NEM for 30 min, and analyzed by fluorescence microscopy as described in (**A**). Scale bar, 5 μm. (**F**) Quantification of the number of diffusing puncta and immobile puncta per focal plane in the experiments shown in (**E**). Bars represent means ± SD (*n* = 3 independent biological replicates). *P < 0.05, **P < 0.01, ****P < 0.0001, n.s., not significant (Tukey’s multiple comparisons test). For the number of diffusing puncta: β-estradiol- NEM- vs β-estradiol+ NEM- *P*  = 5.4 × 10^−5^; β-estradiol- NEM- vs β-estradiol+ NEM + *P*  =  2.8 × 10^−3^; β-estradiol- NEM- vs β-estradiol- NEM + *P*  =  2.1 × 10^−1^; β-estradiol+ NEM- vs β-estradiol+ NEM + *P*  =  1.3 × 10^−2^. For the number of immobile puncta: β-estradiol- NEM- vs β-estradiol+ NEM- *P* = 6.7 × 10^−1^; β-estradiol- NEM- vs β-estradiol+ NEM + *P*  =  1.7 × 10^−1^; β-estradiol- NEM- vs β-estradiol- NEM + *P*  =  3.9 × 10^−2^. (**G**) *atg23*Δ cells expressing Atg9-GFP were treated with NEM for 30 min and analyzed by fluorescence microscopy as described in (**A**). Scale bar, 5 μm. (**H**) Quantification of the number of diffusing puncta and immobile puncta per focal plane in the experiments shown in (**G**). Bars represent means ± SD (*n* = 3 independent biological replicates). *P < 0.05, n.s., not significant (unpaired two-tailed Student’s *t* test). For the number of diffusing puncta: *P*  = 2.3 × 10^−2^. For the number of immobile puncta: *P* = 5.1 × 10^−2^. (**I**) Cells expressing Sec18-AID-HA were treated with β-estradiol for 15 min to induce OsTIR1 expression, followed by IAA treatment for 2 h to deplete Sec18-AID-HA. Cells co-expressing Atg9-GFP were then analyzed by fluorescence microscopy as described in (**A**). Scale bar, 5 μm. (**J**) Quantification of the number of diffusing puncta and immobile puncta per focal plane in the experiments shown in (**I**). Bars represent means ± SD (*n* = 3 independent biological replicates). *P < 0.05, n.s., not significant (Tukey’s multiple comparisons test). For the number of diffusing puncta: WT IAA- vs WT IAA + *P* = 7.3 × 10^−1^; *atg23*Δ IAA- vs *atg23*Δ IAA + *P* = 2.5 × 10^−2^. For the number of immobile puncta: WT IAA- vs WT IAA + *P* > 0.99; WT IAA- vs *atg23*Δ IAA- *P* = 3.4 × 10^−1^; WT IAA- vs *atg23*Δ IAA + *P* = 4.3 × 10^−1^. (**K**) Cells expressing Atg9-mNeonGreen were treated with rapamycin for 2 h and analyzed by fluorescence microscopy as described in (**A**). Scale bar, 5 μm. (**L**) Quantification of the number of diffusing puncta and immobile puncta per focal plane in the experiments shown in (**K**). Bars represent means ± SD (*n* = 3 independent biological replicates). *P < 0.05, ****P < 0.0001, n.s., not significant (Tukey’s multiple comparisons test). For the number of diffusing puncta: *atg11*Δ *atg17*Δ vs *atg11*Δ *atg17*Δ *atg23*Δ *P* = 1.1 × 10^−6^; *atg11*Δ *atg17*Δ *atg23*Δ vs *sso1*Δ *P* = 8.7 × 10^−2^; *atg11*Δ *atg17*Δ *atg23*Δ vs *gos1*Δ *P* = 1.2 × 10^−2^. For the number of immobile puncta: *atg11*Δ *atg17*Δ vs *atg11*Δ *atg17*Δ *atg23*Δ *P* = 2.2 × 10^−1^; *atg11*Δ *atg17*Δ vs *sso1*Δ *P* = 9.7 × 10^−1^; *atg11*Δ *atg17*Δ vs *gos1*Δ *P* = 7.3 × 10^−1^. Source data are available online for this figure.

In fluorescence microscopy of *atg11*Δ *atg17*Δ cells, most Atg9-GFP signals are observed as diffusing puncta representing Atg9 vesicles, but these cells also contain immobile puncta representing Atg9 in the Golgi or endosomes (Yamamoto et al, 2012). Therefore, we established a method to more accurately quantify the number of Atg9 vesicles. When fluorescence images of Atg9-GFP are captured at 32 msec/frame, both diffusing and immobile puncta are visualized. Whereas averaging 50 consecutive frames results in the disappearance of diffusing puncta, immobile puncta remained clearly visible. We quantified the number of Atg9 vesicles (per focal plane) by counting diffusing puncta, which remained after subtracting immobile puncta signals obtained by frame averaging from the first frame image that contained both diffusing and immobile puncta (Fig. EV2A) (see “Methods” for details). This method clearly showed that the expression of Atg1^WT^-Gcn4^CC^-GNb, but not that of its kinase-dead version, largely decreased the number of Atg9 vesicles per cell (Fig. 3A,B). Similarly, the number of Atg9 vesicles was decreased by the S19D S948D mutation in Atg9 or disruption of *ATG23*, but not by the S19A S948A mutation (Figs. 3C,D and EV2B). Moreover, Atg9 vesicle number in the S19A S948A mutant cells was insensitive to the expression of Atg1^WT^-Gcn4^CC^-GNb (Fig. EV2C,D). These results suggest that Atg1-mediated phosphorylation of these serine residues in Atg9 decreased the number of Atg9 vesicles. These findings could be interpreted as indicating that artificially disrupting the interaction between Atg9 and Atg23 impaired the formation of Atg9 vesicles, thereby decreasing their number, because Atg23 is involved in their formation. In addition, it was also possible that the loss of this interaction reduced the stability of Atg9 vesicles after their formation, resulting in a decreased vesicle number. Previous studies reported that Atg9 vesicles contain several SNARE proteins, which mediate vesicle fusion events in different membrane trafficking pathways (Sawa-Makarska et al, 2020; Jahn and Scheller, 2006). Thus, we hypothesized that Atg9 vesicles not associated with Atg23 decrease in number due to their SNARE-mediated inappropriate fusion during their localization to the PAS. To test this hypothesis, we first inactivated Sec18, the yeast homolog of the mammalian SNARE-priming factor *N*-ethylmaleimide (NEM)-sensitive factor (NSF) (Mayer et al, 1996; Eakle et al, 1988; Wilson et al, 1989). As described above, induction of Atg1^WT^-Gcn4^CC^-GNb decreased the number of Atg9 vesicles (Fig. 3A,B). However, when these cells were treated with NEM that inhibits Sec18 (Block et al, 1988; Rexach and Schekman, 1991; Baker et al, 1988), the number of Atg9 vesicles was significantly restored (Fig. 3E,F). Importantly, in these cells, NEM treatment did not restore the interaction between Atg9 and Atg23, and Atg23-GFP remained cytoplasmic (Fig. EV2E,F). In addition, NEM treatment of cells not expressing Atg1^WT^-Gcn4^CC^-GNb (i.e., without β-estradiol treatment) did not increase the number of Atg9 vesicles (Fig. 3E,F). A similar increase in vesicle number was observed upon NEM treatment in *atg23*Δ cells and *atg9*^*S19D S948D*^ cells, but not in wild-type cells, without affecting Atg9-GFP levels (Figs. 3G,H and EV2G–J). NEM treatment also increased the PAS localization of Atg9 in *atg23*Δ cells, suggesting that the increased Atg9 vesicles are functional at least in terms of PAS localization. (Fig. EV2K,L).

Furthermore, we employed the auxin-inducible degron (AID) system (Nishimura et al, 2009) to deplete Sec18. To this end, the chromosomal *SEC18* gene was modified to express Sec18 C-terminally tagged with the AID and the 3×HA sequences (Sec18-AID-HA) in cells expressing the F-box protein OsTIR1 under the control of the *Z4EV* promoter to minimize its basal expression and thereby prevent unintended degradation of Sec18-AID-HA. OsTIR1 recognizes the AID tag in the presence of the auxin indole-3-acetic acid (IAA) and directs AID-fused proteins to proteasomal degradation via ubiquitylation. Immunoblotting analysis confirmed that Sec18-AID-HA was depleted upon IAA addition after β-estradiol treatment, without affecting Atg9-GFP levels (Fig. EV2M,N). Fluorescence microscopy revealed that the reduction in Atg9 vesicle number caused by *ATG23* knockout was reversed by Sec18 depletion (Fig. 3I,J). Together, these results support the notion that the decrease in Atg9 vesicles observed in cells lacking Atg23 or the Atg9-Atg23 interaction results not only from defective Atg9 vesicle formation but also from their SNARE-dependent inappropriate fusion.

Finally, in *atg23*Δ cells, we disrupted genes encoding the SNARE proteins Sso1 and Gos1, which localize to Atg9 vesicles and are non-essential for yeast growth (Sawa-Makarska et al, 2020; Aalto et al, 1993; McNew et al, 1998). Knockout of *GOS1* partially but significantly restored the number of Atg9 vesicles in *atg23*Δ cells (Figs. 3K,L and EV2O), suggesting that this SNARE protein is involved in aberrant fusion of Atg9 vesicles that leads to their reduction in number.

Given that the reduction in Atg9 vesicles was modest in *atg9*^*S19D S948D*^ mutant cells compared with *atg23*Δ cells (Fig. 3C,D), even though the interaction of the Atg9^S19D S948D^ mutant with Atg23 was reduced to an undetectable level in coimmunoprecipitation analysis (Fig. 2H), we examined the association of Atg23 with Atg9 vesicles by fluorescence microscopy. The results showed that most Atg9^S19D S948D^ vesicles, as in the case of wild-type Atg9 vesicles, were positive for Atg23 (Fig. EV2P,Q). This observation may account for the apparent discrepancy between the severely impaired interaction of this Atg9 mutant with Atg23 observed in coimmunoprecipitation analysis and its modest phenotypes. The Atg9^S19D S948D^ mutant may not completely lose the interaction with Atg23 in cells. In addition, the membrane-binding ability of Atg23 (Hawkins et al, 2022) may compensate for its association with Atg9 vesicles.

### Atg1-mediated phosphorylation of Atg9 promotes Atg23 release from Atg9 vesicles at the PAS and subsequent recruitment of downstream factors

**Figure 4:**
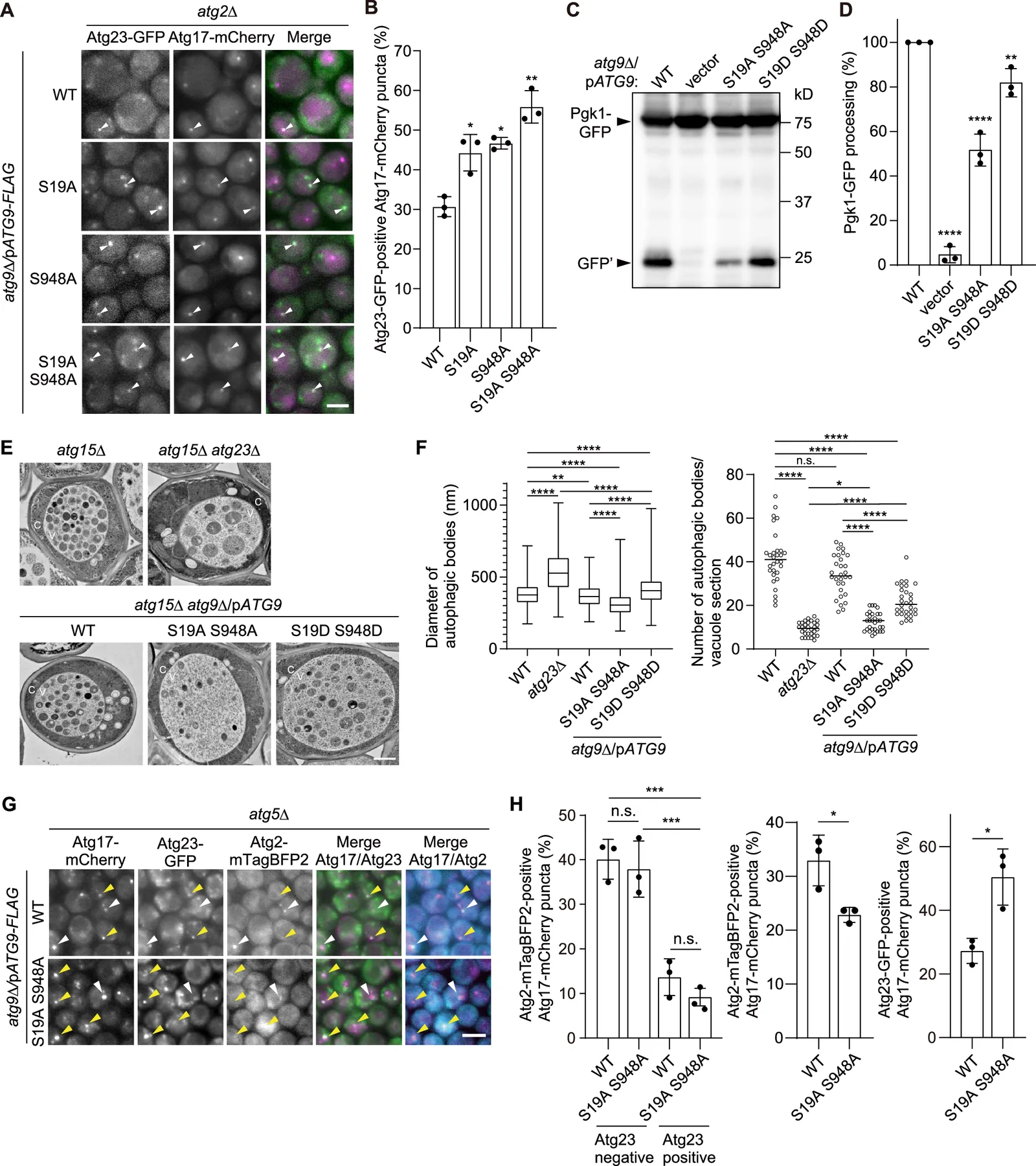
Atg23 dissociation from Atg9 vesicles facilitates Atg2 recruitment to the PAS. (**A**) *atg2*Δ cells expressing Atg23-GFP and Atg17-mCherry were treated with rapamycin for 2 h and analyzed by fluorescence microscopy. Scale bar, 5 μm. White arrowheads, Atg17-mCherry puncta positive for Atg23-GFP. (**B**) Quantification of the percentage of Atg17-mCherry puncta positive for Atg23-GFP in the experiments shown in (**A**). Bars represent means ± SD (*n* = 3 independent biological replicates). *P < 0.05, **P < 0.01 (Tukey’s multiple comparisons test): WT vs S19A *P*  =  3.8 × 10^−3^; WT vs S948A *P*  =  4.9 × 10^−2^; WT vs S19A S948A *P*  =  4.4 × 10^−3^. (**C**) Cells expressing Pgk1-GFP were treated with rapamycin for 4 h and analyzed by immunoblotting using antibodies against GFP. (**D**) The band intensities of Pgk1-GFP and GFP fragments (GFP’) in the experiments shown in (**C**) were measured, and the percentage of GFP fragments relative to the sum of Pgk1-GFP and GFP fragments was calculated as an index of Pgk1-GFP degradation. Bars represent means ± SD (*n* = 3 independent biological replicates) of values relative to WT set to 100. **P < 0.01, ****P < 0.0001 (Tukey’s multiple comparisons test): WT vs vector *P* = 6.7 × 10^−8^; WT vs S19A S948A *P* = 2.2 × 10^−6^; WT vs S19D S948D *P* = 7.3 × 10^−3^. (**E**) *atg15*Δ cells were treated with rapamycin for 24 h. Autophagic bodies accumulated in the vacuole were observed by electron microscopy. C, cytoplasm. V, vacuole. Scale bar, 1 μm. (**F**) Quantification of diameter of autophagic bodies and number of autophagic bodies/vacuole section in the experiments shown in (**E**). Bars represent means ± SD (*n* = 3 independent biological replicates). *P < 0.05, **P < 0.01, ****P < 0.0001, n.s., not significant (Tukey’s multiple comparisons test). For diameter of autophagic bodies: WT vs *atg23*Δ *P* = 4.6 × 10^−9^; WT vs Atg9 WT *P* = 5.8 × 10^−3^; WT vs Atg9 S19A S948A *P* = 4.6 × 10^−9^; WT vs Atg9 S19D S948D *P* = 5.5 × 10^−9^; *atg23*Δ vs Atg9 S19D S948D *P* = 4.6 × 10^−9^; Atg9 WT vs Atg9 S19A S948A *P* = 4.6 × 10^−9^; Atg9 WT vs Atg9 S19D S948D *P* = 4.6 × 10^−9^. For number of autophagic bodies/vacuole section: WT vs *atg23*Δ *P* = 6.0 × 10^−14^; WT vs Atg9 WT *P* = 1.9 × 10^−3^; WT vs Atg9 S19A S948A *P* = 6.0 × 10^−14^; WT vs Atg9 S19D S948D *P* = 1.1 × 10^−13^; *atg23*Δ vs Atg9 S19A S948A *P* = 1.5 × 10^−2^; *atg23*Δ vs Atg9 S19D S948D *P* = 6.8 × 10^−8^; Atg9 WT vs Atg9 S19A S948A *P* = 1.0 × 10^−13^; Atg9 WT vs Atg9 S19D S948D *P* = 1.7 × 10^−7^. (**G**) *atg5*Δ cells expressing Atg17-mCherry, Atg23-GFP, and Atg2-mTagBFP2 were treated with rapamycin for 2 h and analyzed by fluorescence microscopy. Scale bar, 5 μm. White arrowheads, Atg23-GFP-negative Atg17-mCherry puncta. Yellow arrowheads, Atg23-GFP-positive Atg17-mCherry puncta. (**H**) Quantification of the percentage of Atg2-mTagBFP2-positive puncta among Atg23-GFP-negative and -positive Atg17-mCherry puncta (left), the percentage of Atg2-mTagBFP2-positive Atg17-mCherry puncta (middle), and the percentage of Atg23-GFP-positive Atg17-mCherry puncta (right) in the experiments shown in (**G**). Bars represent means ± SD (*n* = 3 independent biological replicates). ***P < 0.001, n.s., not significant (Tukey’s multiple comparisons test) (left): Atg23 negative WT vs Atg23 negative S19A S948A *P* = 9.2 × 10^−1^; Atg23 negative WT vs Atg23 positive WT *P* = 4.0 × 10^−4^; Atg23 negative S19A S948A vs Atg23 positive S19A S948A *P* = 2.0 × 10^−4^; Atg23 positive WT vs Atg23 positive S19A S948A *P* = 6.3 × 10^−3^. *P < 0.05 (unpaired two-tailed Student’s *t* test) (middle and right); in middle, *P* = 4.1 × 10^−2^; in right, *P* = 3.4 × 10^−2^. Source data are available online for this figure.

**Figure EV3:**
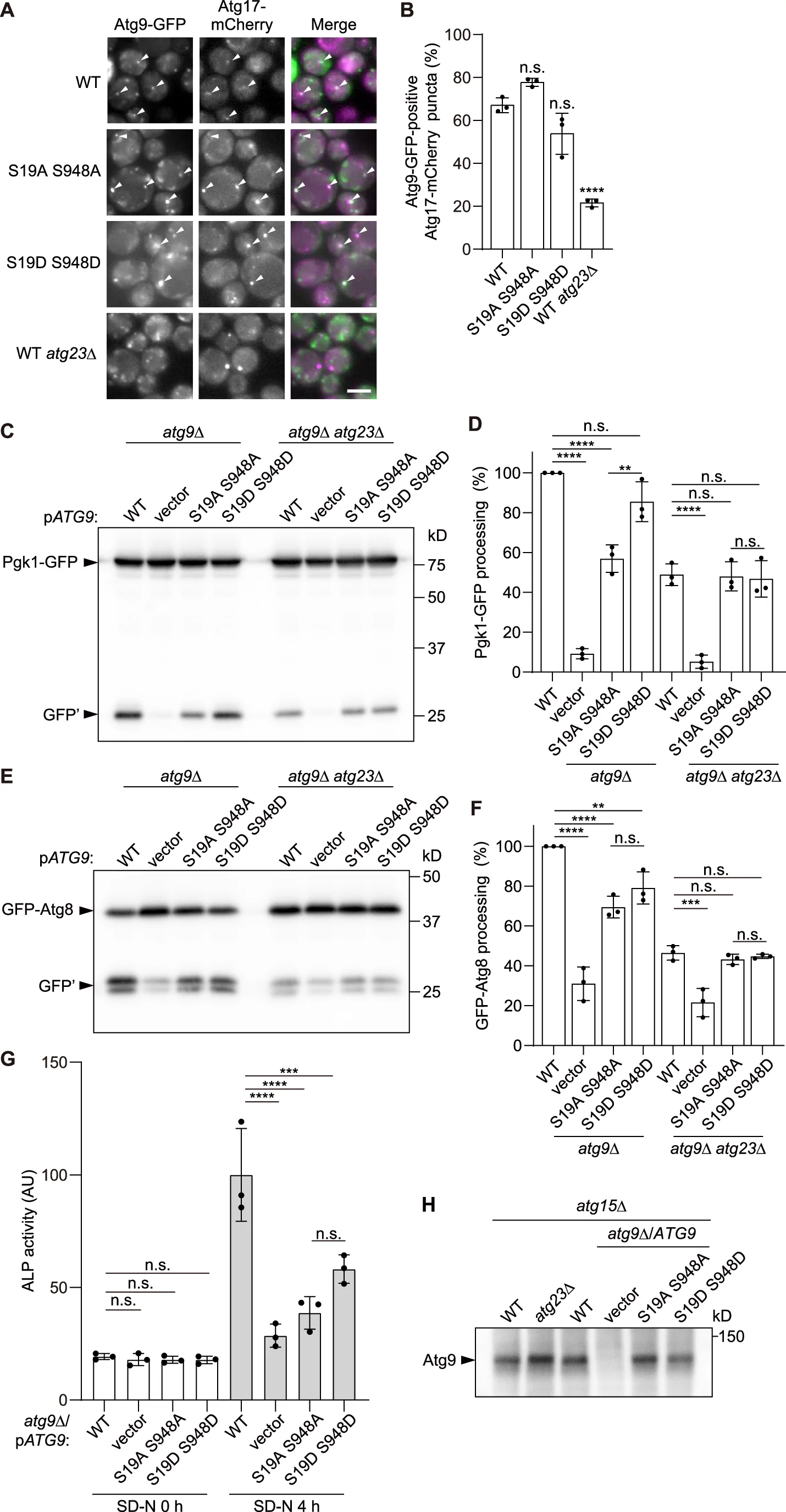
Atg9 phosphorylation is required for efficient autophagy. (**A**) Cells expressing Atg17-mCherry and either Atg9-GFP were treated with rapamycin for 2 h and analyzed by fluorescence microscopy. Scale bars, 5 μm. White arrowheads, Atg17-mCherry puncta positive for Atg9-GFP. (**B**) Quantification of the percentage of Atg17-mCherry puncta positive for Atg9-GFP in the experiments shown in (**A**). Bars represent means ± SD (*n* = 3 independent biological replicates). ****P < 0.0001, n.s., not significant (Tukey’s multiple comparisons test): Atg9^WT^ vs Atg9^S19A S948A^ *P* = 1.3 × 10^−1^; Atg9^WT^ vs Atg9^S19D S948D^ *P* = 5.8 × 10^−2^; Atg9^WT^ vs *atg23*Δ *P* = 2.6 × 10^−5^. (**C**) Cells expressing Pgk1-GFP were treated with rapamycin for 24 h and analyzed by immunoblotting using antibodies against GFP. (**D**) The band intensities of Pgk1-GFP and GFP fragments (GFP’) in the experiments shown in (**C**) were measured, and the percentage of GFP fragments relative to the sum of Pgk1-GFP and GFP fragments was calculated as an index of Pgk1-GFP degradation. Bars represent means ± SD (*n* = 3 independent biological replicates) of values relative to WT set to 100. **P < 0.01, ****P < 0.0001 (Tukey’s multiple comparisons test), n.s., not significant (Tukey’s multiple comparisons test). In *atg9*Δ background, WT vs vector *P* = 2.0 × 10^−10^; WT vs S19A S948A *P* = 9.2 × 10^−6^; WT vs S19D S948D *P* = 1.8 × 10^−1^; S19A S948A vs S19D S948D *P* = 1.1 × 10^−3^. In *atg9*Δ *atg23*Δ background, WT vs vector *P* = 7.6 × 10^−6^; WT vs S19A S948A *P* > 0.99; WT vs S19D S948D *P* > 0.99; S19A S948A vs S19D S948D *P* > 0.99. (**E**) Cells expressing GFP-Atg8 were treated with rapamycin for 24 h and analyzed by immunoblotting using antibodies against GFP. (**F**) The band intensities of GFP-Atg8 and GFP fragments (GFP’) in the experiments shown in (**E**) were measured, and the percentage of GFP fragments relative to the sum of GFP-Atg8 and GFP fragments was calculated as an index of GFP-Atg8 degradation. Bars represent means ± SD (*n* = 3 independent biological replicates) of values relative to WT set to 100. **P < 0.01, ***P < 0.001, ****P < 0.0001, n.s., not significant (Tukey’s multiple comparisons test). In *atg9*Δ background, WT vs vector *P* = 1.0 × 10^−9^; WT vs S19A S948A *P* = 8.0 × 10^−5^; WT vs S19D S948D *P* = 4.4 × 10^−3^; S19A S948A vs S19D S948D *P* = 4.2 × 10^−1^. In *atg9*Δ *atg23*Δ background, WT vs vector *P* = 7.9 × 10^−4^; WT vs S19A S948A *P* = 9.9 × 10^−1^; WT vs S19D S948D *P* > 0.99; S19A S948A vs S19D S948D *P* > 0.99. (**G**) ALP activities in yeast cells expressing the Atg9 mutants. Cells expressing Pho8Δ60 and the Atg9 mutants were grown, and then incubated in SD-N medium for 4 h, and ALP activities in these cells were measured. Bars represent means ± SD (*n* = 3 independent biological replicates) of values relative to WT set to 100. **P < 0.01, ****P < 0.0001, n.s., not significant (Tukey’s multiple comparisons test). In SD-N 0 h, WT vs vector *P* > 0.99; WT vs S19A S948A *P* > 0.99; WT vs S19D S948D *P* > 0.99. In SD-N 4 h, WT vs vector *P* = 3.4 × 10^−7^; WT vs S19A S948A *P* = 2.7 × 10^−6^; WT vs S19D S948D *P* = 3.0 × 10^−4^; S19A S948A vs S19D S948D *P* = 1.5 × 10^−1^. (**H**) Immunoblot analysis of Atg9 expression levels in cells used for microscopy in Fig. 4E. The cell lysates were analyzed by immunoblotting using an antibody. Source data are available online for this figure.

Next, we examined the significance of the Atg1-mediated downregulation of the Atg9-Atg23 interaction at the PAS in autophagosome formation. Since reduced autophagosome formation efficiency can lead to the accumulation of Atg proteins at the PAS, potentially affecting the interpretation of the effects of the Atg9 mutations, we knocked out *ATG2* to block autophagosome formation at a late stage and assessed Atg23 localization to the PAS. We found that Atg23-GFP was more frequently observed at the PAS (Atg17-mCherry puncta) in cells expressing either the phospho-deficient Atg9^S19A^ or Atg9^S948A^ mutant than in those expressing wild-type Atg9 (Fig. 4A,B). The PAS localization frequency of Atg23-GFP was further increased in *atg9*^*S19A S948A*^ double mutant cells, whereas the PAS localization of this Atg9 mutant was not significantly different from that of wild-type Atg9 (Fig. EV3A,B). These results suggest that Atg9 phosphorylation by Atg1 facilitates the release of Atg23 from Atg9 vesicles at the PAS, likely by reducing the interaction between Atg9 and Atg23.

We conducted Pgk1-GFP (Welter et al, 2010) and GFP-Atg8 (Meiling-Wesse et al, 2002) processing assays to evaluate the effects of Atg9 phosphorylation mutations on autophagic activity (Figs. 4C,D and EV3C–F). In these assays, when the cytoplasmic protein Pgk1 or the autophagosomal membrane protein Atg8 tagged with GFP is delivered to the vacuole via autophagy, relatively stable GFP fragments accumulate within the vacuole and can be detected by immunoblotting as a readout of autophagic activity. The results showed that *atg9*^*S19A S948A*^ mutant cells were significantly defective in autophagy, suggesting that dissociation of Atg23 from Atg9 vesicles, promoted by Atg1-mediated phosphorylation of Atg9 at the PAS, is important for efficient progression of autophagy. We also showed that autophagy was defective in cells expressing the Atg9^S19D S948D^ mutant, which exhibited a reduced Atg9-Atg23 interaction (Fig. 2H) and decreased Atg9 vesicle formation (Fig. 3C,D), but the defect was less pronounced than the Atg9^S19A S948A^ mutant (Figs. 4C,D and EV3C–F). Since the PAS localization of the Atg9^S19D S948D^ mutant was not significantly decreased (Fig. EV3A,B), the reduced number of Atg9 vesicles appears to be sufficient to maintain their PAS localization, and this may explain why the defect in autophagy was relatively modest in the Atg9^S19D S948D^ mutant. To further quantify autophagic activity, we performed the Pho8Δ60 alkaline phosphatase (ALP) assay (Fig. EV3G) (Noda et al, 1995). Consistent with the results obtained in the GFP processing assays, both *atg9*^S19A S948A^ and *atg9*^S19D S948D^ cells showed reduced autophagic activity. We also performed the GFP processing assays in the *atg23*Δ background to determine whether the effects of the Atg9 mutations depend on Atg23. In both assays, while *ATG23* knockout significantly reduced autophagic activity, neither mutation showed an additional effect in the *atg23*Δ background, consistent with the notion that these mutations affect autophagy through altered interaction with Atg23 (Fig. EV3C–F).

We further analyzed the size and number of autophagosomes formed in the Atg9 mutants by electron microscopy. For this purpose, we disrupted *ATG15*, which encodes a vacuolar lipase responsible for the disintegration of autophagic bodies, inner autophagosomal membrane vesicles released into the vacuole upon autophagosome-vacuole fusion, to accumulate autophagic bodies, whose size and number reflect those of autophagosomes (Takeshige et al, 1992; Epple et al, 2001; Teter et al, 2001; Watanabe et al, 2023; Kagohashi et al, 2023) (Figs. 4E,F and EV3H). The results showed that compared with cells expressing wild-type Atg9, cells expressing the Atg9^S19A S948A^ mutant contained smaller and fewer autophagic bodies. In the Atg9^S19D S948D^ mutant, the size of autophagic bodies was slightly larger and the number was reduced. Similarly, *ATG23* deletion also caused an increase in size and a decrease in number of autophagic bodies, consistent with the fact that Atg9^S19D S948D^ is defective in its interaction with Atg23. Thus, although the two Atg9 mutants showed opposite effects on autophagic body size, these results suggest that the reduced autophagic activity observed above is due to impaired autophagosome formation in these mutant cells.

**Figure EV4:**
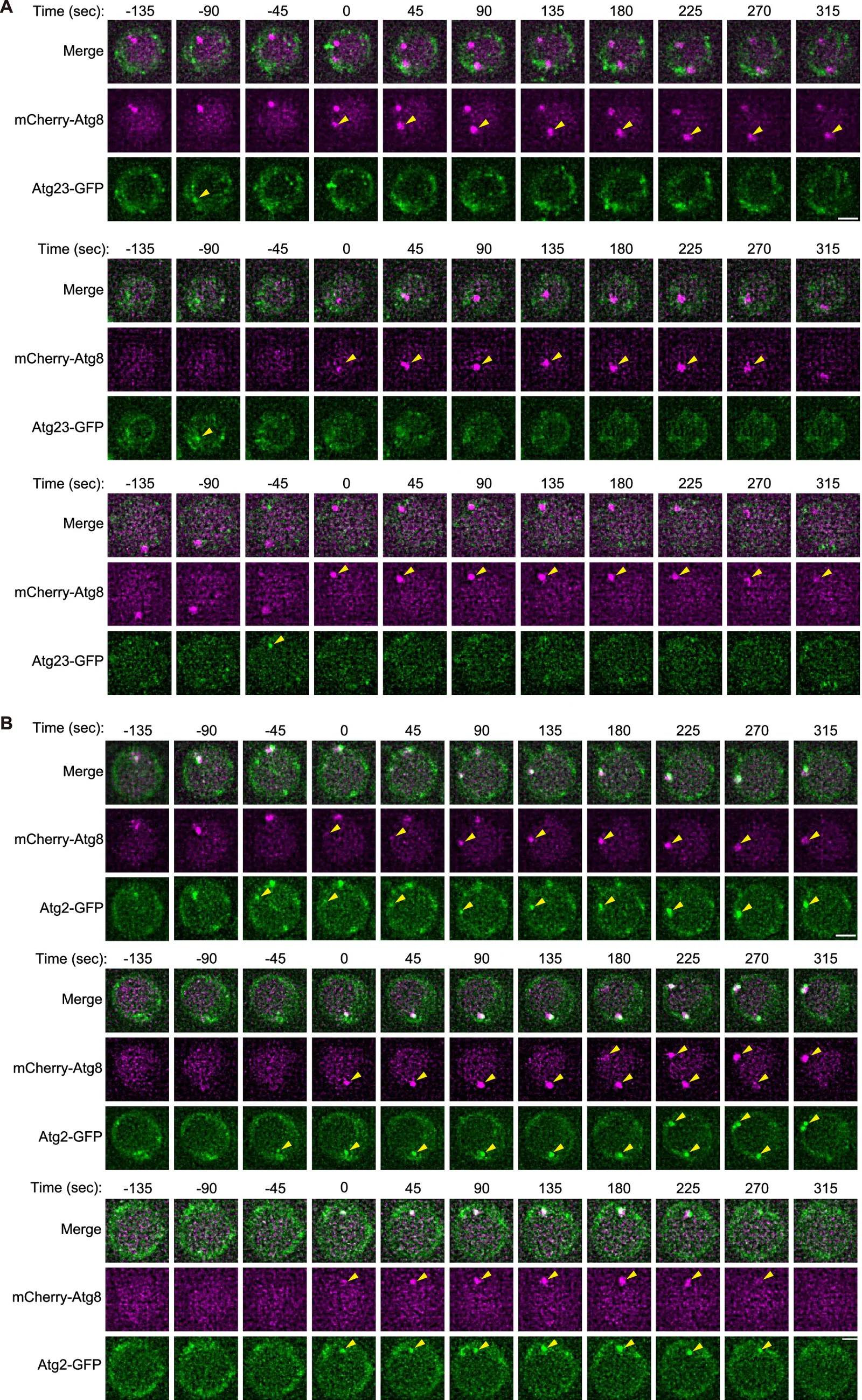
Atg23 localizes to the PAS prior to Atg2 and dissociates earlier. (**A**, **B**) Cells expressing mCherry-Atg8 and Atg23-GFP or Atg2-GFP were treated with rapamycin for 2 h and analyzed by fluorescence microscopy. Time-lapse images were acquired at 45-s intervals for 30 min. Scale bar, 2 μm. Yellow arrowheads indicate mCherry-Atg8, Atg23-GFP, or Atg2-GFP puncta. Source data are available online for this figure.

We next sought to address how the delayed release of Atg23 from Atg9 vesicles at the PAS interferes with autophagosome formation and examined the PAS localization of Atg2, one of the downstream factors localizing to the PAS following Atg9 vesicles, in *atg9*^*S19A S948A*^ cells. To this end, we constructed cells expressing Atg23-GFP, Atg2-mTagBFP2, and Atg17-mCherry to allow simultaneous observation of Atg23 and Atg2 localization to the PAS (Atg17-mCherry puncta) (Fig. 4G). Fluorescence microscopy analysis of these cells revealed that, in both wild-type and *atg9*^*S19A S948A*^ cells, Atg2 localized to Atg23-negative PASs more frequently than to Atg23-positive PASs, suggesting that the retention of Atg23 at the PAS impedes the recruitment of Atg2 (Fig. 4H, left). Although this frequency was comparable between the two strains, the overall frequency of Atg2 localization to the PAS was reduced in *atg9*^*S19A S948A*^ cells (Fig. 4H, middle) because these cells have a higher proportion of Atg23-positive PASs (Fig. 4A,B,H, right). To further examine the dynamics of Atg23 and Atg2 at the PAS, we performed time-lapse imaging of cells expressing mCherry-Atg8 together with either Atg23-GFP or Atg2-GFP at 45 s intervals, following a previously described approach (Li et al, 2025). In this study, we defined the appearance of the mCherry-Atg8 punctum as time 0 s. Atg23 puncta appeared 45–90 s before mCherry-Atg8 and disappeared in the next frame (Fig. EV4A), whereas Atg2 puncta appeared ~45 s before or concomitantly with mCherry-Atg8 and remained colocalized thereafter (Fig. EV4B). These observations support a model in which Atg23 dissociates from the PAS (Atg9 vesicles) prior to Atg2 recruitment.

**Figure EV5:**
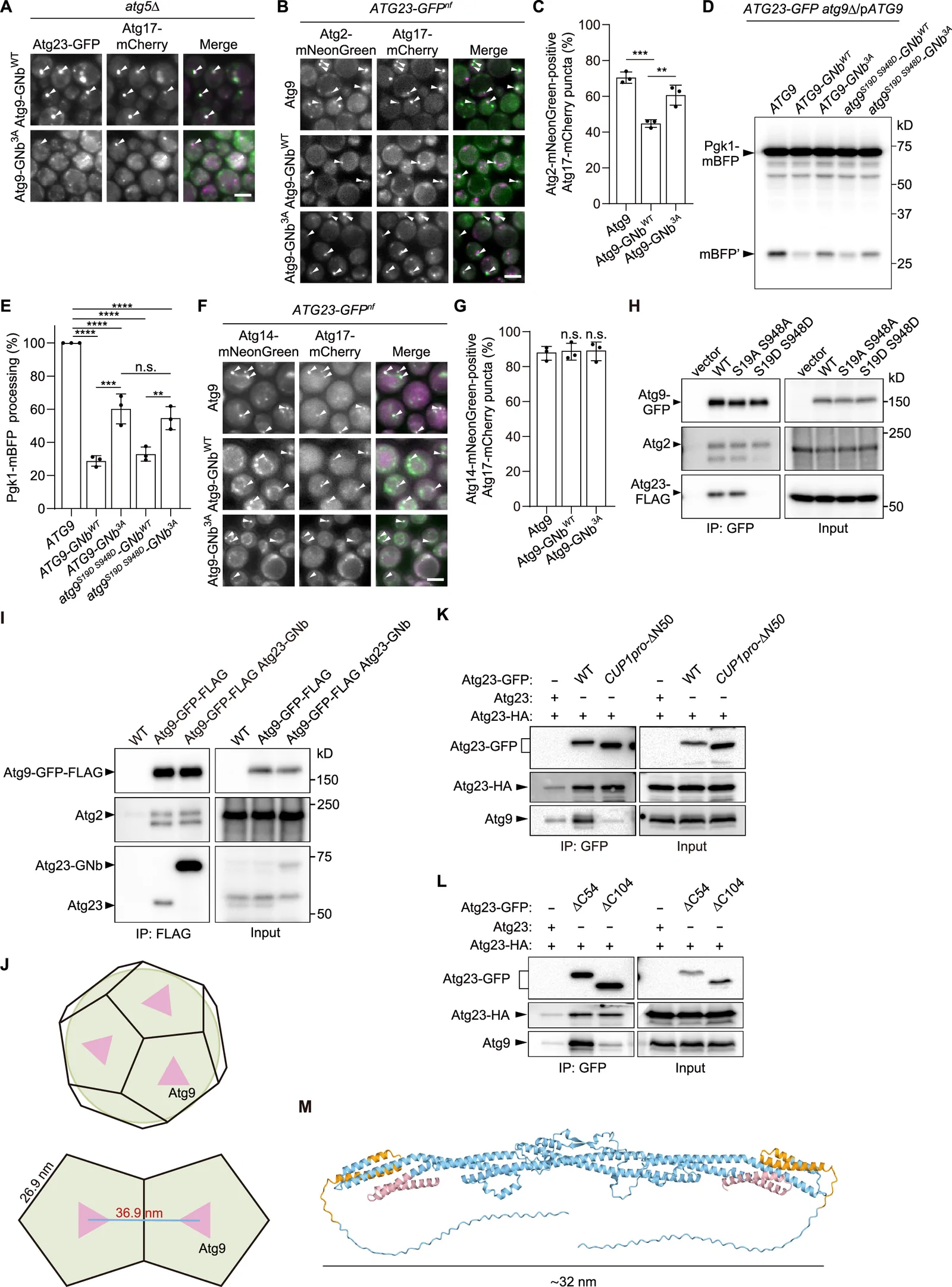
Supporting analyses for Atg23-mediated inhibition of Atg2 recruitment. (**A**) *atg5*Δ cells expressing Atg23-GFP, Atg17-mCherry, and either Atg9-GNb^WT^ or the 3 A mutant were treated with rapamycin for 2 h and analyzed by fluorescence microscopy. Scale bar, 5 μm. White arrowheads, Atg23-GFP-positive Atg17-mCherry puncta. (**B**) Cells expressing Atg2-mNeonGreen, Atg17-mCherry, Atg23-GFP^nf^, and either non-tagged Atg9, Atg9-GNb^WT^, or the 3 A mutant were treated with rapamycin for 2 h and analyzed by fluorescence microscopy. Scale bar, 5  μm. White arrowheads, Atg2-mNeonGreen-positive Atg17-mCherry puncta. (**C**) Quantification of the percentage of Atg2-mNeonGreen-positive Atg17-mCherry puncta in the experiments shown in (**B**). Bars represent means ± SD (*n* = 3 independent biological replicates). **P < 0.01, ***P < 0.001; n.s., not significant (Tukey’s multiple comparisons test): Atg9 vs Atg9-GNb^WT^ *P* = 4.8 × 10^−4^; Atg9 vs Atg9-GNb^3A^ *P* = 4.8 ×  10^−2^. (**D**) Cells expressing Pgk1-mTagBFP2 were treated with rapamycin for 4 h and analyzed by immunoblotting using antibodies against mTagBFP2. (**E**) The band intensities of Pgk1-mTagBFP2 and mTagBFP2 fragments (mBFP’) in the experiments shown in (**D**) were measured, and the percentage of mTagBFP2 fragments relative to the sum of Pgk1-mTagBFP2 and mTagBFP2 fragments was calculated as an index of Pgk1-mTagBFP2 degradation. Bars represent means ± SD (*n* = 3 independent biological replicates) of values relative to WT set to 100. **P < 0.01, ***P < 0.001, ****P < 0.0001, n.s., not significant (Tukey’s multiple comparisons test): Atg9^WT^ vs Atg9^WT^-GNb^WT^ *P* = 2.0 × 10^−7^; Atg9^WT^ vs Atg9^WT^-GNb^3A^ *P* = 4.4 × 10^−5^; Atg9^WT^ vs atg9^S19D S948D^-GNb^WT^ *P* = 3.5 × 10^−7^; Atg9^WT^ vs atg9^S19D S948D^-GNb^3A^ *P* = 1.6 × 10^−3^; Atg9^WT^-GNb^WT^ vs Atg9^WT^-GNb^3A^ *P* = 3.3 × 10^−4^; Atg9^WT^-GNb^3A^ vs atg9^S19D S948D^-GNb^3A^ *P* = 7.4 × 10^−1^; Atg9^S19D S948D^-GNb^WT^ vs Atg9^S19D S948D^-GNb^3A^ *P* = 5.7 × 10^−3^. (**F**) Cells expressing Atg14-mNeonGreen, Atg17-mCherry, Atg23-GFP^nf^, and either non-tagged Atg9, Atg9-GNb^WT^, or the 3A mutant were treated with rapamycin for 2 h and analyzed by fluorescence microscopy. Scale bar, 5 μm. White arrowheads, Atg14-mNeonGreen-positive Atg17-mCherry puncta. (**G**) Quantification of the percentage of Atg14-mNeonGreen-positive Atg17-mCherry puncta in the experiments shown in (**F**). Bars represent means ± SD (*n* = 3 independent biological replicates). n.s., not significant (Tukey’s multiple comparisons test): Atg9 vs Atg9-GNb^WT^ *P* = 9.6 × 10^−1^; Atg9 vs Atg9-GNb^3A^ *P* = 9.5 × 10^−1^. (**H**) Cells expressing Atg9-GFP and Atg23-FLAG were treated with rapamycin for 2 h, and the cell lysates (Input) were subjected to immunoprecipitation using GNb-conjugated beads. The immunoprecipitates (IP) were analyzed by immunoblotting with antibodies against FLAG, GFP and Atg2. (**I**) Cells expressing Atg9-GFP and Atg23-FLAG were treated with rapamycin for 2 h, and the cell lysates (Input) were subjected to immunoprecipitation using anti-FLAG antibody-conjugated beads. The immunoprecipitates (IP) were analyzed by immunoblotting with antibodies against FLAG, GFP and Atg2. (**J**) Approximated Atg9 vesicle model for estimating the distance between Atg9 trimers on the vesicle surface (see Discussion for details). (**K**, **L**) Cells expressing Atg23-GFP and Atg23-HA were treated with rapamycin for 2 h. The cell lysates (Input) were subjected to immunoprecipitation using GNb-conjugated beads, and the immunoprecipitates (IP) were analyzed by immunoblotting using antibodies against GFP, HA, and Atg9. The Atg23 ΔN50 mutant was expressed under the *CUP1* promoter without CuSO_4_ so that its protein level was comparable to that of the wild-type. The results showed that coimmunoprecipitation of Atg9 was markedly reduced in the ΔN50 (residues 1–50) and ΔC104 (residues 350–453) mutants but not in the ΔC54 (residues 400–453) mutant, suggesting that the N-terminal 50 residues and the C-terminal region encompassing residues 350–399 are involved in the interaction with Atg9. In contrast, coprecipitation of Atg23-HA was not affected by these deletions, suggesting that both mutations retain normal homodimerization ability. (**M**) The AlphaFold3-predicted structure of the Atg23 dimer. Atg23 residues 1–50 and 350–399, which are important for interaction with Atg9, are colored pink and orange, respectively. Source data are available online for this figure.

**Figure 5:**
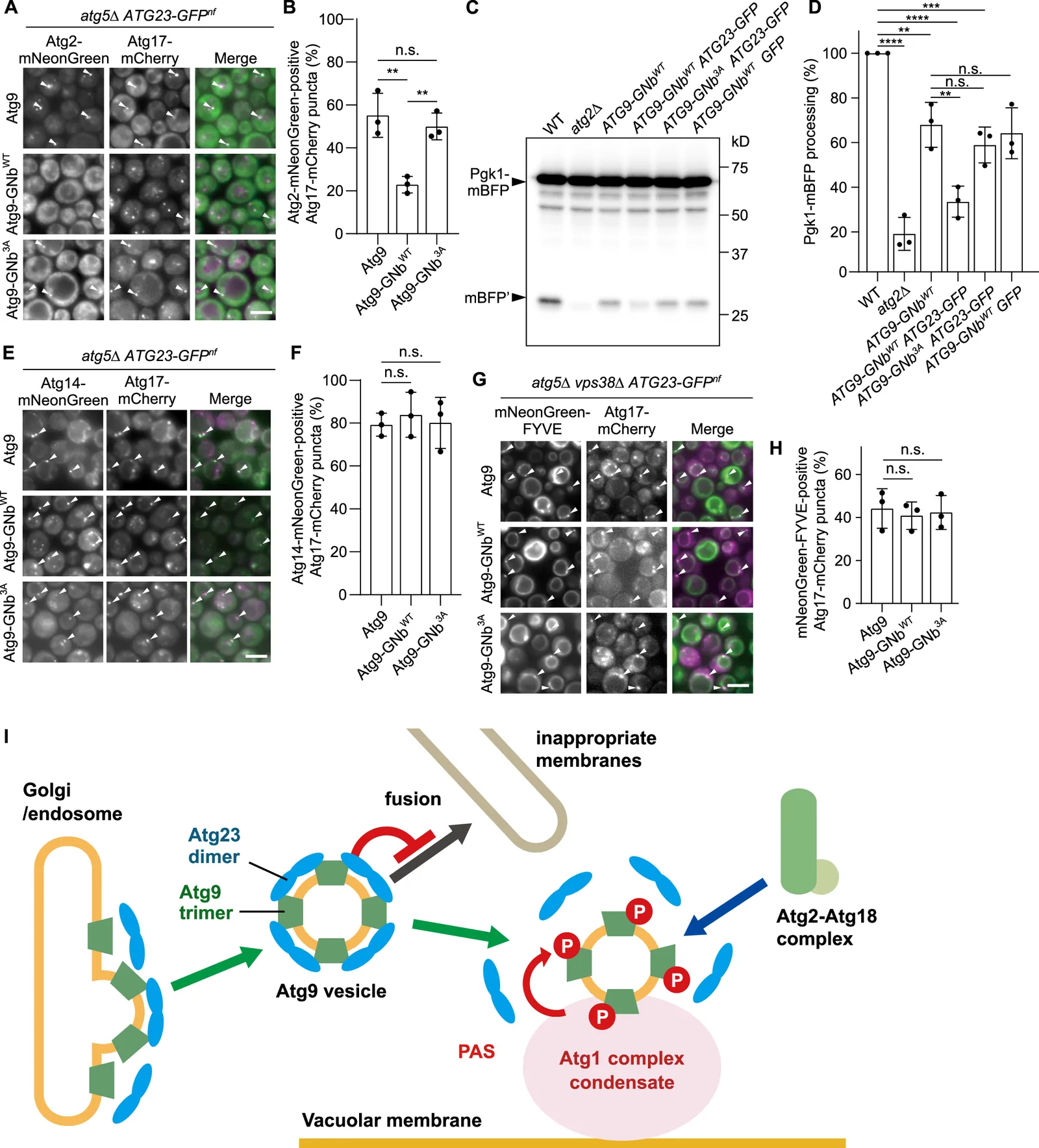
Persistent association of Atg23 with Atg9 impairs Atg2 recruitment to the PAS. (**A**) *atg5*Δ cells expressing Atg2-mNeonGreen, Atg17-mCherry, Atg23-GFP^nf^, and either non-tagged Atg9, Atg9-GNb^WT^, or the 3A mutant were treated with rapamycin for 2 h and analyzed by fluorescence microscopy. Scale bar, 5 μm. White arrowheads, Atg2-mNeonGreen-positive Atg17-mCherry puncta. (**B**) Quantification of the percentage of Atg2-mNeonGreen-positive Atg17-mCherry puncta in the experiments shown in (**A**). Bars represent means ± SD (*n* = 3 independent biological replicates). **P < 0.01; n.s., not significant (Tukey’s multiple comparisons test): Atg9 vs Atg9-GNb^WT^ *P* = 2.6 × 10^−3^; Atg9 vs Atg9-GNb^3A^ *P* = 6.6 × 10^−1^; Atg9-GNb^WT^ vs Atg9-GNb^3A^ *P* = 5.9 × 10^−3^. (**C**) Cells expressing Pgk1-mTagBFP2 were treated with rapamycin for 4 h and analyzed by immunoblotting using antibodies against mTagBFP2. (**D**) The band intensities of Pgk1-mTagBFP2 and mTagBFP2 fragments (mBFP’) in the experiments shown in (**C**) were measured, and the percentage of mTagBFP2 fragments relative to the sum of Pgk1-mTagBFP2 and mTagBFP2 fragments was calculated as an index of Pgk1-mTagBFP2 degradation. Bars represent means ± SD (*n* = 3 independent biological replicates) of values relative to WT set to 100. *P < 0.05, **P < 0.01, ***P < 0.001, ****P < 0.0001, n.s., not significant (Tukey’s multiple comparisons test): WT vs *atg2*Δ *P* = 5.0 × 10^−7^; WT vs Atg9-GNb^WT^ *P* = 4.3 × 10^−3^; WT vs Atg9-GNb^WT^ Atg23-GFP *P* = 4.2 × 10^−6^; WT vs Atg9-GNb^3A^ Atg23-GFP *P* = 5.3 × 10^−4^; WT vs Atg9-GNb^WT^ GFP *P* = 1.7 × 10^−3^; Atg9-GNb^WT^ vs Atg9-GNb^WT^ Atg23-GFP *P* = 2.4 × 10^−3^; Atg9-GNb^WT^ vs Atg9-GNb^3A^ Atg23-GFP *P* = 7.5 × 10^−1^; Atg9-GNb^WT^ vs Atg9-GNb^WT^ GFP *P* = 9.9 × 10^−1^. (**E**) *atg5*Δ cells expressing Atg14-mNeonGreen, Atg17-mCherry, Atg23-GFP^nf^, and either non-tagged Atg9, Atg9-GNb^WT^, or the 3A mutant were treated with rapamycin for 2 h and analyzed by fluorescence microscopy. Scale bar, 5 μm. White arrowheads, Atg14-mNeonGreen-positive Atg17-mCherry puncta. (**F**) Quantification of the percentage of Atg14-mNeonGreen-positive Atg17-mCherry puncta in the experiments shown in (**E**). Bars represent means ± SD (*n* = 3 independent biological replicates). n.s., not significant (Tukey’s multiple comparisons test): Atg9 vs Atg9-GNb^WT^ *P* = 8.3 × 10^−1^; Atg9 vs Atg9-GNb^3A^ *P* > 0.99. (**G**) *atg5*Δ *vps38*Δ cells expressing mNeonGreen-FYVE, Atg17-mCherry, Atg23-GFP^nf^, and either non-tagged Atg9, Atg9-GNb^WT^, or the 3A mutant were treated with rapamycin for 2 h and analyzed by fluorescence microscopy. Scale bar, 5 μm. White arrowheads, mNeonGreen-FYVE-positive Atg17-mCherry puncta. (**H**) Quantification of the percentage of mNeonGreen-FYVE-positive Atg17-mCherry puncta in the experiments shown in (**G**). Bars represent means ± SD (*n* = 3 independent biological replicates). n.s., not significant (Tukey’s multiple comparisons test): Atg9 vs Atg9-GNb^WT^ *P* = 8.6 × 10^−1^; Atg9 vs Atg9-GNb^3A^ *P* = 9.6 × 10^−1^. (**I**) Atg1-dependent association and dissociation of Atg23 on Atg9 vesicles. Atg23 interacts with Atg9 in the Golgi apparatus or endosomes to form Atg9 vesicles and remains associated with Atg9 after vesicle formation. These vesicles diffuse through the cytoplasm and are captured by Atg13, a subunit of the Atg1 kinase complex that organizes the PAS scaffold. During this localization to the PAS, Atg23 on Atg9 vesicles prevents their aberrant membrane fusion, a process corresponding to vesicle coating, thereby ensuring efficient localization of Atg9 vesicles to the PAS. Upon arrival at the PAS, Atg1, which is specifically activated at this site, phosphorylates Atg9 to promote the dissociation of Atg23 from the vesicles. This vesicle uncoating is required for downstream events at the PAS, including the recruitment of the Atg2-Atg18 complex. Source data are available online for this figure.

Atg2 directly interacts with Atg9, and this interaction is important for the localization of Atg2 to the PAS (Wang et al, 2001; Gómez-Sánchez et al, 2018). We hypothesized that Atg23 sterically hinders the binding of Atg2 to Atg9, thereby blocking its PAS recruitment. To test this hypothesis, we modified the genomic *ATG9* and *ATG23* genes to express GNb- and GFP-tagged proteins, respectively, and examined how artificial binding of Atg23 to Atg9 through the tight GNb-GFP interaction affects the PAS localization of Atg2. First, we confirmed that Atg23-GFP accumulated at almost all PASs in these cells, and that this accumulation was largely decreased by the GNb mutations S33A, R35 A, and Y37A (GNb^3A^), which impair GNb binding to GFP (Araki et al, 2013) (Fig. EV5A). We then replaced the GFP fused to Atg23 with a variant containing the T65A Y66A G67A mutations (GFP^nf^) (Tanaka et al, 2014), which abolish its fluorescence without affecting recognition by GNb, to enable the use of green fluorescence for observing Atg2 tagged with mNeonGreen, which does not bind to GNb. Additionally, we knocked out *ATG5* to block autophagosome formation at a downstream step. *atg5*Δ cells expressing Atg9-GNb^WT^ and Atg23-GFP^nf^ exhibited a significant decrease in the localization of Atg2-mNeonGreen to Atg17-mCherry puncta compared with cells expressing non-tagged Atg9, and this effect was rarely observed in cells expressing Atg9-GNb^3A^ (Fig. 5A,B). A similar result was also obtained in cells with intact *ATG5* (Fig. EV5B,C). These results support the idea that Atg23 binding to Atg9 interferes with Atg2 localization to the PAS. We also performed Pgk1 degradation assay using mTagBFP2 instead of GFP, and the results showed that these cells had autophagic activity consistent with the PAS localization of Atg2; the combination of Atg9-GNb^WT^ and Atg23-GFP significantly reduced this activity (Fig. 5C,D). However, we showed that Atg9-GNb^WT^ containing the S19D S948D mutation also showed an inhibitory effect on autophagic activity when coexpressed with Atg23-GFP, whereas this effect was not observed with Atg9^S19AD S948D^-GNb^3A^ (Fig. EV5D). Thus, the inhibitory effect observed in the Atg23-GFP–Atg9-GNb system does not require direct Atg23 binding to Atg9 and can be explained by the steric hindrance effect of Atg23 rather than by secondary mechanisms such as Atg23-induced conformational changes in Atg9.

Atg2 forms a complex with the phosphatidylinositol 3-phosphate (PI3P)-binding protein Atg18, and its PAS recruitment involves Atg18 binding to PI3P produced by PI3KCI, which localizes to the PAS following Atg9 vesicles (Suzuki et al, 2007; Obara et al, 2008; Rieter et al, 2013). We examined the PAS localization of PI3KCI using its component Atg14 fused to mNeonGreen, and showed that this step was not affected in *atg5*Δ cells expressing Atg9-GNb^WT^ and Atg23-GFP^nf^ compared with those expressing non-tagged Atg9 or Atg9-GNb^3A^ (Fig. 5E,F). In addition, the accumulation of PI3P at the PAS was monitored using the PI3P probe mNeonGreen-2×FYVE (mNeonGreen-FYVE), in which two FYVE domains that specifically bind to PI3P are fused in tandem to mNeonGreen (Fig. 5G,H). Since this probe also stains PI3P-enriched endosomes, which are often located near the PAS, it is challenging to distinguish its signals at the PAS from those in endosomes. To eliminate these endosomal signals, we knocked out *VPS38*, which encodes a subunit responsible for the endosomal localization of PI3KC (PI3KCII) and PI3P production in endosomes (Kihara et al, 2001; Obara et al, 2006). Consistent with the unchanged PAS localization of PI3KCI (Figs. 5E,F and EV5F,G), the results suggested that forced Atg23 binding to Atg9 did not significantly change PI3P levels at the PAS (Fig. 5G,H). Thus, these results suggest that, unless Atg23 dissociates from Atg9, steps downstream of PI3P production at the PAS are retarded, consistent with a model in which Atg23 impedes Atg2 recruitment to the PAS. Finally, we examined whether the interaction between Atg9 and Atg2 is blocked when Atg23 remained associated with Atg9. The levels of Atg2 coprecipitated with Atg9 were not changed in either the S19A S948A or S19D S948D mutant (Fig. EV5H) or in cells co-expressing Atg9-GFP and Atg23-GNb (Fig. EV5I). These results suggest that the reduced recruitment of Atg2 to the PAS is unlikely to result from inhibition of the Atg9-Atg2 interaction.

## Discussion

Previous studies have described the significance of Atg23 in the formation of Atg9 vesicles, based on the observation that these vesicles decrease in the absence of this protein. In this study, we revealed that this decrease can also result from the non-productive fusion of Atg9 vesicles with as-yet-unidentified organelle membranes or vesicles. Accordingly, we propose a previously unrecognized post-vesicle formation role of Atg23 (Fig. 5I): Atg23 associated with Atg9 vesicles prevents their fusion with inappropriate membranes during their diffusion in the cytoplasm toward the PAS. The Atg9 S19D S948D mutation, which hampers this function of Atg23, tended to reduce Atg9 localization to the PAS, although the reduction was not statistically significant (Fig. EV3A,B), suggesting that the fusion-preventing function of Atg23 contributes to efficient delivery of Atg9 vesicles to the PAS. The fusion of Atg9 vesicles with inappropriate membranes was suggested by the following observations: the number of Atg9 vesicles decreased in the absence of Atg23 or the Atg9-Atg23 interaction was restored by inactivation or removal of Sec18 or the Atg9 vesicle-resident SNARE protein Gos1 (Sawa-Makarska et al, 2020). Although Gos1 is known to mediate intra-Golgi trafficking (McNew et al, 1998; Jahn and Scheller, 2006), abnormal Atg9 accumulation in the Golgi was not observed, even in cells lacking Atg23-mediated protection of Atg9 vesicles. Thus, the membranes with which Atg9 vesicles fuse in these cells remain elusive. The lack of detectable accumulation of Atg9 in specific organelles may be due to dilution of the Atg9 signal by fusion with multiple organelles and subsequent transport to other organelles.

In this study, we propose that Atg23 on Atg9 vesicles has a role in inhibiting their fusion with non-target membranes en route to the PAS. A possible mechanism for this inhibition is that Atg23 functions as a vesicle coat, similar to those seen in other vesicle transport pathways (Bonifacino and Glick, 2004). To explore this possibility from a structural perspective, we modeled an Atg9 vesicle as a sphere with a diameter of 60 nm and approximated it with a regular dodecahedron circumscribing the sphere (Fig. EV5J). Assuming that the vesicle contains 12 Atg9 trimers, given the experimental estimation that each Atg9 vesicle harbors ~30 Atg9 molecules (Yamamoto et al, 2012), and that each trimer forms an equilateral triangle with 12-nm sides, we positioned these triangles at the centers of the dodecahedron faces (Matoba et al, 2020; Maeda et al, 2020). Under this model, the distance between the centers of adjacent triangles is ~37 nm. Meanwhile, our analysis using truncated Atg23 mutants suggested that both the N- and C-terminal regions of Atg23 are involved in its interaction with Atg9 (Fig. EV5K,L). Given that these Atg23 regions are located at both ends of the Atg23 dimer and that the long axis of the dimer is ~32 nm (Hawkins et al, 2022) (Fig. EV5M), Atg23 dimers could bridge Atg9 trimers to cover a substantial portion of the vesicle surface, although they are unlikely to form a regular lattice. This Atg23 coating may serve as a physical obstacle to interactions between proteins that mediate vesicle fusion. It is also possible that the Atg23 coat interacts with factors involved in vesicle fusion on Atg9 vesicles, thereby masking their functions. Elucidating the architecture of Atg23-associated Atg9 vesicles would further advance our understanding of the mechanism by which Atg23 inhibits the fusion of Atg9 vesicles.

Our results also suggested that Atg23 dissociates from Atg9 vesicles upon Atg9 phosphorylation by Atg1 at the PAS, a process that can be regarded as vesicle uncoating, as observed for other transport vesicles (Lord et al, 2011; Bonifacino and Glick, 2004; Rothman and Schmid, 1986). Ser19 and Ser948 in Atg9, whose phosphorylation is involved in Atg23 dissociation, are both located within the Atg9 regions important for Atg23 binding (Legakis et al, 2007; Oeo-Santos et al, 2025; Bekkhozhin et al, 2026) (Fig. EV1E), suggesting that Atg1-mediated phosphorylation of these residues directly affects the Atg9-Atg23 interaction. Since Ser948 is located in proximity to Asp50 of Atg23, its phosphorylation is expected to cause electrostatic repulsion, which could contribute to destabilizing the interaction with Atg23. In cells defective in this Atg23 dissociation, such as those expressing phospho-deficient Atg9 mutants, the recruitment of Atg2, likely as the Atg2-Atg18 complex, was reduced, and autophagosome formation was compromised. We showed that the events downstream of Atg9 vesicle recruitment, i.e., PAS localization of PI3KCI, subsequent production of PI3P, and interaction between Atg9 and Atg2, are unchanged in these cells. Thus, the mechanism by which persistent retention of Atg23 at the PAS reduces Atg2 localization to the PAS remains unclear. In addition to the Atg2-Atg9 interaction, Atg18 binding to PI3P is known to play a pivotal role in the PAS localization of Atg2 (the Atg2-Atg18 complex) (Suzuki et al, 2007; Obara et al, 2008). Therefore, persistent association of Atg23 may limit Atg18 access to PI3P on Atg9 vesicles, thereby impairing recruitment of the Atg2–Atg18 complex to the PAS.

Atg23 dissociation from Atg9 vesicles may also facilitate downstream events during PAS organization, including the recruitment of factors other than Atg2. Previous studies suggested that several Atg9 vesicles assemble at the PAS, serving as a seed for the formation of a single autophagosome (Yamamoto et al, 2012). In addition, COPII vesicles are recruited to the PAS and eventually contribute to autophagosomal membranes (Shima et al, 2019). These findings suggest that Atg9 vesicles undergo fusion at the PAS in a homotypic and/or heterotypic manner. The release of Atg23 from Atg9 vesicles could also be a prerequisite for these fusion events at the PAS. The pronounced reduction in autophagic activity in the Atg9^S19A S948A^ mutant may reflect defects in these processes in addition to decreased recruitment of Atg2 (and other factors) to the PAS. These fusion events may also involve SNARE proteins on Atg9 vesicles that mediate their aberrant fusion in the absence of Atg23. To repurpose SNAREs that function in other membrane traffic pathways for autophagosome formation, cells would have needed a mechanism that blocks non-productive fusion of Atg9 vesicles during their cytoplasmic diffusion and releases this block upon their arrival at the PAS. To meet such requirements, cells may have acquired Atg9 vesicle coating and uncoating regulated through Atg9 phosphorylation by Atg1, which is specifically activated at the PAS.

In mammalian cells, ATG9 vesicles form via the AP-4 and AP-2 pathways in the Golgi and recycling endosomes, respectively (Imai et al, 2016; Mattera et al, 2017; Davies et al, 2018), and play similar roles in autophagosome biogenesis through interactions with ATG13 and ATG2 (Kannangara et al, 2021; van Vliet et al, 2022; Hama et al, 2023). In addition, the shuttling of ATG9 vesicles among the trans-Golgi network, endosomes, and the plasma membrane suggests the presence of SNARE proteins on the vesicles (Kakuta et al, 2017). Therefore, it is possible that, although mammalian cells lack a homolog of Atg23, other proteins on ATG9 vesicles play a similar role in protecting these vesicles from non-productive fusion during their trafficking to the autophagosome formation site.

## Methods

**Table:** Reagents and tools table

Reagent/resource	Reference or source	Identifier or catalog number
**Experimental models**		
*MATa ade2-1 ura3-1 his3-11,15 trp1-1 leu2-3,112 can1-100*	Thomas and Rothstein, 1989	W303-1A
W303-1A *ade2-1*::*ADE2 ATG9-GFP-hph*	This study	YKT12
YKT12 *atg23*Δ::*nat*	This study	YKT14
YKT12 *ATG23-6×FLAG-kan*	This study	YKT26
YKT26 *atg1*Δ::*zeo*	This study	YKT44
YKT26 *atg2*Δ::*zeo*	This study	YKT46
W303-1A *ade2-1*::*ADE2 ATG9-2×mCherry-hph ATG23-GFP-kan*	This study	YSMT528
YSMT528 *atg1*Δ::*zeo*	This study	YKT51
YSMT528 *atg2*Δ::*zeo*	This study	YKT52
W303-1A *ade2-1*::*ADE2 ATG9-GFP-hph atg11*Δ::*zeo atg17*Δ::Cg*TRP1*	This study	YKT53
W303-1A *ade2-1*::*ADE2 ATG23-GFP-hph atg9*Δ::*nat*	This study	YKT72
W303-1A *ade2-1*::*ADE2 atg9*Δ::*hph ATG23-6xFLAG-kan*	This study	YKT83
W303-1A *ade2-1*::*ADE2 atg17*Δ::*zeo lue2*::*ATG17-2xmCherry-305*	This study	ScTK1523-1
ScTK1523-1 *ATG9-GFP-hph*	This study	YKT90
YKT90 *atg1*Δ::*kan*	This study	YKT91
YKT90 *atg2*Δ::*kan*	This study	YKT92
ScTK1523-1 *ATG23-GFP-hph*	This study	YKT93
YKT93 *atg1*Δ::*kan*	This study	YKT94
YKT93 *atg2*Δ::*kan*	This study	YKT95
W303-1A *ade2-1*::*ADE2 atg11*Δ::*zeo atg17*Δ::*CgTRP1 ATG9-GFP-kan ATG23-FLAG-hph ura3*::*ATG1*^*WT*^*-GCN4*^*CC*^*-GNb-306 nat-ACT1pro-Z4EV-305*	This study	YKT103
W303-1A *ade2-1*::*ADE2 atg11*Δ::*zeo atg17*Δ::*CgTRP1 ATG9-GFP-kan ATG23-FLAG-hph ura3*::*atg1*^*D211A*^*-GCN4*^*CC*^*-GNb-306 nat-ACT1pro-Z4EV-305*	This study	YKT104
W303-1A *ade2-1*::*ADE2 PGK1-GFP-kan atg9*Δ::*nat*	This study	YKT109
YKT93 *atg2*Δ::*nat atg9*Δ::*kan*	This study	YKT110
YKT93 *atg5*Δ::*CgTRP1 atg9*Δ::*kan ATG2-mTagBFP2-nat*	This study	YKT113
W303-1A *ade2-1*::*ADE2 atg11*Δ::*zeo atg17*Δ::*CgTRP1 atg9*Δ::*kan ATG23-FLAG-hph ura3::ATG1*^*WT*^*-GCN4*^*CC*^*-GNb-306 nat-ACT1pro-Z4EV-305*	This study	YKT127
W303-1A *ade2-1*::*ADE2 atg5*Δ::*CgTRP1 ATG9-GNb*^*WT*^*-nat ATG23-GFP-hph ATG17-mCherry-zeo*	This study	YKT148
W303-1A *ade2-1*::*ADE2 atg5*Δ::*CgTRP1 ATG9-GNb*^*WT*^*-nat ATG23-GFP*^*nf*^*-hph ATG17-mCherry-zeo*	This study	YKT149
W303-1A *ade2-1*::*ADE2 atg5*Δ::*CgTRP1 ATG9-GNb*^*3A*^*-nat ATG23-GFP*^*nf*^*-hph ATG17-mCherry-zeo*	This study	YKT150
YKT149 *ATG2-mNeonGreen-kan*	This study	YKT151
YKT150 *ATG2-mNeonGreen-kan*	This study	YKT152
YKT149 *ATG14-mNeonGreen-kan*	This study	YKT153
YKT150 *ATG14-mNeonGreen-kan*	This study	YKT154
YKT149 *vps38*Δ::*CgHIS3 mNeonGreen-2×FYVE-306*	This study	YKT155
YKT150 *vps38*Δ::*CgHIS3 mNeonGreen-2×FYVE-306*	This study	YKT156
W303-1A *ade2-1*::*ADE2 atg9*Δ::*kan atg17*Δ::*CgTRP1 atg11*Δ::*zeo ura3::ATG1*^*WT*^*-GCN4*^*CC*^*-GNb-306 nat-ACT1pro-Z4EV-305*	This study	YKT161
W303-1A *ade2-1*::*ADE2 atg9*Δ::*kan atg17*Δ::*CgTRP1 atg11*Δ::*zeo ura3::atg1*^*D211A*^*-GCN4*^*CC*^*-GNb-306 leu2::pACT1-Z4EV-NAT-leu2d0-int*	This study	YKT162
YKT14 *gos1*Δ::*zeo*	This study	YKT163
YKT14 *sso1*Δ::*zeo*	This study	YKT164
W303-1A *ade2-1*::*ADE2 PGK1-mTagBFP2-kan*	This study	YKT189
YKT189 *atg2*Δ::*zeo*	This study	YKT190
YKT189 *ATG9-GFPnanobody*^*WT*^*-nat*	This study	YKT191
YKT189 *ATG9-GFPnanobody*^*WT*^*-nat ATG23-GFP-hph*	This study	YKT192
YKT189 *ATG9-GFPnanobody*^*3A*^*-nat ATG23-GFP-hph*	This study	YKT193
*MATα ura3-52 his3-200 leu2-3,112 trp1-901 lys2-801 suc2-9*	Darsow et al, 1997	SEY6120
SEY6120 *Z4EVpro-OsTIR1-306 nat-pACT1-Z4EV-305 sec18-aid-HA-hph ATG9-GFP-kan*	This study	YKT201
*YKT201 atg23*Δ::*zeo*	This study	YKT202
W303-1A *ade2-1*::*ADE leu2::pACT1-Z4EV-NAT-leu2d0-int atg2*Δ::*kan atg1*Δ::*zeo*	This study	ScTK2181
W303-1A *ade2-1*::*ADE2 ATG23-GFP-hph*	This study	YSMT647
W303-1A *ade2-1*::*ADE2 nat-CUP1pro-ATG23-GFP*	This study	YSMT648
W303-1A *ade2-1*::*ADE2 nat-CUP1pro-ATG23*Δ*N50-GFP*	This study	YSMT649
W303-1A *ade2-1*::*ADE2 ATG23*Δ*C54-GFP-hph*	This study	YSMT677
W303-1A *ade2-1*::*ADE2 ATG23*Δ*C104-GFP-hph*	This study	YSMT678
W303-1A *LEU2*::*mCherry-ATG8-hph ATG2-GFP-Kan*	This study	YSMT393
W303-1A *LEU2*::*mCherry-ATG8-hph ATG23-GFP-Kan*	This study	YSMT526
W303-1A *ATG9-GFP-3×FLAG-kan*	This study	YKT63
YKT63 *ATG23-GNb-zeo*	This study	YKT225
**Recombinant DNA**		
pRS316	Sikorski and Hieter, 1989	N/A
pRS316-ATG1	Matsuura et al, 1997	N/A
pRS316-ATG1 D211A	Matsuura et al, 1997	N/A
pRS316-ATG9	Yamamoto et al, 2012	N/A
pRS316-ATG9 S19A	This study	N/A
pRS316-ATG9 S948A	This study	N/A
pRS316-ATG9 S19A S948A	This study	N/A
pRS316-ATG9-GFP	Yamamoto et al, 2012	N/A
pRS316-ATG9-GFP S19A	This study	N/A
pRS316-ATG9-GFP S802A	This study	N/A
pRS316-ATG9-GFP S831A	This study	N/A
pRS316-ATG9-GFP S948A	This study	N/A
pRS316-ATG9-GFP S969A	This study	N/A
pRS316-ATG9-GFP S19A S948A	This study	N/A
pRS316-ATG9-GFP S19D	This study	N/A
pRS316-ATG9-GFP S802D	This study	N/A
pRS316-ATG9-GFP S831D	This study	N/A
pRS316-ATG9-GFP S948D	This study	N/A
pRS316-ATG9-GFP S969D	This study	N/A
pRS306-Z4EVpro-ATG1^WT^-Gcn4^CC^-GNb	This study	N/A
pRS306-Z4EVpro-ATG1^D211A^-Gcn4^CC^-GNb	This study	N/A
pRS306-Z4EVpro-ATG1^WT^-Gcn4^CC^-3xFLAG	This study	N/A
pRS304-ADHpro-mNeonGreen-2×FYVE	This study	N/A
pRS316-ATG9-GNb^WT^	This study	N/A
pRS316-ATG9-GNb^3A^	This study	N/A
pRS316-ATG9^S19D S948D^-GNb^WT^	This study	N/A
pRS316-ATG9^S19D S948D^-GNb^3A^	This study	N/A
pRS316-ADHp-GFP	This study	N/A
**Antibodies**		
mouse anti-GFP	Clontech	JL-8(632381)
mouse anti-mTagBFP2	Evrogen	Anti-tRFP antibody (AB233)
mouse anti-FLAG	Sigma-Aldrich	M2(F1804)
mouse anti-Pgk1	Invitrogen	22C5D8 - 459250
rat anti-HA	Roche	3F10
rabbit anti-mNeonGreen	Cell Signaling Technology	#53061
rabbit anti-Atg1	Matsuura et al, 1997	anti-Apg1
rabbit anti-Atg2	Shintani et al, 2001	anti-Apg2p
rabbit anti-Atg9	Noda et al, 2000	anti-Atg9 N-pep
rabbit anti-Atg23	Yamamoto et al, 2012	anti-Atg23
Goat HRP-conjugated secondary antibodies against Rabbit IgG	Jackson Immunoresearch	111-035-144
Goat HRP-conjugated secondary antibodies against Mouse IgG	Jackson Immunoresearch	315-035-003
Goat HRP-conjugated secondary antibodies against Rat IgG	Jackson Immunoresearch	112-035-003
**Chemicals, enzymes and other reagents**		
cOmplete EDTA-free protease inhibitor cocktail	Roche	Cat # 05056489001
PhosSTOP^TM^ phosphatase inhibitor tablets	Roche	Cat # 4906837001
Phenylmethylsulfonyl fluoride (PMSF)	Sigma-Aldrich	10837091001
*N*-Ethylmaleimide (NEM)	Nacalai tesque	15512-24
microcystin LR	Wako	136-12241
n-Dodecyl-β-D-maltopyranoside (DDM)	Nacalai tesque	14239-54
Triton-X100 (TX-100)	Nacalai tesque	35501-15
Rapamycin	LC Laboratories	R-5000
3-Indoleacetic Acid	Nacalai tesque	19119-61
KOD One PCR Master Mix	TOYOBO	KMM-101
PrimeSTAR Mutagenesis Basal Kit	TaKaRa	TKR-R045A
Gibson Assembly Cloning Kit	New England Biolabs	E5510S
bovine serum albumin	Sigma-Aldrich	A7030
NHS beads	TAMAGAWA SEIKI	TAS8848N1141
Used for YPD medium		
Bacto Yeast Extract	Becton, Dickinson and Company	212750
Bacto Peptone	Becton, Dickinson and Company	211677
D-(+)-Glucose	Nacalai tesque	16806-54
Used for SD + CA + ATU medium		
Difco Yeast Nitrogen Base w/o Amino Acids and Ammonium Sulfate	Becton, Dickinson and Company	233520
Ammonium Sulfate	Nacalai tesque	02620-04
Bacto Casamino acids	Becton, Dickinson and Company	223050
Sodium Hydroxide	Wako	198-13765
Adenine Sulfate	Wako	010-19612
L-Tryptophan	Wako	204-03382
Uracil	Wako	212-00062
**Software**		
ImageJ/Fiji	Schindelin et al, 2012	https://imagej.net/Fiji
AlphaFold Server	Abramson et al, 2024	https://alphafoldserver.com/
CueMol: Molecular Visualization Framework	developed by Ryuichiro Ishitani, Takanori Nakane	http://www.cuemol.org/ja/
MetaMorph software	Molecular Devices	
SoftWoRx version 7.0.0	GE Healthcare	
EvolutionCapt Version 18.0.12.0	Vilber	
GraphPad Prism 8.4.2	GraphPad Software	https://www.graphpad.com/features
**Other**		
Fluorescence microscope IX83	Olympus	IX83
DeltaVision Elite imaging system	GE Healthcare	DeltaVision Elite
FastPrep	MP Biomedicals	SKU:116004500
FUSION FX	Vilber	FX7.EDGE
Multi-beads shocker	Yasui Kikai	MB3200 (S)
0.5-mm zirconia beads	Yasui Kikai	YZB zirconia beads

### Yeast strains and media

Yeast strains used in this study are listed in Reagents and Tools Table. Knockout and tagging of chromosomal genes were performed based on a standard method using PCR-amplified DNA cassettes (Janke et al, 2004). Yeast strains were cultured at 30 °C in YPD medium [1% yeast extract, 2% peptone, and 2% glucose] for strain construction or in SD + CA + ATU medium [0.17% yeast nitrogen base without amino acids and ammonium sulfate, 0.5% ammonium sulfate, 0.5% casamino acid, 2% glucose, 3.75 mM NaOH, 0.002% adenine sulfate, 0.002% tryptophan, and 0.002% uracil] for immunoprecipitation, fluorescence microscopy and the Pgk1-GFP degradation assay. Yeast cells harboring pRS316-derived plasmids were cultured in SD + CA + ATU without uracil. To induce autophagy, cells were treated with 200 ng/ml rapamycin dissolved in 90% ethanol and 10% Tween 20. Expression of genes under the *Z4EV* promoter was induced by treatment with 1 μM β-estradiol dissolved in ethanol. Degradation of Sec18-AID-HA via the AID system was triggered by treatment with 0.5 μM indole-3-acetic acid (IAA) dissolved in ethanol.

### Plasmids

Plasmids used in this study are listed in Reagents and Tools Table. Plasmids based on pRS316 expressing Atg9, Atg9-GFP, and Atg9-FLAG were previously described (Yamamoto et al, 2012; Matoba et al, 2020), and point mutations were introduced using the KOD One PCR Master Mix or the PrimeSTAR Mutagenesis Basal Kit. Plasmids for expression of Atg23-HA in yeast were constructed as follows. The *ATG23* promoter (1,000 bp upstream of the start codon) and terminator (250 bp downstream of the stop codon) were amplified by PCR from genomic DNA of a yeast strain in which *ATG23* was tagged with HA. The resulting PCR product was cloned into pRS316 using the Gibson Assembly Kit.

### Fluorescence microscopy

Yeast cells were grown to the mid-log phase and treated with rapamycin for the indicated times. Fluorescence microscopy was performed at room temperature using an inverted fluorescence microscope (IX83; Olympus) equipped with 100× (UPLFLN U Plan Fluorite 100x Oil; Olympus) and 150× objective lens (UAPON 150XOTIRF; Olympus) for observation of the PAS and Atg9 vesicles, respectively. A 405-nm violet laser (50 mW; Coherent), a 488-nm blue laser (50 mW; Coherent), and a 588-nm yellow laser (50 mW; Coherent) were used for the excitation of mTagBFP2 and GFP or mNeonGreen and mCherry, respectively. Fluorescence was reflected by a dichroic mirror at 405-, 488-, and 588-nm wavelengths (Olympus), separated into two channels using the DV2 multichannel imaging system (Photometrics) equipped with a Di02-R594-25×36 dichroic mirror (Semrock), and then passed through a TRF59001-EM ET bandpass filter (Chroma) for the GFP/mNeonGreen channel and an FF01-624/40-25 bandpass filter (Semrock) for the mCherry channel. Images were acquired using an electron-multiplying CCD camera (ImagEM C9100-13; Hamamatsu Photonics K.K.) and MetaMorph software (Molecular Devices). Exposure time was 32 ms for observation of Atg9 vesicles and 500 ms for observation of the PAS. Obtained images were processed using Fiji (ImageJ) (Schneider et al, 2012). For PAS observations in Figs. 4G,  5A,E,G, and  EV5A,B,F cells were mounted on SD-N agarose gel pads [0.17% yeast nitrogen base without amino acids and ammonium sulfate, 2% glucose, and 3.5% (wt/vol) agarose] (Young et al, 2012; Hitomi et al, 2023).

Time-lapse fluorescence microscopy for analyzing the timing of Atg23 and Atg2 localization to the PAS (Fig. EV4A,B) was performed using a DeltaVision Elite imaging system (GE Healthcare) equipped with a sCMOS camera (pco.edge 5.5; PCO AG) and a 100× objective lens (UPlanSApo 100×/1.40 oil; Olympus, Tokyo, Japan). Five z-stack images at 0.2-μm intervals were acquired and deconvolved using SoftWoRx version 7.0.0 (GE Healthcare). Maximum intensity projections were generated using Fiji (ImageJ). Based on previous studies (Li et al, 2025), images were acquired at 45-s intervals. Atg2-GFP and Atg23-GFP were used to visualize Atg2 and Atg23, respectively, and mCherry-Atg8 was used as a marker for PAS formation. The appearance of an mCherry-Atg8 punctum was defined as time 0, and the timing of Atg2 and Atg23 recruitment was determined relative to this event.

### Quantification of Atg9 vesicle number

To quantify the number of Atg9 vesicles per focal plane, time-lapse imaging was performed on cells expressing Atg9-GFP or Atg9-mNeonGreen. Each time-lapse sequence consisted of 50 frames captured with an exposure time of 32 ms per frame. Image processing and analysis were performed using Fiji. As illustrated in Fig. EV2A, to extract mobile puncta (diffusing Atg9 vesicles), an average intensity projection image was generated from frames 1 to 50 of each series using the “Z Project” function (Projection type: Average Intensity). This average image, representing signals from immobile structures such as the Golgi and endosomes (immobile puncta), was subtracted from the first frame using the “Image Calculator” tool. The resulting difference image highlighted mobile puncta as positive signals, which were counted using the “Find Maxima” tool. The number of maxima detected was taken as the number of Atg9 vesicles.

### Immunoblot-based assessment of autophagic activity and protein expression levels

Yeast cells grown to the mid-log phase were treated with rapamycin, collected by centrifugation, and treated with 20% trichloroacetic acid (TCA) on ice for 15 min. After centrifugation at 17,700 × *g* for 5 min, the pellets were washed with ice-cold acetone, dried, and resuspended in SDS sample buffer [100 mM Tris-HCl (pH 7.5), 2% SDS, 10% glycerol, and a trace amount of bromophenol blue] containing 20 mM dithiothreitol (DTT) by mixing at 65 °C for 10 min. These suspensions were transferred to screw-capped tubes containing 0.5-mm YZB zirconia beads. The cells were then disrupted by a FastPrep. The resulting lysates were collected, boiled for 3 min, and clarified by centrifugation at 17,700 × *g* for 1 min. The supernatants were analyzed by immunoblotting. For immunoblotting, antibodies against GFP (JL-8; dilution 1:2000), mTagBFP2 (AB233; dilution 1:2000), mNeonGreen (#53061; dilution 1:2000), Atg9 (anti-Atg9 N-pep (Noda et al, 2000)), Pgk1 (#459250; dilution 1:10,000) and HA (3F10; dilution 1:2000) were used. Immunoblots were detected using FUSION FX with EvolutionCapt Version 18.0.12.0.

### ALP assay

ALP assay was performed as previously described (Noda et al, 1995). Briefly, yeast cells expressing Pho8Δ60 were grown to late log phase, then transferred to SD-N medium, and incubated for 4 h. These cells were resuspended in ALP buffer (1 M Tris-HCl (pH 9.0), 1 M MgSO₄, 10 mM ZnSO₄) and disrupted using glass beads to prepare cell lysates. Alkaline phosphatase activity was measured using an endpoint spectrophotometric assay based on the hydrolysis of p-nitrophenyl phosphate to p-nitrophenol. Absorbance of p-nitrophenol was measured at 405 nm, and the activity was normalized to total protein levels determined by the BCA assay. Data are presented as the mean ± standard deviation (SD) from three independent biological replicates.

### Immunoprecipitation

Anti-FLAG antibody [M2 (F1804)] and GFP-nanobody (GFP-binding protein) were conjugated to NHS FG magnetic beads according to the manufacturer’s protocol. In brief, NHS FG beads were treated with methanol and then incubated with anti-FLAG antibody or GFP-nanobody at room temperature for 30 min. The magnetic beads were mixed with 1 M 2-aminoethanol (pH 8.0) at 4 °C for 16–20 h to quench the conjugation reaction, washed three times with wash buffer [10 mM HEPES-NaOH (pH 7.9), 50 mM KCl, 1 mM EDTA, and 10% glycerol], and stored in wash buffer containing 1 mg/ml bovine serum albumin.

Yeast cells were disrupted in IP buffer [20 mM HEPES-KOH (pH 7.2), 0.8 M sorbitol, 5 mM EDTA, 2× complete protease inhibitor cocktail, and 1× PhosSTOP] using a Multi-beads shocker with 0.5-mm YZB zirconia beads. The lysates were solubilized with 0.2% Triton X-100 by rotating test tubes at 4 °C for 30 min and clarified by centrifugation at 2000 × *g* for 15 min followed by 15,000 × *g* for 5 min. The supernatants were incubated with antibody-conjugated magnetic beads at 4 °C for 1 h (Figs. 2A,C, EV1C,D, EV2E, and EV5I,K,L). The beads were washed three times with wash buffer [20 mM HEPES-KOH (pH 7.2), 0.8 M sorbitol, and 5 mM EDTA], and bound proteins were eluted by incubating the beads in SDS sample buffer at 65 °C for 10 min. These samples were collected, boiled for 5 min after addition of 20 mM DTT, clarified by centrifugation at 17,700 × *g* for 1 min, and analyzed by immunoblotting using antibodies against GFP, FLAG (M2; dilution 1:2000), Atg1 (anti-Apg1 (Matsuura et al, 1997); dilution 1:1000), Atg2 (anti-Apg2p (Shintani et al, 2001)) Atg9 (anti-Atg9 N-pep (Noda et al, 2000); dilution 1:1000), and Atg23 (23#1 (Yamamoto et al, 2012)).

In the experiments shown in Figs. 2G,H and  EV5H, spheroplasted yeast cells were incubated at 30 °C in 0.5× SD + CA + AT medium containing 1 M sorbitol and 200 ng/mL rapamycin for 2 h. These spheroplasts were resuspended in IP buffer containing 1% n-dodecyl-β-D-maltoside, solubilized at 4 °C for 30 min, and cleared by centrifugation at 2000 × *g* for 10 min, followed by 15,000 × *g* for 15 min, and the supernatants were subjected to immunoprecipitation as described above. These samples were analyzed by immunoblotting using antibodies against GFP and FLAG.

### In vitro phosphorylation of Atg9 vesicles by Atg1

Atg1^WT^-Gcn4^CC^-FLAG and Atg1^D211A^-Gcn4^CC^-FLAG used in the experiments shown in Fig. 2E,F were prepared as follows. Yeast cells harboring plasmids expressing either Atg1^WT^-Gcn4^CC^-FLAG or Atg1^D211A^-Gcn4^CC^-FLAG were cultured in SD + CA + ATU medium to mid-log phase, and their expression was induced by the addition of β-estradiol for 2 h. These cells were collected by centrifugation and disrupted in 400 μl of IP buffer using a Multi-beads shocker with 0.5-mm YZB zirconia beads. The lysates were solubilized by mixing with 400 μl of IP buffer containing 2% Triton X-100 and rotating at 4 °C for 30 min. The solubilized lysates were incubated with anti-FLAG antibody-conjugated NHS FG magnetic beads at 4 °C for 2 h. The beads were collected using a magnetic stand, washed once with IP buffer, and then washed twice with kinase reaction buffer [20 mM HEPES-KOH (pH 7.2), 50 mM NaCl, 5 mM magnesium acetate, 0.5 mM EDTA, and 0.8 M sorbitol]. Proteins were eluted by incubating the beads in 100 μl of kinase reaction buffer containing 1 mg/mL 3×FLAG peptide at 16 °C for 30 min.

Atg9 vesicle-bound beads were prepared as follows. Yeast cells expressing Atg9-GFP and Atg23-HA were cultured in SD + CA + ATU medium to mid-log phase, and treated with rapamycin for 2 h. These cells were disrupted by using zirconia beads, and the resulting lysates were incubated without solubilization with GNb-conjugated magnetic beads to capture Atg9 vesicles through Atg9-GFP, and the beads were washed twice with kinase reaction buffer.

The Atg9-vesicle-bound beads were mixed with either Atg1^WT^-Gcn4^CC^-FLAG or Atg1^D211A^-Gcn4^CC^-FLAG, and 5 mM ATP was added to initiate the kinase reaction. The mixture was incubated at 30 °C for 30 min. The beads were then collected using a magnetic stand, the supernatants were recovered (released fractions), and the beads were washed once with kinase reaction buffer. Proteins that remained bound to the beads were eluted by incubating the beads in 100 μl of SDS sample buffer at 65 °C for 10 min with shaking (bead-bound fractions). The released fractions were treated with 20% TCA on ice for 5 min, and after centrifugation at 17,700 × *g* for 5 min, the pellets were washed with ice-cold acetone, dried, and dissolved in SDS sample buffer. 20 mM DTT was added to both fractions, and the samples were boiled for 5 min and analyzed by immunoblotting using antibodies against FLAG, GFP, and HA.

### AlphaFold3 prediction

The structure of the *S. cerevisiae* Atg23 dimer alone and in complex with the N- and C-terminal fragments of Atg9 was predicted using AlphaFold3 (Abramson et al, 2024). The structural models shown in Figs. EV1E and EV5M were visualized using CueMol2.

### Quantification and statistical analysis

All experiments were independently repeated at least three times as biological replicates with similar results. No statistical methods were used to predetermine the sample size. Quantification of fluorescence microscopy and immunoblotting data was performed using Fiji software. For quantification of protein colocalization, the percentage of puncta of one protein that were positive for another was manually determined in three independent experiments, with at least 100 cells analyzed per experiment. The exact number of cells analyzed in each experiment is provided in the Source Data. Quantification of Atg9 vesicle number was performed by using Fiji as described in “Quantification of Atg9 vesicle number”, with the same replicates and cell numbers. Statistical tests were performed in GraphPad Prism. Data distribution was assumed to be normal, but this was not formally tested. Statistical significance was assessed using Tukey’s multiple comparisons test or unpaired two-tailed Student’s *t* test, as appropriate.

## Supplementary information


Peer Review File
Source data Fig. 1
Source data Fig. 2
Source data-1 Fig. 3
Source data-2 Fig. 3
Source data-3 Fig. 3
Source data Fig. 4
Source data Fig. 5
Figure EV1 Source Data
Figure EV2 Source Data-1
Figure EV2 Source Data-2
Figure EV3 Source Data
Figure EV4 Source Data
Figure EV5 Source Data
Expanded View Figures


## Data Availability

This study includes no data deposited in external repositories. All data supporting the findings of this study are available from the corresponding author upon reasonable request. The source data of this paper are collected in the following database record: biostudies:S-SCDT-10_1038-S44318-026-00867-0.

## References

[CR1] Aalto MK, Ronne H, Keränen S (1993) Yeast syntaxins Sso1p and Sso2p belong to a family of related membrane proteins that function in vesicular transport. EMBO J 12:4095–4104 8223426 10.1002/j.1460-2075.1993.tb06093.x PMC413702

[CR2] Abramson J, Adler J, Dunger J, Evans R, Green T, Pritzel A, Ronneberger O, Willmore L, Ballard AJ, Bambrick J et al (2024) Accurate structure prediction of biomolecular interactions with AlphaFold 3. Nature 630:493–500 38718835 10.1038/s41586-024-07487-w PMC11168924

[CR3] Araki Y, Ku WC, Akioka M, May AI, Hayashi Y, Arisaka F, Ishihama Y, Ohsumi Y (2013) Atg38 is required for autophagy-specific phosphatidylinositol 3-kinase complex integrity. J Cell Biol 203:299–313 24165940 10.1083/jcb.201304123 PMC3812978

[CR4] Backues SK, Orban DP, Bernard A, Singh K, Cao Y, Klionsky DJ (2015) Atg23 and Atg27 Act at the Early Stages of Atg9 Trafficking in *S. cerevisiae*. Traffic 16:172–190 25385507 10.1111/tra.12240 PMC4305007

[CR5] Baker D, Hicke L, Rexach M, Schleyer M, Schekman R (1988) Reconstitution of SEC gene product-dependent intercompartmental protein transport. Cell 54:335–344 3293799 10.1016/0092-8674(88)90196-1

[CR6] Bekkhozhin Z, Leary KA, Ragusa MJ (2026) Atg23 interacts with both the N- and C-termini of Atg9 via a hydrophobic binding pocket. J Biol Chem 302:113245. 10.1016/j.jbc.2026.113245 PMC13351150 42285518

[CR7] Block MR, Glick BS, Wilcox CA, Wieland FT, Rothman JE (1988) Purification of an N-ethylmaleimide-sensitive protein catalyzing vesicular transport. Proc Natl Acad Sci USA 85:7852–7856 3186695 10.1073/pnas.85.21.7852 PMC282295

[CR8] Bonifacino JS, Glick BS (2004) The mechanisms of vesicle budding and fusion. Cell 116:153–166 14744428 10.1016/s0092-8674(03)01079-1

[CR9] Cheong H, Nair U, Geng J, Klionsky DJ (2008) The Atg1 kinase complex is involved in the regulation of protein recruitment to initiate sequestering vesicle formation for nonspecific autophagy in *Saccharomyces cerevisiae*. Mol Biol Cell 19:668–681 18077553 10.1091/mbc.E07-08-0826 PMC2230592

[CR86] Darsow T, Rieder SE, Emr SD (1997) A multispecifi city syntaxin homologue, Vam3p, essential for autophagic and biosynthetic protein transport to the vacuole. J Cell Biol 138:517–529 10.1083/jcb.138.3.517 PMC2141632 9245783

[CR10] Davies AK, Itzhak DN, Edgar JR, Archuleta TL, Hirst J, Jackson LP, Robinson MS, Borner GHH (2018) AP-4 vesicles contribute to spatial control of autophagy via RUSC-dependent peripheral delivery of ATG9A. Nat Commun 9:3958 30262884 10.1038/s41467-018-06172-7 PMC6160451

[CR11] Dikic I, Elazar Z (2018) Mechanism and medical implications of mammalian autophagy. Nat Rev Mol Cell Biol 19:349–364 29618831 10.1038/s41580-018-0003-4

[CR12] Dokládal L, Stumpe M, Hu Z, Jaquenoud M, Dengjel J, De Virgilio C (2021) Phosphoproteomic responses of TORC1 target kinases reveal discrete and convergent mechanisms that orchestrate the quiescence program in yeast. Cell Rep 37:110149 34965436 10.1016/j.celrep.2021.110149

[CR13] Eakle KA, Bernstein M, Emr SD (1988) Characterization of a component of the yeast secretion machinery: identification of the SEC 18 gene product. Mol Cell Biol 8:4098–4109 3054509 10.1128/mcb.8.10.4098-4109.1988 PMC365479

[CR14] Epple UD, Suriapranata I, Eskelinen EL, Thumm M (2001) Aut5/Cvt17p, a putative lipase essential for disintegration of autophagic bodies inside the vacuole. J Bacteriol 183:5942–5955 11566994 10.1128/JB.183.20.5942-5955.2001 PMC99673

[CR15] Fujioka Y, Alam JM, Noshiro D, Mouri K, Ando T, Okada Y, May AI, Knorr RL, Suzuki K, Ohsumi Y et al (2020) Phase separation organizes the site of autophagosome formation. Nature 578:301–305 32025038 10.1038/s41586-020-1977-6

[CR16] Gómez-Sánchez R, Rose J, Guimarães R, Mari M, Papinski D, Rieter E, Geerts WJ, Hardenberg R, Kraft C, Ungermann C et al (2018) Atg9 establishes Atg2-dependent contact sites between the endoplasmic reticulum and phagophores. J Cell Biol 217:2743–2763 29848619 10.1083/jcb.201710116 PMC6080931

[CR17] Hama Y, Kurikawa Y, Matsui T, Mizushima N, Yamamoto H (2023) TAX1BP1 recruits ATG9 vesicles through SCAMP3 binding. Preprint at https://www.biorxiv.org/content/10.1101/2023.08.18.553817v1

[CR18] Hawkins WD, Leary KA, Andhare D, Popelka H, Klionsky DJ, Ragusa MJ (2022) Dimerization-dependent membrane tethering by Atg23 is essential for yeast autophagy. Cell Rep 39:110702 35443167 10.1016/j.celrep.2022.110702 PMC9097366

[CR19] Hitomi K, Kotani T, Noda NN, Kimura Y, Nakatogawa H (2023) The Atg1 complex, Atg9, and Vac8 recruit PI3K complex I to the pre-autophagosomal structure. J Cell Biol 222:e202210017 10.1083/jcb.202210017 PMC10337603 37436710

[CR20] Imai K, Hao F, Fujita N, Tsuji Y, Oe Y, Araki Y, Hamasaki M, Noda T, Yoshimori T (2016) Atg9A trafficking through the recycling endosomes is required for autophagosome formation. J Cell Sci 129:3781–3791 27587839 10.1242/jcs.196196

[CR21] Jahn R, Scheller RH (2006) SNAREs-engines for membrane fusion. Nat Rev Mol Cell Biol 7:631–643 16912714 10.1038/nrm2002

[CR22] Janke C, Magiera MM, Rathfelder N, Taxis C, Reber S, Maekawa H, Moreno-Borchart A, Doenges G, Schwob E, Schiebel E et al (2004) A versatile toolbox for PCR-based tagging of yeast genes: New fluorescent proteins, more markers and promoter substitution cassettes. Yeast 21:947–962 15334558 10.1002/yea.1142

[CR23] Kagohashi Y, Sasaki M, May AI, Kawamata T, Ohsumi Y (2023) The mechanism of Atg15-mediated membrane disruption in autophagy. J Cell Biol 222:e202306120 10.1083/jcb.202306120 PMC10622257 37917025

[CR24] Kakuta S, Yamaguchi J, Suzuki C, Sasaki M, Kazuno S, Uchiyama Y (2017) Small GTPase Rab1B is associated with ATG9A vesicles and regulates autophagosome formation. FASEB J 31:3757–3773 28522593 10.1096/fj.201601052R

[CR25] Kannangara AR, Poole DM, McEwan CM, Youngs JC, Weerasekara VK, Thornock AM, Lazaro MT, Balasooriya ER, Oh LM, Soderblom EJ et al (2021) BioID reveals an ATG9A interaction with ATG13-ATG101 in the degradation of p62/SQSTM1-ubiquitin clusters. EMBO Rep 22:EMBR202051136 10.15252/embr.202051136 PMC8490997 34369648

[CR26] Kawamata T, Kamada Y, Kabeya Y, Sekito T, Ohsumi Y (2008) Organization of the pre-autophagosomal structure responsible for autophagosome formation. Mol Biol Cell 19:2039–2050 18287526 10.1091/mbc.E07-10-1048 PMC2366851

[CR27] Kihara A, Noda T, Ishihara N, Ohsumi Y (2001) Two distinct Vps34 phosphatidylinositol 3-kinase complexes function in autophagy and carboxypeptidase y sorting in *Saccharomyces cerevisiae*. J Cell Biol 152:519–530 11157979 10.1083/jcb.152.3.519 PMC2196002

[CR28] Kotani T, Kirisako H, Koizumi M, Ohsumi Y, Nakatogawa H (2018) The Atg2-Atg18 complex tethers pre-autophagosomal membranes to the endoplasmic reticulum for autophagosome formation. Proc Natl Acad Sci USA 115:10363–10368 30254161 10.1073/pnas.1806727115 PMC6187169

[CR29] Landschulz WH, Johnson PF, Mcknight SL (1988) The leucine zipper: a hypothetical structure common to a new class of DNA binding proteins. Science 240:1759–1764 3289117 10.1126/science.3289117

[CR30] Lang T, Reiche S, Straub M, Bredschneider M, Thumm M (2000) Autophagy and the CVT pathway both depend on AUT9. J Bacteriol 182:2125–2133 10735854 10.1128/jb.182.8.2125-2133.2000 PMC111260

[CR31] Legakis JE, Yen WL, Klionsky DJ (2007) A cycling protein complex required for selective autophagy. Autophagy 3:422–432 17426440 10.4161/auto.4129

[CR32] Levine B, Kroemer G (2019) Biological functions of autophagy genes: a disease perspective. Cell 176:11–42 30633901 10.1016/j.cell.2018.09.048 PMC6347410

[CR33] Li H, Song JZ, He CW, Xie MX, Zhang ZT, Zhou Y, Li XJ, Cui L, Zhu J, Gong Q et al (2025) Temporal dissection of the roles of Atg4 and ESCRT in autophagosome formation in yeast. Cell Death Differ 32:866–879 39715823 10.1038/s41418-024-01438-8 PMC12089382

[CR34] Lord C, Bhandari D, Menon S, Ghassemian M, Nycz D, Hay J, Ghosh P, Ferro-Novick S (2011) Sequential interactions with Sec23 control the direction of vesicle traffic. Nature 473:181–186 21532587 10.1038/nature09969 PMC3093450

[CR35] Maeda S, Otomo C, Otomo T (2019) The autophagic membrane tether ATG2A transfers lipids between membranes. eLife 8:1–24 10.7554/eLife.45777 PMC6625793 31271352

[CR36] Maeda S, Yamamoto H, Kinch LN, Garza CM, Takahashi S, Otomo C, Grishin NV, Forli S, Mizushima N, Otomo T (2020) Structure, lipid scrambling activity and role in autophagosome formation of ATG9A. Nat Struct Mol Biol 27:1194–1201 33106659 10.1038/s41594-020-00520-2 PMC7718406

[CR37] Mari M, Griffith J, Rieter E, Krishnappa L, Klionsky DJ, Reggiori F (2010) An Atg9-containing compartment that functions in the early steps of autophagosome biogenesis. J Cell Biol 190:1005–1022 20855505 10.1083/jcb.200912089 PMC3101592

[CR38] Matoba K, Kotani T, Tsutsumi A, Tsuji T, Mori T, Noshiro D, Sugita Y, Nomura N, Iwata S, Ohsumi Y et al (2020) Atg9 is a lipid scramblase that mediates autophagosomal membrane expansion. Nat Struct Mol Biol 27:1185–1193 33106658 10.1038/s41594-020-00518-w

[CR39] Matsuura A, Tsukada M, Wada Y, Ohsumi Y (1997) Apg1p, a novel protein kinase required for the autophagic process in *Saccharomyces cerevisiae*. Gene 192:245–250 9224897 10.1016/s0378-1119(97)00084-x

[CR40] Mattera R, Park SY, De Pace R, Guardia CM, Bonifacino JS (2017) AP-4 mediates export of ATG9A from the trans-Golgi network to promote autophagosome formation. Proc Natl Acad Sci USA 114:E10697–E10706 29180427 10.1073/pnas.1717327114 PMC5740629

[CR41] Mayer A, Wickner W, Haas A (1996) Sec18p (NSF)-driven release of Sec17p (α-SNAP) can precede docking and fusion of yeast vacuoles. Cell 85:83–94 8620540 10.1016/s0092-8674(00)81084-3

[CR42] McIsaac RS, Oakes BL, Wang X, Dummit KA, Botstein D, Noyes MB (2013) Synthetic gene expression perturbation systems with rapid, tunable, single-gene specificity in yeast. Nucleic Acids Res 41:e57 23275543 10.1093/nar/gks1313 PMC3575806

[CR43] McNew JA, Coe JGS, Søgaard M, Zemelman BV, Wimmer C, Hong W, Söllner TH (1998) Gos1p, a *Saccharomyces cerevisiae* SNARE protein involved in Golgi transport. FEBS Lett 435:89–95 9755865 10.1016/s0014-5793(98)01044-8

[CR44] Meiling-Wesse K, Barth H, Thumm M (2002) Ccz1p/Aut11p/Cvt16p is essential for autophagy and the CVT pathway. FEBS Lett 526:71–76 12208507 10.1016/s0014-5793(02)03119-8

[CR45] Meiling-Wesse K, Bratsika F, Thumm M (2004) ATG23, a novel gene required for maturation of proaminopeptidase I, but not for autophagy. FEMS Yeast Res 4:459–465 14734026 10.1016/S1567-1356(03)00207-1

[CR46] Nakatogawa H (2020) Mechanisms governing autophagosome biogenesis. Nat Rev Mol Cell Biol 21:439–458 32372019 10.1038/s41580-020-0241-0

[CR47] Nakatogawa H, Suzuki K, Kamada Y, Ohsumi Y (2009) Dynamics and diversity in autophagy mechanisms: lessons from yeast. Nat Rev Mol Cell Biol 10:458–467 19491929 10.1038/nrm2708

[CR48] Nishimura K, Fukagawa T, Takisawa H, Kakimoto T, Kanemaki M (2009) An auxin-based degron system for the rapid depletion of proteins in nonplant cells. Nat Methods 6:917–922 19915560 10.1038/nmeth.1401

[CR49] Noda T, Kim J, Huang WP, Baba M, Tokunaga C, Ohsumi Y, Klionsky DJ (2000) Apg9p/Cvt7p is an integral membrane protein required for transport vesicle formation in the Cvt and autophagy pathways. J Cell Biol 148:465–480 10662773 10.1083/jcb.148.3.465 PMC2174799

[CR50] Noda T, Matsuura A, Wada Y, Ohsumi Y (1995) Novel system for monitoring autophagy in the yeast *Saccharomyces cerevisiae*. Biochem Biophys Res Commun 210:126–132 7741731 10.1006/bbrc.1995.1636

[CR51] Obara K, Sekito T, Niimi K, Ohsumi Y (2008) The Atg18-Atg2 complex is recruited to autophagic membranes via phosphatidylinositol 3-phosphate and exerts an essential function. J Biol Chem 283:23972–23980 18586673 10.1074/jbc.M803180200 PMC3259791

[CR52] Obara K, Sekito T, Ohsumi Y (2006) Assortment of phosphatidylinositol 3-kinase complexes—Atg14p directs association of complex I to the pre-autophagosomal structure in *Saccharomyces cerevisiae*. Mol Biol Cell 17:1527–1539 16421251 10.1091/mbc.E05-09-0841 PMC1415304

[CR53] Oeo-Santos C, Knüpling E, Davis C, Meng X, Maslen S, Kunzelmann S, D’Antuono R, Olerinyova A, Auchynnikava T, Skehel M et al (2025) A structural and regulatory framework for Atg9-containing vesicle formation and their Atg1-dependent remodelling during autophagy initiation. Preprint at https://www.biorxiv.org/content/10.1101/2025.11.06.685569v1.full

[CR54] Ohsumi Y (2014) Historical landmarks of autophagy research. Cell Res 24:9–23 24366340 10.1038/cr.2013.169 PMC3879711

[CR55] Osawa T, Kotani T, Kawaoka T, Hirata E, Suzuki K, Nakatogawa H, Ohsumi Y, Noda NN (2019) Atg2 mediates direct lipid transfer between membranes for autophagosome formation. Nat Struct Mol Biol 26:281–288 30911189 10.1038/s41594-019-0203-4

[CR56] Papinski D, Schuschnig M, Reiter W, Wilhelm L, Barnes CA, Maiolica A, Hansmann I, Pfaffenwimmer T, Kijanska M, Stoffel I et al (2014) Early steps in autophagy depend on direct phosphorylation of Atg9 by the Atg1 kinase. Mol Cell 53:471–483 24440502 10.1016/j.molcel.2013.12.011 PMC3978657

[CR57] Reggiori F, Tucker KA, Stromhaug PE, Klionsky DJ (2004) The Atg1-Atg13 complex regulates Atg9 and Atg23 retrieval transport from the pre-autophagosomal structure. Dev Cell 6:79–90 14723849 10.1016/s1534-5807(03)00402-7

[CR58] Rexach MF, Schekman RW (1991) Distinct biochemical requirements for the budding, targeting, and fusion of ER-derived transport vesicles. J Cell Biol 114:219–229 1649197 10.1083/jcb.114.2.219 PMC2289076

[CR59] Rieter E, Vinke F, Bakula D, Cebollero E, Ungermann C, Proikas-Cezanne T, Reggiori F (2013) Atg18 function in autophagy is regulated by specific sites within its β-propeller. J Cell Sci 126:593–604 23230146 10.1242/jcs.115725

[CR60] Rothbauer U, Zolghadr K, Tillib S, Nowak D, Schermelleh L, Gahl A, Backmann N, Conrath K, Muyldermans S, Cardoso MC et al (2006) Targeting and tracing antigens in live cells with fluorescent nanobodies. Nat Methods 3:887–889 17060912 10.1038/nmeth953

[CR61] Rothman JE, Schmid SL (1986) Enzymatic recycling of clathrin from coated vesicles. Cell 46:5–9 2872968 10.1016/0092-8674(86)90852-4

[CR62] Sawa-Makarska J, Baumann V, Coudevylle N, von Bülow S, Nogellova V, Abert C, Schuschnig M, Graef M, Hummer G, Martens S (2020) Reconstitution of autophagosome nucleation defines Atg9 vesicles as seeds for membrane formation. Science 369:eaaz7714 10.1126/science.aaz7714 PMC7610778 32883836

[CR63] Schneider CA, Rasband WS, Eliceiri KW (2012) NIH image to ImageJ: 25 years of image analysis. Nat Methods 9:671–675 22930834 10.1038/nmeth.2089 PMC5554542

[CR88] Schindelin J, Arganda-Carreras I, Frise E, Kaynig V, Longair M, Pietzsch T, Preibisch S, Rueden C, Saalfeld S, Schmid B,Tinevez JY, White DJ, Hartenstein V, Eliceiri K, Tomancak P, Cardona A (2012) Fiji: an open-source platform for biological-image analysis. Nat Methods 9:676–682 10.1038/nmeth.2019 PMC3855844 22743772

[CR64] Shima T, Kirisako H, Nakatogawa H (2019) COPII vesicles contribute to autophagosomal membranes. J Cell Biol 218:1503–1510 30787039 10.1083/jcb.201809032 PMC6504894

[CR65] Shintani T, Suzuki K, Kamada Y, Noda T, Ohsumi Y (2001) Apg2p functions in autophagosome formation on the perivacuolar structure. J Biol Chem 276:30452–30460 11382761 10.1074/jbc.M102346200

[CR66] Shirahama-Noda K, Kira S, Yoshimori T, Noda T (2013) TRAPPis responsible for vesicular transport from early endosomes to Golgi, facilitating Atg9 cycling in autophagy. J Cell Sci 126:4963–4973 23986483 10.1242/jcs.131318

[CR87] Sikorski RS, Hieter P (1989) A system of shuttle vectors and yeast host strains designed for efficient manipulation of DNA in Saccharomyces cerevisiae. Genetics 122:19–27 10.1093/genetics/122.1.19 PMC1203683 2659436

[CR67] Suzuki K, Kubota Y, Sekito T, Ohsumi Y (2007) Hierarchy of Atg proteins in pre-autophagosomal structure organization. Genes Cells 12:209–218 17295840 10.1111/j.1365-2443.2007.01050.x

[CR68] Suzuki SW, Yamamoto H, Oikawa Y, Kondo-Kakuta C, Kimura Y, Hirano H, Ohsumi Y (2015) Atg13 HORMA domain recruits Atg9 vesicles during autophagosome formation. Proc Natl Acad Sci USA 112:3350–3355 25737544 10.1073/pnas.1421092112 PMC4371973

[CR69] Takeshige K, Baba M, Tsuboi S, Noda T, Ohsumi Y (1992) Autophagy in yeast demonstrated with proteinase-deficient mutants and conditions for its induction. J Cell Biol 119:301–311 1400575 10.1083/jcb.119.2.301 PMC2289660

[CR70] Tanaka C, Tan LJ, Mochida K, Kirisako H, Koizumi M, Asai E, Sakoh-Nakatogawa M, Ohsumi Y, Nakatogawa H (2014) Hrr25 triggers selective autophagy-related pathways by phosphorylating receptor proteins. J Cell Biol 207:91–105 25287303 10.1083/jcb.201402128 PMC4195827

[CR71] Teter SA, Eggerton KP, Scott SV, Kim J, Fischer AM, Klionsky DJ (2001) Degradation of lipid vesicles in the yeast vacuole requires function of Cvt17, a putative lipase. J Biol Chem 276:2083–2087 11085977 10.1074/jbc.C000739200 PMC2749705

[CR85] Thomas BJ, Rothstein R (1989) Elevated recombination rates in transcriptionally active DNA. Cell 56:619–630 10.1016/0092-8674(89)90584-9 2645056

[CR72] Tucker KA, Reggiori F, Dunn WA, Klionsky DJ (2003) Atg23 is essential for the cytoplasm to vacuole targeting pathway and efficient autophagy but not pexophagy. J Biol Chem 278:48445–48452 14504273 10.1074/jbc.M309238200 PMC1705954

[CR73] Valverde DP, Yu S, Boggavarapu V, Kumar N, Lees JA, Walz T, Reinisch KM, Melia TJ (2019) ATG2 transports lipids to promote autophagosome biogenesis. J Cell Biol 218:1787–1798 30952800 10.1083/jcb.201811139 PMC6548141

[CR74] van Vliet AR, Chiduza GN, Maslen SL, Pye VE, Joshi D, De Tito S, Jefferies HBJ, Christodoulou E, Roustan C, Punch E et al (2022) ATG9A and ATG2A form a heteromeric complex essential for autophagosome formation. Mol Cell 82:4324–4339.e8 36347259 10.1016/j.molcel.2022.10.017

[CR75] Wang CW, Kim J, Huang WP, Abeliovich H, Stromhaug PE, Dunn WA, Klionsky DJ (2001) Apg2 is a novel protein required for the cytoplasm to vacuole targeting, autophagy, and pexophagy pathways. J Biol Chem 276:30442–30451 11382760 10.1074/jbc.M102342200 PMC2737745

[CR76] Watanabe Y, Iwasaki Y, Sasaki K, Motono C, Imai K, Suzuki K (2023) Atg15 is a vacuolar phospholipase that disintegrates organelle membranes. Cell Rep 42:113567 38118441 10.1016/j.celrep.2023.113567

[CR77] Welter E, Thumm M, Krick R (2010) Quantification of nonselective bulk autophagy in *S. cerevisiae* using Pgk1-GFP. Autophagy 6:794–797 20523132 10.4161/auto.6.6.12348

[CR78] Wilson DW, Wilcox CA, Flynn GC, Chen E, Kuang WJ, Henzel WJ, Block MR, Ullrich A, Rothman JE (1989) A fusion protein required for vesicle-mediated transport in both mammalian cells and yeast. Nature 339:355–359 2657434 10.1038/339355a0

[CR79] Yamamoto H, Kakuta S, Watanabe TM, Kitamura A, Sekito T, Kondo-Kakuta C, Ichikawa R, Kinjo M, Ohsumi Y (2012) Atg9 vesicles are an important membrane source during early steps of autophagosome formation. J Cell Biol 198:219–233 22826123 10.1083/jcb.201202061 PMC3410421

[CR80] Yamamoto H, Zhang S, Mizushima N (2023) Autophagy genes in biology and disease. Nat Rev Genet 24:382–400 36635405 10.1038/s41576-022-00562-w PMC9838376

[CR81] Yang Z, Klionsky DJ (2010) Eaten alive: a history of macroautophagy. Nat Cell Biol 12:814–822 20811353 10.1038/ncb0910-814 PMC3616322

[CR82] Yao W, Chen Y, Chen Y, Zhao P, Liu J, Zhang Y, Jiang Q, Wu C, Xie Y, Fan S et al (2023) TOR -mediated Ypt1 phosphorylation regulates autophagy initiation complex assembly. EMBO J 42:EMBJ2022112814 10.15252/embj.2022112814 PMC10548176 37635626

[CR83] Yeh YY, Shah KH, Herman PK (2011) An Atg13 protein-mediated self-association of the Atg1 protein kinase is important for the induction of autophagy. J Biol Chem 286:28931–28939 21712380 10.1074/jbc.M111.250324 PMC3190700

[CR84] Young JW, Locke JCW, Altinok A, Rosenfeld N, Bacarian T, Swain PS, Mjolsness E, Elowitz MB (2012) Measuring single-cell gene expression dynamics in bacteria using fluorescence time-lapse microscopy. Nat Protoc 7:80–88 10.1038/nprot.2011.432 PMC4161363 22179594

