# Supplementary material for: Atg23 prevents aberrant fusion of Atg9 vesicles during delivery to autophagosome formation sites
Source: EMBO J. 2026 Jul 20;45(17):6086–114. doi: 10.1038/s44318-026-00867-0 (PMC13534743; doi:10.1038/s44318-026-00867-0)
Supplement: Supplementary file 1 [file 44318_2026_867_MOESM15_ESM.pdf]

Expanded View Figures

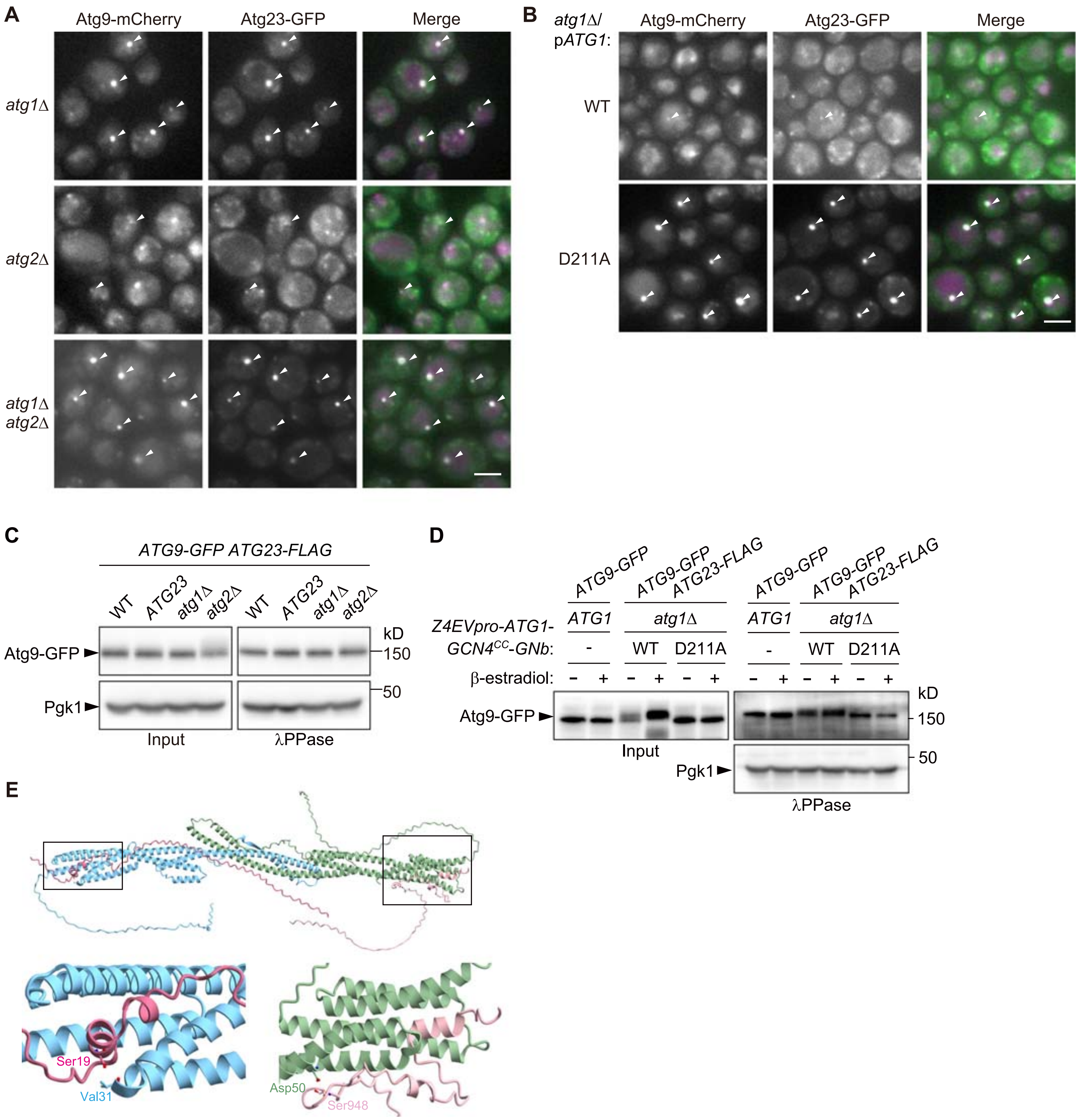

Figure EV1. PAS accumulation of Atg9 and Atg23 in cells lacking Atg1 kinase activity.

(**A**, **B**) Cells expressing Atg9-mCherry and Atg23-GFP were treated with rapamycin for 2 h and analyzed by fluorescence microscopy. Scale bars, 5 µm. White arrowheads, Atg23-GFP puncta colocalized with high-intensity Atg9-mCherry puncta (Atg9-mCherry localized to the PAS). (**C**) Cells expressing Atg9-GFP and Atg23-FLAG were treated with rapamycin for 2 h, and an aliquot of the cell lysates (Input) was treated with λ phosphatase for 30 min and analyzed by immunoblotting using antibodies against GFP and Pgk1. (**D**) Cells expressing Atg9-GFP and Atg23-FLAG were treated with β-estradiol for 2 h to induce expression of $Atg1^{WT}$-$Gcn4^{CC}$-GNb or $Atg1^{D211A}$-$Gcn4^{CC}$-GNb, and an aliquot of the cell lysates (Input) was treated with λ phosphatase for 30 min and analyzed by immunoblotting using antibodies against GFP and Pgk1. (**E**) AlphaFold 3-predicted structure of the Atg23 dimer in complex with Atg9 fragments (residues 1–50 and 897–997). These fragments are colored magenta and pink, respectively. Source data are available online for this figure.

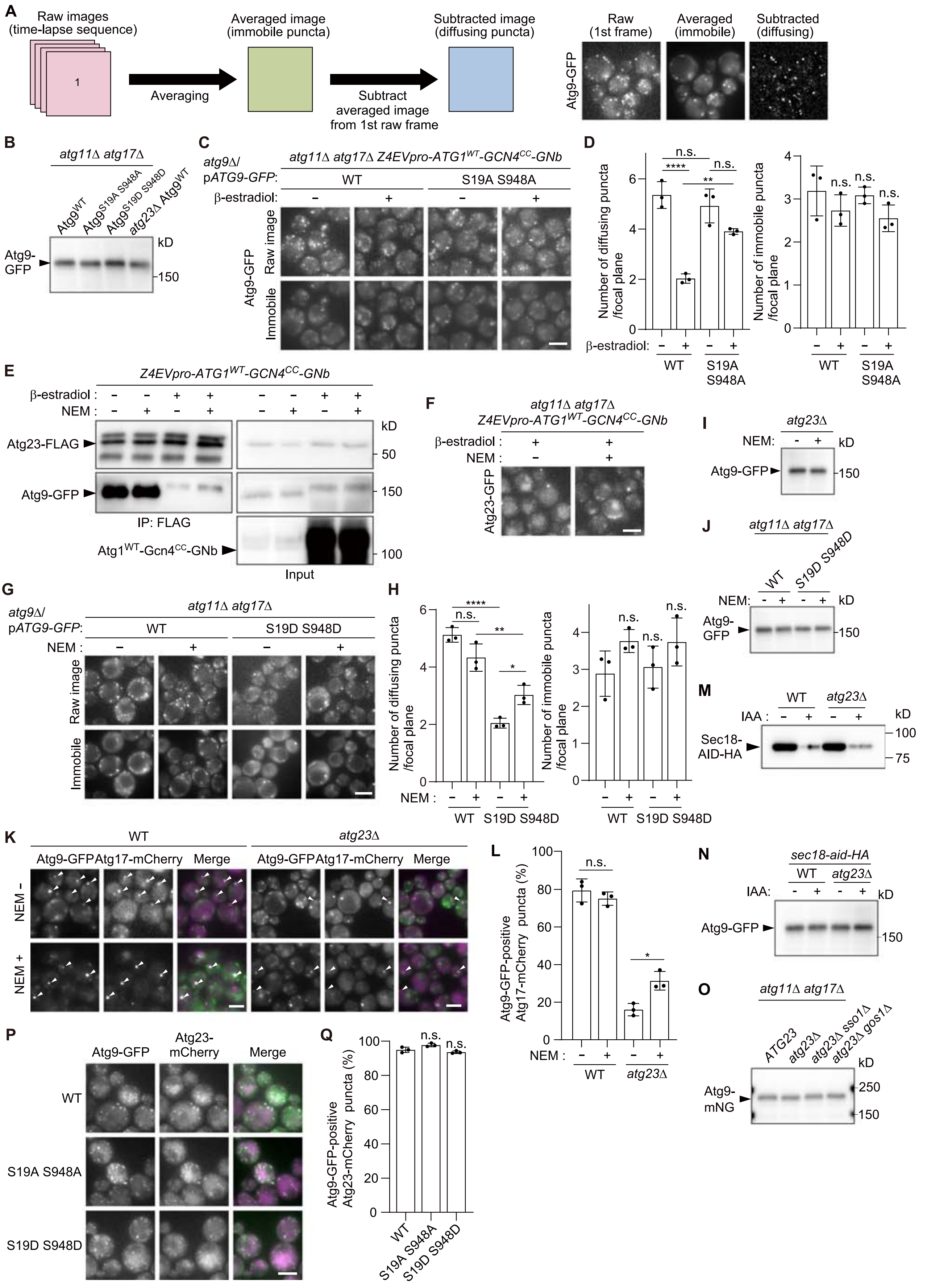
A
Raw images (time-lapse sequence)
1
Averaging
Averaged image (immobile puncta)
Subtract averaged image from 1st raw frame
Subtracted image (diffusing puncta)
Raw (1st frame)
Averaged (immobile)
Subtracted (diffusing)
Atg9-GFP
B
atg11Δ atg17Δ
Atg9WT
Atg9S19A S948A
Atg9S19D S948D
atg23Δ Atg9WT
kD
Atg9-GFP
150
C
atg9Δ/ pATG9-GFP:
atg11Δ atg17Δ Z4EVpro-ATG1WT-GCN4CC-GNb
WT
S19A S948A
β-estradiol:
Atg9-GFP
Raw image
Immobile
D
Number of diffusing puncta /focal plane
n.s.
****
n.s.
**
β-estradiol:
WT
S19A S948A
Number of immobile puncta /focal plane
n.s.
E
Z4EVpro-ATG1WT-GCN4CC-GNb
β-estradiol :
NEM :
Atg23-FLAG
Atg9-GFP
IP: FLAG
Atg1WT-Gcn4CC-GNb
Input
kD
50
150
100
F
atg11Δ atg17Δ Z4EVpro-ATG1WT-GCN4CC-GNb
β-estradiol :
NEM :
Atg23-GFP
I
atg23Δ
NEM:
kD
Atg9-GFP
150
J
atg11Δ atg17Δ
WT
S19D S948D
NEM:
kD
Atg9-GFP
150
G
atg9Δ/ pATG9-GFP:
atg11Δ atg17Δ
WT
S19D S948D
NEM :
Raw image
Immobile
H
Number of diffusing puncta /focal plane
****
n.s.
**
*
NEM :
WT
S19D S948D
Number of immobile puncta /focal plane
n.s.
M
WT
atg23Δ
IAA :
kD
Sec18-AID-HA
100
75
K
WT
atg23Δ
Atg9-GFP
Atg17-mCherry
Merge
NEM -
NEM +
L
Atg9-GFP-positive Atg17-mCherry puncta (%)
n.s.
*
NEM :
WT
atg23Δ
N
sec18-aid-HA
WT
atg23Δ
IAA:
kD
Atg9-GFP
150
O
atg11Δ atg17Δ
ATG23
atg23Δ
atg23Δ sso1Δ
atg23Δ gos1Δ
kD
Atg9-mNG
250
150
P
Atg9-GFP
Atg23-mCherry
Merge
WT
S19A S948A
S19D S948D
Q
Atg9-GFP-positive Atg23-mCherry puncta (%)
n.s.
WT
S19A S948A
S19D S948D

Figure EV2. Supporting analyses for Atg23-mediated inhibition of SNARE-dependent fusion of Atg9 vesicles.

(**A**) Image processing workflow for quantification of Atg9 vesicles. Time-lapse fluorescence microscopy was performed on cells expressing Atg9-GFP. To extract diffusing Atg9 puncta, 50 frames were stacked and averaged to generate an average intensity projection image representing immobile Atg9-GFP-positive structures such as the Golgi and endosomes. This averaged image was subtracted from the first frame, resulting in a difference image highlighting diffusing puncta. The resulting diffusing puncta image was used to quantify the number of Atg9 vesicles. Details are described in the "Methods" section. (**B**) Immunoblot analysis of Atg9-GFP expression levels in cells used for microscopy in Fig. 3C. The cell lysates were analyzed by immunoblotting using an antibody against GFP. (**C**) *atg11Δ atg17Δ* cells expressing Atg9-GFP were treated with β-estradiol for 2 h to induce expression of Atg1^{WT}-Gcn4^{CC}-GNb and analyzed by fluorescence microscopy as described in Fig. 3A. (**D**) Quantification of the number of diffusing puncta and immobile puncta per focal plane in the experiments shown in (**E**). Bars represent means ± SD ($n = 3$ independent biological replicates). $^{**}P < 0.01$, $^{****}P < 0.0001$, n.s., not significant (Tukey's multiple comparisons test). For number of Atg9 vesicles, WT β-estradiol- vs WT β-estradiol+ $P = 7.7 \times 10^{-5}$; WT β-estradiol- vs S19A S948A β-estradiol- $P = 6.5 \times 10^{-1}$; WT β-estradiol+ vs S19A S948A β-estradiol+ $P = 3.8 \times 10^{-3}$; S19A S948A β-estradiol- vs S19A S948A β-estradiol+ $P = 8.9 \times 10^{-2}$. For number of immobile puncta, WT β-estradiol- vs WT β-estradiol+ $P = 5.1 \times 10^{-1}$; WT β-estradiol- vs S19A S948A β-estradiol- $P = 9.9 \times 10^{-1}$; WT β-estradiol- vs S19A S948A β-estradiol+ $P = 2.6 \times 10^{-1}$. (**E**) Cells expressing Atg9-GFP and Atg23-FLAG were treated with β-estradiol to induce the expression of Atg1^{WT}-Gcn4^{CC}-GNb for 1.5 h and then with NEM for 30 min. The cell lysates (Input) were subjected to immunoprecipitation using anti-FLAG antibody, and the lysates and the immunoprecipitates (IP) were analyzed by immunoblotting using antibodies against FLAG, GFP, and Atg1. (**F**) Cells expressing Atg23-GFP were treated with β-estradiol and NEM as described in (**E**) and analyzed by fluorescence microscopy. Scale bar, 5 µm. (**G**) *atg11Δ atg17Δ* cells expressing Atg9-GFP were treated with NEM for 30 min and analyzed by fluorescence microscopy as described in Fig. 3A. Scale bar, 5 µm. (**H**) Quantification of the number of diffusing puncta and immobile puncta per focal plane in the experiments shown in (**G**). Bars represent means ± SD ($n = 3$ independent biological replicates). $^{**}P < 0.01$, $^{****}P < 0.0001$, n.s., not significant (Tukey's multiple comparisons test). For number of Atg9 vesicles, WT NEM- vs WT NEM + $P = 7.3 \times 10^{-2}$; WT NEM- vs S19D S948D NEM- $P = 1.5 \times 10^{-5}$; WT NEM+ vs S19D S948D NEM + $P = 5.7 \times 10^{-3}$; S19D S948D NEM- vs S19D S948D NEM + $P = 2.7 \times 10^{-2}$. For number of immobile puncta, WT NEM- vs WT NEM + $P = 2.8 \times 10^{-1}$; WT NEM- vs S19D S948D NEM- $P = 9.8 \times 10^{-1}$; WT NEM- vs S19D S948D NEM + $P = 3.0 \times 10^{-1}$. (**I**) Immunoblot analysis of Atg9-GFP expression levels in cells used for microscopy in Fig. 3G. The cell lysates were analyzed by immunoblotting using an antibody against GFP. (**J**) Immunoblot analysis of Atg9-GFP expression levels in cells used for microscopy in Fig. EV2G. The cell lysates were analyzed by immunoblotting using an antibody against GFP. (**K**) Cells expressing Atg9-GFP and Atg17-mCherry were treated with rapamycin for 1 h and then with NEM for 30 min and analyzed by fluorescence microscopy. Scale bars, 5 µm. White arrowheads, Atg17-mCherry puncta positive for Atg9-GFP. (**L**) Quantification of the percentage of Atg9-GFP-positive Atg17-mCherry puncta in the experiments shown in (**K**). Bars represent means ± SD ($n = 3$ independent biological replicates). $^{*}P < 0.05$, n.s., not significant (Tukey's multiple comparisons test): WT NEM- vs WT NEM + $P = 7.1 \times 10^{-1}$; *atg23Δ* NEM- vs *atg23Δ* NEM + $P = 3.4 \times 10^{-2}$. (**M**) Immunoblot analysis of Sec18-AID-HA expression levels in cells used for microscopy in Fig. 3I. Cells expressing Sec18-AID-HA were treated with β-estradiol for 15 min and then with IAA for 2 h, inducing OsTIR1 expression and Sec18-AID-HA degradation, respectively. The levels of Sec18-AID-HA were analyzed by immunoblotting using an antibody against HA. (**N**) Immunoblot analysis of Atg9-GFP expression levels in cells used for microscopy in Fig. 3I. The cell lysates were analyzed by immunoblotting using an antibody against GFP. (**O**) Immunoblot analysis of Atg9-mNeonGreen (Atg9-mNG) expression levels in cells used for microscopy in Fig. 3K. The cell lysates were analyzed by immunoblotting using an antibody against mNeonGreen. (**P**) Cells expressing Atg9-GFP and Atg23-mCherry were treated with rapamycin for 2 h and analyzed by fluorescence microscopy. Scale bar, 5 µm. (**Q**) Quantification of the percentage of Atg23-mCherry puncta positive for Atg9-GFP in the experiments shown in (**P**). Bars represent means ± SD ($n = 3$ independent biological replicates). n.s., not significant (Tukey's multiple comparisons test): Atg9^{WT} vs Atg9$^{S19A\ S948A}$ $P = 5.7 \times 10^{-2}$; Atg9^{WT} vs Atg9$^{S19D\ S948D}$ $P = 4.0 \times 10^{-1}$. Source data are available online for this figure.

© The Author(s)

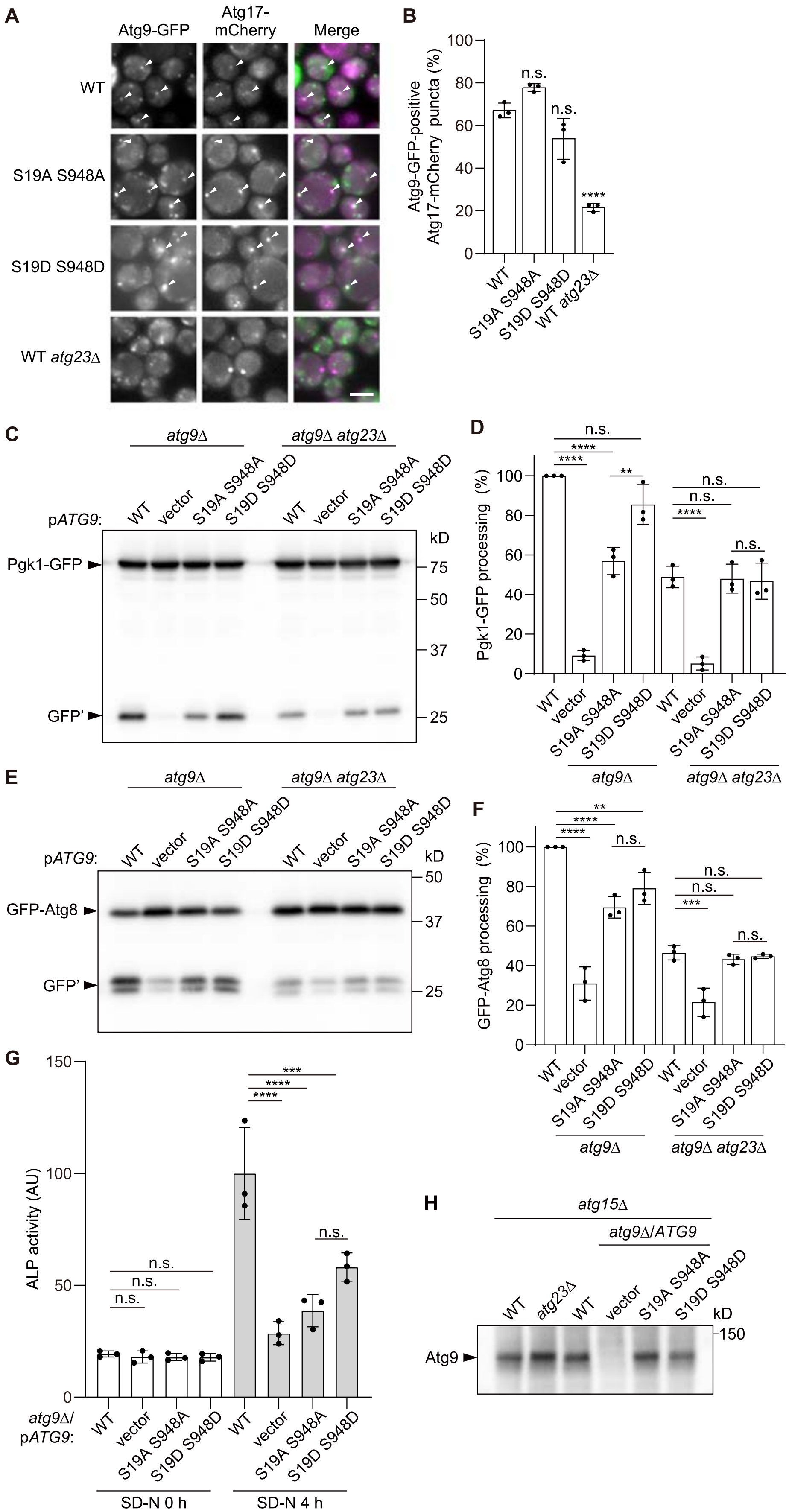
A
Atg9-GFP
Atg17-mCherry
Merge
WT
S19A S948A
S19D S948D
WT atg23Δ
B
Atg9-GFP-positive Atg17-mCherry puncta (%)
n.s.
n.s.
****
WT
S19A S948A
S19D S948D
WT atg23Δ
C
atg9Δ
atg9Δ atg23Δ
pATG9:
WT
vector
S19A S948A
S19D S948D
kD
Pgk1-GFP
GFP'
75
50
37
25
D
Pgk1-GFP processing (%)
n.s.
****
**
E
atg9Δ
atg9Δ atg23Δ
pATG9:
GFP-Atg8
GFP'
kD
50
37
25
F
GFP-Atg8 processing (%)
***
n.s.
G
ALP activity (AU)
atg9Δ/pATG9:
SD-N 0 h
SD-N 4 h
H
atg15Δ
atg9Δ/ATG9
WT
atg23Δ
vector
S19A S948A
S19D S948D
kD
150
Atg9

Figure EV3. Atg9 phosphorylation is required for efficient autophagy.

(**A**) Cells expressing Atg17-mCherry and either Atg9-GFP were treated with rapamycin for 2 h and analyzed by fluorescence microscopy. Scale bars, 5 µm. White arrowheads, Atg17-mCherry puncta positive for Atg9-GFP. (**B**) Quantification of the percentage of Atg17-mCherry puncta positive for Atg9-GFP in the experiments shown in (**A**). Bars represent means ± SD ($n = 3$ independent biological replicates). ****$P < 0.0001$, n.s., not significant (Tukey's multiple comparisons test): $Atg9^{WT}$ vs $Atg9^{S19A\ S948A}$ $P = 1.3 \times 10^{-1}$; $Atg9^{WT}$ vs $Atg9^{S19D\ S948D}$ $P = 5.8 \times 10^{-2}$; $Atg9^{WT}$ vs *atg23*Δ $P = 2.6 \times 10^{-5}$. (**C**) Cells expressing Pgk1-GFP were treated with rapamycin for 24 h and analyzed by immunoblotting using antibodies against GFP. (**D**) The band intensities of Pgk1-GFP and GFP fragments (GFP') in the experiments shown in (**C**) were measured, and the percentage of GFP fragments relative to the sum of Pgk1-GFP and GFP fragments was calculated as an index of Pgk1-GFP degradation. Bars represent means ± SD ($n = 3$ independent biological replicates) of values relative to WT set to 100. **$P < 0.01$, ****$P < 0.0001$ (Tukey's multiple comparisons test), n.s., not significant (Tukey's multiple comparisons test). In *atg9*Δ background, WT vs vector $P = 2.0 \times 10^{-10}$; WT vs S19A S948A $P = 9.2 \times 10^{-6}$; WT vs S19D S948D $P = 1.8 \times 10^{-1}$; S19A S948A vs S19D S948D $P = 1.1 \times 10^{-3}$. In *atg9*Δ *atg23*Δ background, WT vs vector $P = 7.6 \times 10^{-6}$; WT vs S19A S948A $P > 0.99$; WT vs S19D S948D $P > 0.99$; S19A S948A vs S19D S948D $P > 0.99$. (**E**) Cells expressing GFP-Atg8 were treated with rapamycin for 24 h and analyzed by immunoblotting using antibodies against GFP. (**F**) The band intensities of GFP-Atg8 and GFP fragments (GFP') in the experiments shown in (**E**) were measured, and the percentage of GFP fragments relative to the sum of GFP-Atg8 and GFP fragments was calculated as an index of GFP-Atg8 degradation. Bars represent means ± SD ($n = 3$ independent biological replicates) of values relative to WT set to 100. **$P < 0.01$, ***$P < 0.001$, ****$P < 0.0001$, n.s., not significant (Tukey's multiple comparisons test). In *atg9*Δ background, WT vs vector $P = 1.0 \times 10^{-9}$; WT vs S19A S948A $P = 8.0 \times 10^{-5}$; WT vs S19D S948D $P = 4.4 \times 10^{-3}$; S19A S948A vs S19D S948D $P = 4.2 \times 10^{-1}$. In *atg9*Δ *atg23*Δ background, WT vs vector $P = 7.9 \times 10^{-4}$; WT vs S19A S948A $P = 9.9 \times 10^{-1}$; WT vs S19D S948D $P > 0.99$; S19A S948A vs S19D S948D $P > 0.99$. (**G**) ALP activities in yeast cells expressing the Atg9 mutants. Cells expressing Pho8Δ60 and the Atg9 mutants were grown, and then incubated in SD-N medium for 4 h, and ALP activities in these cells were measured. Bars represent means ± SD ($n = 3$ independent biological replicates) of values relative to WT set to 100. **$P < 0.01$, ****$P < 0.0001$, n.s., not significant (Tukey's multiple comparisons test). In SD-N 0 h, WT vs vector $P > 0.99$; WT vs S19A S948A $P > 0.99$; WT vs S19D S948D $P > 0.99$. In SD-N 4 h, WT vs vector $P = 3.4 \times 10^{-7}$; WT vs S19A S948A $P = 2.7 \times 10^{-6}$; WT vs S19D S948D $P = 3.0 \times 10^{-4}$; S19A S948A vs S19D S948D $P = 1.5 \times 10^{-1}$. (**H**) Immunoblot analysis of Atg9 expression levels in cells used for microscopy in Fig. 4E. The cell lysates were analyzed by immunoblotting using an antibody. Source data are available online for this figure.

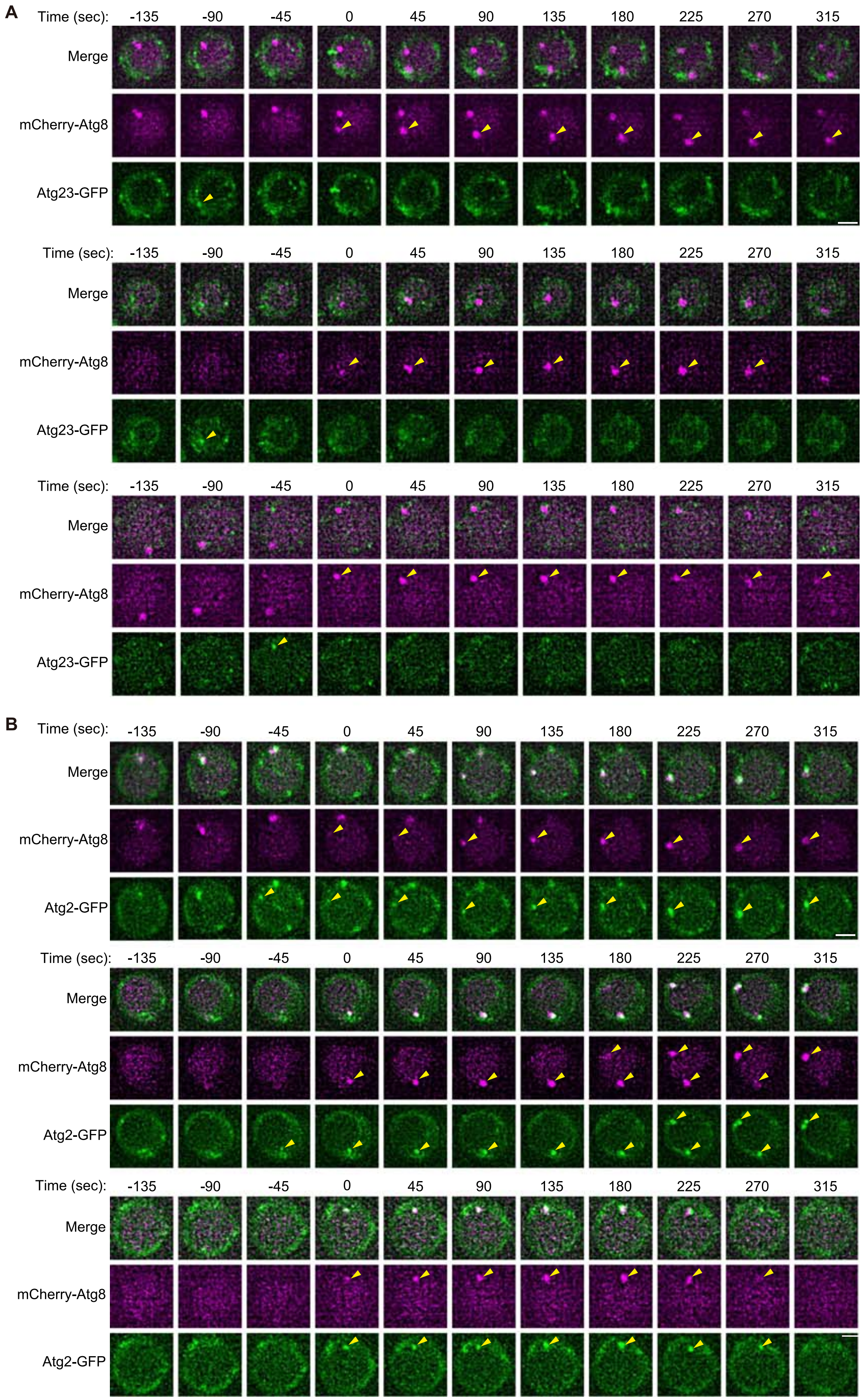
A
Time (sec): -135 -90 -45 0 45 90 135 180 225 270 315
Merge
mCherry-Atg8
Atg23-GFP
B
Atg2-GFP

 © The Author(s)

Figure EV4. Atg23 localizes to the PAS prior to Atg2 and dissociates earlier.

(**A**, **B**) Cells expressing mCherry-Atg8 and Atg23-GFP or Atg2-GFP were treated with rapamycin for 2 h and analyzed by fluorescence microscopy. Time-lapse images were acquired at 45-s intervals for 30 min. Scale bar, 2 µm. Yellow arrowheads indicate mCherry-Atg8, Atg23-GFP, or Atg2-GFP puncta. Source data are available online for this figure.

© The Author(s)

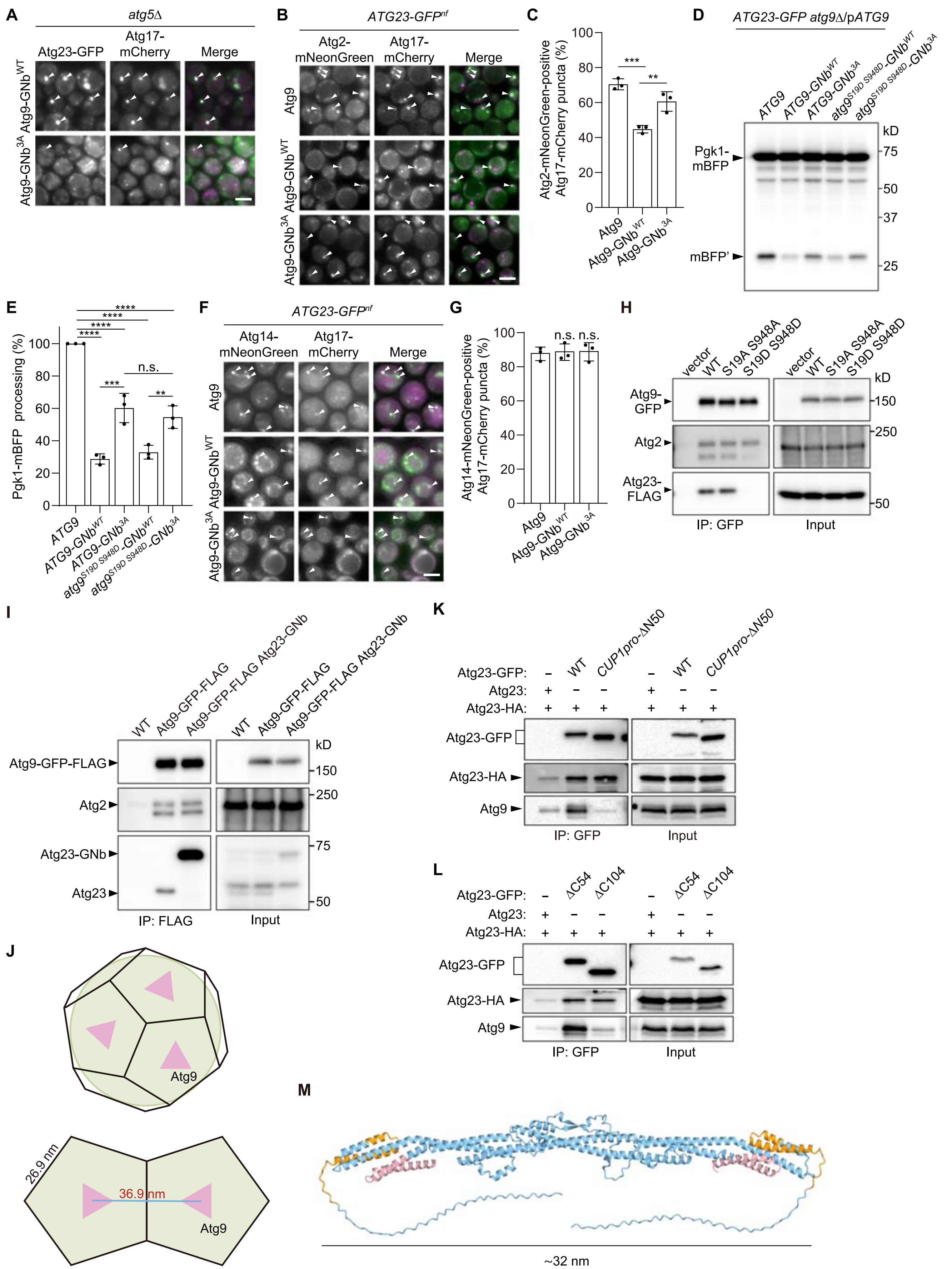
A
atg5Δ
Atg23-GFP
Atg17-mCherry
Merge
Atg9-GNbWT
Atg9-GNb3A
B
ATG23-GFPnf
Atg2-mNeonGreen
Atg17-mCherry
Merge
Atg9
Atg9-GNbWT
Atg9-GNb3A
C
Atg2-mNeonGreen-positive Atg17-mCherry puncta (%)
100
80
60
40
20
0
***
**
Atg9
Atg9-GNbWT
Atg9-GNb3A
D
ATG23-GFP atg9Δ/pATG9
ATG9
ATG9-GNbWT
ATG9-GNb3A
atg9S19D S948D-GNbWT
atg9S19D S948D-GNb3A
kD
Pgk1-mBFP
mBFP'
75
50
37
25
E
Pgk1-mBFP processing (%)
100
80
60
40
20
0
****
***
**
n.s.
ATG9
ATG9-GNbWT
ATG9-GNb3A
atg9S19D S948D-GNbWT
atg9S19D S948D-GNb3A
F
ATG23-GFPnf
Atg14-mNeonGreen
Atg17-mCherry
Merge
Atg9
Atg9-GNbWT
Atg9-GNb3A
G
Atg14-mNeonGreen-positive Atg17-mCherry puncta (%)
100
80
60
40
20
0
n.s.
n.s.
Atg9
Atg9-GNbWT
Atg9-GNb3A
H
vector
WT
S19A S948A
S19D S948D
kD
Atg9-GFP
Atg2
Atg23-FLAG
150
250
50
IP: GFP
Input
I
WT
Atg9-GFP-FLAG
Atg9-GFP-FLAG Atg23-GNb
kD
Atg9-GFP-FLAG
Atg2
Atg23-GNb
Atg23
150
250
75
50
IP: FLAG
Input
J
Atg9
26.9 nm
36.9 nm
K
Atg23-GFP:
Atg23:
Atg23-HA:
–
WT
CUP1pro-ΔN50
+
Atg23-GFP
Atg23-HA
Atg9
IP: GFP
Input
L
Atg23-GFP:
Atg23:
Atg23-HA:
–
ΔC54
ΔC104
+
Atg23-GFP
Atg23-HA
Atg9
IP: GFP
Input
M
~32 nm

 © The Author(s)

Figure EV5. Supporting analyses for Atg23-mediated inhibition of Atg2 recruitment.

(**A**) *atg5*Δ cells expressing Atg23-GFP, Atg17-mCherry, and either Atg9-GNb^{WT} or the 3 A mutant were treated with rapamycin for 2 h and analyzed by fluorescence microscopy. Scale bar, 5 µm. White arrowheads, Atg23-GFP-positive Atg17-mCherry puncta. (**B**) Cells expressing Atg2-mNeonGreen, Atg17-mCherry, Atg23-GFP^{nf}, and either non-tagged Atg9, Atg9-GNb^{WT}, or the 3 A mutant were treated with rapamycin for 2 h and analyzed by fluorescence microscopy. Scale bar, 5 µm. White arrowheads, Atg2-mNeonGreen-positive Atg17-mCherry puncta. (**C**) Quantification of the percentage of Atg2-mNeonGreen-positive Atg17-mCherry puncta in the experiments shown in (**B**). Bars represent means ± SD ($n = 3$ independent biological replicates). $^{**}P < 0.01$, $^{***}P < 0.001$; n.s., not significant (Tukey's multiple comparisons test): Atg9 vs Atg9-GNb^{WT} $P = 4.8 \times 10^{-4}$; Atg9 vs Atg9-GNb^{3A} $P = 4.8 \times 10^{-2}$. (**D**) Cells expressing Pgk1-mTagBFP2 were treated with rapamycin for 4 h and analyzed by immunoblotting using antibodies against mTagBFP2. (**E**) The band intensities of Pgk1-mTagBFP2 and mTagBFP2 fragments (mBFP') in the experiments shown in (**D**) were measured, and the percentage of mTagBFP2 fragments relative to the sum of Pgk1-mTagBFP2 and mTagBFP2 fragments was calculated as an index of Pgk1-mTagBFP2 degradation. Bars represent means ± SD ($n = 3$ independent biological replicates) of values relative to WT set to 100. $^{**}P < 0.01$, $^{***}P < 0.001$, $^{****}P < 0.0001$, n.s., not significant (Tukey's multiple comparisons test): $Atg9^{WT}$ vs $Atg9^{WT}$-GNb^{WT} $P = 2.0 \times 10^{-7}$; $Atg9^{WT}$ vs $Atg9^{WT}$-GNb^{3A} $P = 4.4 \times 10^{-5}$; $Atg9^{WT}$ vs $atg9^{S19D\ S948D}$-GNb^{WT} $P = 3.5 \times 10^{-7}$; $Atg9^{WT}$ vs $atg9^{S19D\ S948D}$-GNb^{3A} $P = 1.6 \times 10^{-3}$; $Atg9^{WT}$-GNb^{WT} vs $Atg9^{WT}$-GNb^{3A} $P = 3.3 \times 10^{-4}$; $Atg9^{WT}$-GNb^{3A} vs $atg9^{S19D\ S948D}$-GNb^{3A} $P = 7.4 \times 10^{-1}$; $Atg9^{S19D\ S948D}$-GNb^{WT} vs $Atg9^{S19D\ S948D}$-GNb^{3A} $P = 5.7 \times 10^{-3}$. (**F**) Cells expressing Atg14-mNeonGreen, Atg17-mCherry, Atg23-GFP^{nf}, and either non-tagged Atg9, Atg9-GNb^{WT}, or the 3A mutant were treated with rapamycin for 2 h and analyzed by fluorescence microscopy. Scale bar, 5 µm. White arrowheads, Atg14-mNeonGreen-positive Atg17-mCherry puncta. (**G**) Quantification of the percentage of Atg14-mNeonGreen-positive Atg17-mCherry puncta in the experiments shown in (**F**). Bars represent means ± SD ($n = 3$ independent biological replicates). n.s., not significant (Tukey's multiple comparisons test): Atg9 vs Atg9-GNb^{WT} $P = 9.6 \times 10^{-1}$; Atg9 vs Atg9-GNb^{3A} $P = 9.5 \times 10^{-1}$. (**H**) Cells expressing Atg9-GFP and Atg23-FLAG were treated with rapamycin for 2 h, and the cell lysates (Input) were subjected to immunoprecipitation using GNb-conjugated beads. The immunoprecipitates (IP) were analyzed by immunoblotting with antibodies against FLAG, GFP and Atg2. (**I**) Cells expressing Atg9-GFP and Atg23-FLAG were treated with rapamycin for 2 h, and the cell lysates (Input) were subjected to immunoprecipitation using anti-FLAG antibody-conjugated beads. The immunoprecipitates (IP) were analyzed by immunoblotting with antibodies against FLAG, GFP and Atg2. (**J**) Approximated Atg9 vesicle model for estimating the distance between Atg9 trimers on the vesicle surface (see Discussion for details). (**K**, **L**) Cells expressing Atg23-GFP and Atg23-HA were treated with rapamycin for 2 h. The cell lysates (Input) were subjected to immunoprecipitation using GNb-conjugated beads, and the immunoprecipitates (IP) were analyzed by immunoblotting using antibodies against GFP, HA, and Atg9. The Atg23 ΔN50 mutant was expressed under the *CUP1* promoter without $CuSO_4$ so that its protein level was comparable to that of the wild-type. The results showed that coimmunoprecipitation of Atg9 was markedly reduced in the ΔN50 (residues 1–50) and ΔC104 (residues 350–453) mutants but not in the ΔC54 (residues 400–453) mutant, suggesting that the N-terminal 50 residues and the C-terminal region encompassing residues 350–399 are involved in the interaction with Atg9. In contrast, coprecipitation of Atg23-HA was not affected by these deletions, suggesting that both mutations retain normal homodimerization ability. (**M**) The AlphaFold3-predicted structure of the Atg23 dimer. Atg23 residues 1–50 and 350–399, which are important for interaction with Atg9, are colored pink and orange, respectively. Source data are available online for this figure.

© The Author(s)
