# Supplementary material for: Atg23 prevents aberrant fusion of Atg9 vesicles during delivery to autophagosome formation sites
Source: EMBO J. 2026 Jul 20;45(17):6086–114. doi: 10.1038/s44318-026-00867-0 (PMC13534743; doi:10.1038/s44318-026-00867-0)
Supplement: Supplementary file 2 [file 44318_2026_867_MOESM1_ESM.pdf]

THE EMBO JOURNAL

Atg23 prevents aberrant fusion of Atg9 vesicles during delivery to autophagosome formation sites

Takumi Kimura, Takayuki Shima, Tetsuya Kotani, and Hitoshi Nakatogawa

Corresponding author(s): Hitoshi Nakatogawa (hnakatogawa@cbc.iir.isct.ac.jp)

Review Timeline:

Submission Date:	16th Oct 25
Editorial Decision:	14th Nov 25
Revision Received:	1st May 26
Editorial Decision:	12th Jun 26
Revision Received:	19th Jun 26
Accepted:	2nd Jul 26

Editor: William Teale

Transaction Report:

(Note: With the exception of the correction of typographical or spelling errors that could be a source of ambiguity, letters and reports are not edited. Depending on transfer agreements, referee reports obtained elsewhere may or may not be included in this compilation. Referee reports are anonymous unless the Referee chooses to sign their reports.)

Dear Prof. Nakatogawa,

Thank you again for the submission of your manuscript entitled "A regulated coating mechanism ensures Atg9 vesicle targeting for autophagosome biogenesis" and for your patience during the review process. We have now received reports from three referees, which I copy below.

As you can see from their comments, all thought that your manuscript could represent a useful and timely resource for the community. That said, all of them point out some issues that will require your attention before your manuscript can be published in The EMBO Journal.

Based on the overall interest expressed in the reports, however, I would like to invite you to address the comments of all referees in a revised version of the manuscript. I should add that it is The EMBO Journal policy to allow only a single major round of revision and that it is therefore important to resolve the main concerns at this stage. I believe the concerns of the referees are reasonable and addressable, but please contact me if you have any questions, need further input on the referee comments or if you anticipate any problems in addressing any of their points. Please, follow the instructions below when preparing your manuscript for resubmission. If you would like to dicuss these reports with me in person, I will be available at any time through the revision period over Zoom. Just let me know if you would like to talk over your plans for revision.

I would also like to point out that as a matter of policy, competing manuscripts published during this period will not be taken into consideration in our assessment of the novelty presented by your study ("scooping" protection). We have extended this 'scooping protection policy' beyond the usual 3 month revision timeline to cover the period required for a full revision to address the essential experimental issues. Please contact me if you see a paper with related content published elsewhere to discuss the appropriate course of action.

When preparing your letter of response to the referees' comments, please bear in mind that this will form part of the Review Process File, and will therefore be available online to the community. For more details on our Transparent Editorial Process, please visit our website: https://www.embopress.org/page/journal/14602075/authorguide#transparentprocess

Again, please contact me at any time during revision if you need any help or have further questions.

Thank you very much again for the opportunity to consider your work for publication. I look forward to your revision.

Best regards,

William

William Teale, Ph.D.
Editor
The EMBO Journal

When submitting your revised manuscript, please carefully review the instructions below and include the following items:

1) a .docx formatted version of the manuscript text (including legends for main figures, EV figures and tables). Please make sure that the changes are highlighted to be clearly visible.

2) individual production quality figure files as .eps, .tif, .jpg (one file per figure).

3) a .docx formatted letter INCLUDING the reviewers' reports and your detailed point-by-point response to their comments. As part of the EMBO Press transparent editorial process, the point-by-point response is part of the Review Process File (RPF), which will be published alongside your paper.

4) a complete author checklist, which you can download from our author guidelines (https://wol-prod-cdn.literatumonline.com/pb-assets/embo-site/Author Checklist%20-%20EMBO%20J-1561436015657.xlsx). Please insert information in the checklist that is also reflected in the manuscript. The completed author checklist will also be part of the RPF.

5) Please note that all corresponding authors are required to supply an ORCID ID for their name upon submission of a revised manuscript.

6) We require a 'Data Availability' section after the Materials and Methods. Before submitting your revision, primary datasets produced in this study need to be deposited in an appropriate public database, and the accession numbers and database listed

under 'Data Availability'. Please remember to provide a reviewer password if the datasets are not yet public (see https://www.embopress.org/page/journal/14602075/authorguide#datadeposition). If no data deposition in external databases is needed for this paper, please then state in this section: This study includes no data deposited in external repositories. Note that the Data Availability Section is restricted to new primary data that are part of this study.

Note - All links should resolve to a page where the data can be accessed.

7) When assembling figures, please refer to our figure preparation guideline in order to ensure proper formatting and readability in print as well as on screen:
http://bit.ly/EMBOPressFigurePreparationGuideline

Please remember: Digital image enhancement is acceptable practice, as long as it accurately represents the original data and conforms to community standards. If a figure has been subjected to significant electronic manipulation, this must be noted in the figure legend or in the 'Materials and Methods' section. The editors reserve the right to request original versions of figures and the original images that were used to assemble the figure.

8) For data quantification: please specify the name of the statistical test used to generate error bars and P values, the number (n) of independent experiments (specify technical or biological replicates) underlying each data point and the test used to calculate p-values in each figure legend. The figure legends should contain a basic description of n, P and the test applied. Graphs must include a description of the bars and the error bars (s.d., s.e.m.).

9) We would also encourage you to include the source data for figure panels that show essential data. Numerical data can be provided as individual .xls or .csv files (including a tab describing the data). For 'blots' or microscopy, uncropped images should be submitted (using a zip archive or a single pdf per main figure if multiple images need to be supplied for one panel). Additional information on source data and instruction on how to label the files are available at .

10) We replaced Supplementary Information with Expanded View (EV) Figures and Tables that are collapsible/expandable online (see examples in https://www.embopress.org/doi/10.15252/embj.201695874). A maximum of 5 EV Figures can be typeset. EV Figures should be cited as 'Figure EV1, Figure EV2" etc. in the text and their respective legends should be included in the main text after the legends of regular figures.

- For the figures that you do NOT wish to display as Expanded View figures, they should be bundled together with their legends in a single PDF file called *Appendix*, which should start with a short Table of Content. Appendix figures should be referred to in the main text as: "Appendix Figure S1, Appendix Figure S2" etc. See detailed instructions regarding expanded view here: .

- Additional Tables/Datasets should be labelled and referred to as Table EV1, Dataset EV1, etc. Legends have to be provided in a separate tab in case of .xls files. Alternatively, the legend can be supplied as a separate text file (README) and zipped together with the Table/Dataset file.

11) At EMBO Press we ask authors to provide source data for the main manuscript figures. Our source data coordinator will contact you to discuss which figure panels we would need source data for and will also provide you with helpful tips on how to upload and organize the files.

12) Our journal encourages inclusion of *data citations in the reference list* to directly cite datasets that were re-used and obtained from public databases. Data citations in the article text are distinct from normal bibliographical citations and should directly link to the database records from which the data can be accessed. In the main text, data citations are formatted as follows: "Data ref: Smith et al, 2001" or "Data ref: NCBI Sequence Read Archive PRJNA342805, 2017". In the Reference list, data citations must be labeled with "[DATASET]". A data reference must provide the database name, accession number/identifiers and a resolvable link to the landing page from which the data can be accessed at the end of the reference. Further instructions are available at .

13) In order to increase the reproducibility and reach of your work, The EMBO Journal includes a table of reagents that were used in the study. Please provide this along with your revisions.

Further instructions for preparing your revised manuscript:

Please make sure you upload a letter of response to the referees' comments together with the revised manuscript.

Please also check that the title and abstract of the manuscript are brief, yet explicit, even to non-specialists.

When assembling figures, please refer to our figure preparation guideline in order to ensure proper formatting and readability in print as well as on screen:

https://bit.ly/EMBOPressFigurePreparationGuideline
See also guidelines for figure legends: https://www.embopress.org/page/journal/14602075/authorguide#figureformat

At EMBO Press we ask authors to provide source data for the main manuscript figures. You will receive a separate email with instructions for providing source data with your revised manuscript, including how to upload and organize the files.

IMPORTANT: When you send the revision we will require
- a point-by-point response to the referees' comments, with a detailed description of the changes made (as a word file).
- a word file of the manuscript text.
- individual production quality figure files (one file per figure)
- a complete author checklist, which you can download from our author guidelines (https://www.embopress.org/page/journal/14602075/authorguide).
- Expanded View files (replacing Supplementary Information)
Please see out instructions to authors
https://www.embopress.org/page/journal/14602075/authorguide#expandedview
- a Reagents and Tools Table as part of the Methods section, which can be downloaded from our author guidelines (https://www.embopress.org/page/journal/14602075/authorguide#structuredmethods)

Please remember: Digital image enhancement is acceptable practice, as long as it accurately represents the original data and conforms to community standards. If a figure has been subjected to significant electronic manipulation, this must be noted in the figure legend or in the 'Materials and Methods' section. The editors reserve the right to request original versions of figures and the original images that were used to assemble the figure.

Further information is available in our Guide For Authors: https://www.embopress.org/page/journal/14602075/authorguide

We realize that it is difficult to revise to a specific deadline. In the interest of protecting the conceptual advance provided by the work, we recommend a revision within 3 months (12th Feb 2026). Please discuss the revision progress ahead of this time with the editor if you require more time to complete the revisions. Use the link below to submit your revision:

https://emboj.msubmit.net/cgi-bin/main.plex

Referee #1:

The authors explore the role of the autophagy protein Atg23, which binds to the conserved Atg9, with previously unclear functions in autophagy. The study uses a number of elegant genetic tools to discover that the interaction of Atg23 and Atg9 is regulated by the Atg1 kinase at the PAS. Atg23 binding to Atg9 prevents the SNARE-dependent decrease in Atg9 vesicles consistent with uncontrolled fusion of Atg9 vesicles with other compartments in the absence of Atg23. The authors identify two Atg1-dependent phosphorylation sites within Atg9, which regulate its interaction with Atg23. Based on their work, the authors propose that Atg23 coats Atg9 vesicle to prevent off-target fusion ensuring targeting to the PAS where Atg9 is phosphorylated by Atg1 to remove Atg23 and to allow for Atg9-Atg2 interaction.
Overall, the study provides compelling evidence for the negative regulatory role of Atg23 binding to Atg9 to prevent Atg9 vesicle fusion with off-target membranes and its regulation by Atg1 phosphorylation for removal of Atg23 at the PAS. There a few points that require clarification:

Critical points:
(1) What is the evidence that Atg23 functions as a vesicle coat protein? The authors provide compelling evidence that Atg23 binds to Atg9, inhibits SNARE-mediated fusion, and upon Atg1-dependent phosphorylation is released from Atg9 to allow for Atg2 recruitment. Canonical coat proteins have specific functions in vesicle formation, which have not been demonstrated for Atg23. Thus, the manuscript lacks the evidence that Atg23 functions as a structural 'coat' protein for the biogenesis of Atg9 vesicles. In fact, data in figure 3(M) suggest that Atg23 functions predominantly in preventing Atg9 vesicle fusion, because the effects on Atg9 biogenesis are relatively minor. It is fine to speculate or proposed the interesting model of an Atg23 coat in the discussion. However, as a title of the manuscript, it is somewhat speculative or overstated and, I think, should be changed. Currently, the data are consistent with the function of Atg23 as an inhibitor of Atg9 vesicle fusion and Atg2 interaction, which may not involve Atg23 function as a coat protein.

(2) The authors propose but do not provide evidence for the claim that Atg9 vesicles fuse with 'non-autophagic' membranes in the absence of Atg23 binding. It is equally possible that Atg9 vesicles homotypically fuse with each other (which could be considered autophagic membranes) to form bigger and potentially less mobile structures, which might be excluded from the current analyses. Such structures may be related to the Atg9 compartments observed upon Atg9 overexpression in Mari et al. 2010 JCB. The authors should at least discuss both possibilities or, better, provide experimental evidence for one or the other or

both. As commented on figure 3A/B below, in this context, it would be important to include the diffusing and immobile Atg9 structures in the analyses, especially, for the Atg9 mutants.

(3) A strength of the study is the use of a number of clever genetic tools to enable the analysis of intermediate steps in the process of PAS/autophagy protein assembly during autophagosome biogenesis. However, at the same time, this is also a potential weakness, because these genetic tools may or may not fully reflect the intermediates observed in an unperturbed process. Thus, it would be important to complement these approaches and more thoroughly assess the role/effects of Atg23 or Atg9 variants in an otherwise WT background with a direct readout for PAS assembly, autophagosome biogenesis, and autophagy flux.

(4) Key experiments that, I think, are missing: Do Atg9S19AS948A and/or Atg9S19DS948D affect Atg9-Atg23 colocalization in vivo? Experiments similar to figure 1A/B should be performed. Atg23 has been shown to bind to membranes (Hawkins et al. 2022, s. below), thus, Atg23 might still bind to Atg9SD vesicles. How do Atg9S19AS948A and/or Atg9S19DS948D affect Atg9 localization to Atg17-marked PAS? These experiments should also include an atg23 deletion background to test for epistasis relationships between Atg23 and Atg9 mutations.

Detailed comments:
The authors need to indicate how many cells were analyzed in each experiment.
Figure 1A-C should include the data for non-treated cells to confirm Atg9 and Atg23 colocalize in the absence of autophagy induction.
Figure 2A/B: input protein levels seem to reflect pulldown efficiencies. Authors need to quantify protein input levels with proper loading controls and, if needed, normalize pulldowns accordingly.
Figure 2G and H: It is counterintuitive that the authors treated cells with rapamycin prior to pulldown experiments. Wouldn't these experiments be more informative to test whether these changes in Atg9 are sufficient for affecting Atg23 binding. In the presence of rapamycin, Atg1 is activated and might phosphorylate additional sites aside from the mutated once. These experiments should be repeated without the addition of rapamycin.
Figure 2H: It might be worth testing whether deletion of Atg1 affects binding of Atg9 variants to Atg23 examining whether other kinases and/or sites are a factor.
Figure 3A/B: What is the logic of removing immobile Atg9 puncta here and not in figure 1? I think it would be helpful if the authors explained their rationale in the text (not just in material and methods). Additionally, the authors should quantify both, immobile and diffusing puncta in all key genotypes. This is particularly important for the atg9 mutant variants because they have not been previously analyzed in this way.
Line 217-220: "The results showed that atg9S19AS948A mutant cells are significantly defective in autophagosome formation". The data do not allow this conclusion, and it is an overinterpretation of the data. The authors can only conclude that the mutation reduces autophagy turnover. The reason could be autophagosome biogenesis, fusion, or degradation in the vacuole. However, the authors have not tested the different possibilities directly. Thus, the authors should measure the formation of autophagosomes in these strains to support their conclusion. Moreover, the atg9S19AS948A mutant displays reduced coimmunoprecipitation for Atg23 in figure 2H compared to Atg9, suggesting increased not decreased dissociation. Thus, these data are in conflict with the conclusion and proposed model. Is the atg9S19AS948A mutant only affected by reduced Atg23 binding or may other factors play a role? The authors should compare Pgk1-GFP turnover in Atg9 and atg9S19AS948A in atg23 deficient cells.
Figure 4C/D should include atg23 mutant background comparisons to test for epistasis.
Figure 4E: the authors need to mention the atg5 deficient strain background being used and explain the rationale in the text.
The authors should assess Atg9 and Atg2 interactions directly in dependence of Atg23 dissociation. For example, does Atg9 and Atg2 interact less in atg9S19AS948A and more in atg9S19DS948D mutant cells?
Figure 4I/J: the authors need to include a ATG9-GNb + GFP or protein-GFP control other than Atg23-GFP to control for unspecific steric inhibition. Additionally, it is important to test atg9S19DS948D -GNb + Atg23-GFP
The authors should discuss the recent publication directly relevant for their study: Hawkins et al. (2022), Cell Reports

Referee #2:

In this study, Takumi et al. elegantly demonstrated how Atg1 and Atg23 cooperate to ensure the proper targeting of Atg9 vesicles for autophagosome biogenesis. Through a series of well-designed and rigorous experiments, the authors addressed a long-standing question in the field of autophagy. Their results revealed that Atg23 suppresses SNARE-mediated fusion of Atg9 vesicles with non-autophagic membranes. Notably, they also uncovered a dynamic regulatory mechanism of autophagy: upon arrival of Atg9 vesicles at the phagophore assembly site, the kinase Atg1 phosphorylates the Atg9 region that interacts with Atg23, thereby promoting the recruitment of the downstream factor Atg2. Overall, the study is conceptually clear, methodologically sound, and supported by solid experimental data. It represents a fascinating and important contribution to the field. However, I have several questions that I would like the authors to clarify.

(1) In Figure 2C, phosphorylation of Atg9 by Atg1 appears to affect the protein level of Atg9, whereas the Atg1 kinase-dead mutant does not. Previous studies have reported that Atg1 indeed regulates the proteasomal degradation of Atg9, and the

authors should discuss this point in the Discussion section. Moreover, in Figure 2A, deletion of Atg2 also leads to a marked decrease in Atg9 protein levels. It is unclear whether this observation reflects a true biological effect. Could the loss of Atg2 influence Atg1 kinase activity, or might there be another underlying mechanism?

(2) In Figure 1E, the authors should clarify whether the phenotype of the Atg1 kinase-dead mutant resembles that of the Atg1 KO strain. This comparison is essential to confirm that the colocalization of Atg9 and Atg23 with the PAS is indeed regulated through the kinase activity of Atg1.

(3) In Figure 2G, the authors show that mimicking Atg1-mediated phosphorylation of Atg9 at S19 and S948 (S19D/S948D) significantly reduces the interaction between Atg9 and Atg23, clearly supporting the model that Atg1 phosphorylation promotes Atg9-Atg23 dissociation. However, it is puzzling that the non-phosphorylatable mutant (S19A-S948A) also shows a notable reduction in Atg23 binding. The authors should address this unexpected result in the Discussion. Could it be that the S19A-S948A mutation alter the structural conformation or stability of Atg9, thereby indirectly affecting its interaction with Atg23?

(4) In Figures 4C and 4D, given that the S19D-S948D mutant markedly reduces Atg9-Atg23 interaction and promotes Atg2 recruitment, one might expect enhanced autophagic activity. However, the autophagy activity of the S19D-S948D mutant appears even lower than that of the wild type. The authors should provide a possible explanation for this discrepancy.

Referee #3:

The manuscript by Kimura et al report a new role of Atg23 in regulating Atg9 vesicle trafficking. The authors noticed that in atg2D cells, although Atg9 accumulated at the PAS consistent with existing knowledge, Atg23 (an interactor of Atg9) did not display such accumulation. The authors hypothesized that Atg23 might dissociate from Atg9 vesicle resulting from weakened interaction, and found that Atg23 co-immunoprecipitated mildly more Atg9 in atg1D, and mildly less Atg9 in atg2D. In addition, Atg9 band displayed some upshift in atg2D, pointing a possibility of phosphorylation. Previous works reported Atg9 as a substrate of Atg1. The authors used a nanobody based system to tether Atg1 to Atg9, and found that forced tethering led to strong phosphorylation of Atg9 and loss of Atg23-Atg9 interaction in CoIP. They further confirmed that S-D substitution of two of the potential phosphorylation sites, S19 and S948, strongly reduced Atg23-Atg9 CoIP.
The authors then explored the potential functional consequence of the phosphorylation. Using the same Atg1 tethering tool, the authors found that forced tethering of Atg1 to Atg9 reduced the number of visible Atg9 vesicles. This reduction was reproduced by the SD mutant, and abolished by the SA mutant, consistent with a role of phosphorylation. Interestingly, inhibiting the function of NSF(Sec18) led to moderate recovery of Atg9 vesicle number in the presence of Atg1 tethering, and in atg23D cells. Knocking out a SNARE protein also partially recovered Atg9 vesicle number. The authors interpreted the data as Atg23 prevents unintended fusion of Atg9 vesicle to non-PAS membrane.
Another function the authors proposed is that the release of Atg23 promotes recruitment of Atg2 to move forward in autophagosome biogenesis. The authors observed in atg2D cells that SA substitutions in Atg9 led to a mild increase in the presence of Atg23 at PAS (marked by Atg17). In atg5D cells, they found a mild decrease of Atg2 dots at PAS with the SA allele. Forced tethering of Atg23 to Atg9 (via the nanobody technique) led to a clear reduction of Atg2 dots at PAS, but did not affect the PI3K complex. The authors also observed a major reduction of autophagic substrate degradation (Pgk1) in SA mutant cells, and a mild reduction in SD mutant cells.
Overall, this is a very interesting story. How various Atg proteins move step by step to participate in autophagosome biogenesis is a challenging and poorly explored aspect in mechanistic studies of autophagy. Here, the authors' work has the potential to make a key contribution. However, the current manuscript contains several weakness that need to be addressed, and feels preliminary in its current form.

Major issues:
1.
My biggest confusion is on how important the reported phosphorylation is in the big picture. The authors demonstrated nicely that phosphorylation of Atg9 by Atg2 can strongly inhibit Atg23-Atg9 interaction. This is a very strong effect. It appears that SA allele of Atg9 interacts constitutively with Atg23, and SD allele of Atg9 essentially loses the interaction. The authors also reported a large reduction of Atg9 vesicle number in SD mutant, and a mild reduction of Atg2 dots in SA mutant. On the other hand, it was the SA allele that produced a major reduction in the degradation of Pgk1, with the SD having only a mild reduction. In other words, from the end result, the authors are essentially saying that loss of Atg23 interaction (SD allele, strong reduction of Atg9 vesicle number) is not important for autophagic flux, whereas constitutive interaction is strongly inhibitive (but without an explanation that match the scale).
Using a single substrate (Pgk1) to report autophagic flux has the potential to generate bias. I suggest that the authors conduct the more quantitative pho8D60 assay, and the more commonly used GFP-Atg8 processing assay, to allow better interpretation of the resulting autophagic flux and cross comparison with other yeast autophagy work. The significance of excess Atg23-Atg9

interaction and loss of interaction may appear different with these assays.
Along this line, as Atg9 was reported to affect autophagosome number instead of size, I suggest that the authors perform TEM to dissect whether reduced autophagic flux resulted from reduced autophagosome number in the context of disturbed Atg23-Atg9 interaction.
To facilitate cross comparison, atg23D should also be included as a control in these assays.
2.
Assuming that additional assays of autophagic flux suggested above end up reporting results consistent with the Pgk1 result, then I have the question of why a strong reduction of Atg9 vesicle number did not lead to a matching reduction in autophagosome number with the SD allele or Atg1 tethering.
The authors should examine the presence of Atg9 vesicle at PAS (the existing data are about non-PAS vesicles) to see if such a level of reduction in total number is actually tolerable for the cells to maintain a close to normal presence of Atg9 at the PAS, which might explain such discrepancy.
Note that the authors tend to use various atg knockouts to arrest autophagosome biogenesis (see also latter discussion). It is critical that a comparison under close-to-WT genotype should be included, beside any atg knockouts that may facilitate the observation.
3.
The authors propose that phosphorylation of Atg9 allows the release of Atg23, and in turn recruitment of Atg2. Based on a recent study (PMID: 39715823), there is only a very short time window of about 1 min from the formation of Atg17 dots to the formation of Atg2 dots. The authors' model imply that it is this short period that Atg23 reside at the PAS. To provide direct support for the authors' hypothesis, I suggest that the authors observe by time-lapse imaging to pinpoint when Atg9 and Atg23 arrive and leave the PAS relative to other key Atg proteins, in particular Atg2.

Minor issues:
1.
The coat hypothesis is very interesting, but I have some reservation. The authors should at least provide quantitative data on the number of Atg23 molecules present at each Atg9 vesicle and see how it compares with the number of Atg9 molecules. There are other possible mechanisms that can explain the current observation. Examining and ruling out the alternatives is outside the scope of this work. But I think the authors need stronger evidence, such as cryoEM, to make such a claim.
2.
Fig. 3A-N, the authors described a method of removing immobile Atg9 dots in text. But it is not immediately evident what images are displayed here. Are these raw images, or computed images only showing the mobile dots? Also, given the reported number, I guess the authors were quantifying dots per slice, not the total number of Atg9 dots per cell that would require the capturing of z-stacks. In either case, more explicit statements are needed.
3.
In Fig. 4, the authors used atg2D and atg5D cells when observing Atg23 and Atg2 localization at PAS. The effect of Atg9 phosphorylation on their presence should also be examined in a close-to-WT genotype to rule out the possibility that this regulation is only important under conditions that arrest autophagosome biogenesis, and much less important under normal conditions (as when measuring autophagic flux).
The use of these mutants should be also be clearly stated in the main text when reporting the results (starting from line 205).
4.
Please add immunoblots of Atg9 along fluorescent images in Fig.3 to rule out the possibility that the reduction of Atg9 vesicle number is caused by protein instability.
5.
In Fig. EV1A, it appears that the accumulation of Atg9 was much stronger in atg1D cells than in atg2D cells. This matches our general impression when working with them. However, in Fig. 1D, the signal of Atg9 in atg1D cells appeared quite dark instead. The cytosolic background also appeared weak in this image. Did the authors use auto-contrast when generating the image in Fig. 1D? Auto-contrasting function of software will make images with bright objects appear this way.
Fig. EV1B may also have this problem. If the authors used any form of auto-contrast/brightness, please go back to raw data and manually set a fixed display threshold, and check other images in the manuscript.
6.
Please quantify the total amounts of Atg9 in Fig. 2A. It appears that the amount was lower in atg2D.
7. Line 41-44, the description of the six functional groups is somewhat unconventional here. The Atg16 complex is rarely used as the name for the Atg12 conjugation system. Also, in light of the many functions of Atg1/ULK1 at late stages of autophagosome formation, and its localization pattern that covers autophagosomes instead of focusing on a dot, the continued use of "Atg1 complex" is potentially misleading.
8.
Given the mild effect of Atg9 SA mutants on Atg2 recruitment, and the strong effect in autophagic flux, I suspect that there are other more important processes yet to be discovered. This mismatch should be discussed.
9.
Can the authors explain why the AID tool OsTIR1 is expressed by the Z4EV promoter (line 188)?

Response to Reviewers' Comments

Referee #1:

The authors explore the role of the autophagy protein Atg23, which binds to the conserved Atg9, with previously unclear functions in autophagy. The study uses a number of elegant genetic tools to discover that the interaction of Atg23 and Atg9 is regulated by the Atg1 kinase at the PAS. Atg23 binding to Atg9 prevents the SNARE-dependent decrease in Atg9 vesicles consistent with uncontrolled fusion of Atg9 vesicles with other compartments in the absence of Atg23. The authors identify two Atg1-dependent phosphorylation sites within Atg9, which regulate its interaction with Atg23. Based on their work, the authors propose that Atg23 coats Atg9 vesicle to prevent off-target fusion ensuring targeting to the PAS where Atg9 is phosphorylated by Atg1 to remove Atg23 and to allow for Atg9-Atg2 interaction.
Overall, the study provides compelling evidence for the negative regulatory role of Atg23 binding to Atg9 to prevent Atg9 vesicle fusion with off-target membranes and its regulation by Atg1 phosphorylation for removal of Atg23 at the PAS. There a few points that require clarification:

Critical points:

(1) What is the evidence that Atg23 functions as a vesicle coat protein? The authors provide compelling evidence that Atg23 binds to Atg9, inhibits SNARE-mediated fusion, and upon Atg1-dependent phosphorylation is released from Atg9 to allow for Atg2 recruitment. Canonical coat proteins have specific functions in vesicle formation, which have not been demonstrated for Atg23. Thus, the manuscript lacks the evidence that Atg23 functions as a structural 'coat' protein for the biogenesis of Atg9 vesicles. In fact, data in figure 3(M) suggest that Atg23 functions predominantly in preventing Atg9 vesicle fusion, because the effects on Atg9 biogenesis are

relatively minor. It is fine to speculate or proposed the interesting model of an Atg23 coat in the discussion. However, as a title of the manuscript, it is somewhat speculative or overstated and, I think, should be changed. Currently, the data are consistent with the function of Atg23 as an inhibitor of Atg9 vesicle fusion and Atg2 interaction, which may not involve Atg23 function as a coat protein.

Response:

First of all, we thank this referee for the positive evaluation and valuable comments. As pointed out, our data do not provide direct evidence that Atg23 functions as a structural "coat" protein. Therefore, our original description was overstated. We have revised the title accordingly and only discussed this possibility as a speculative model in the Discussion section. The revised title is "A mechanism blocking aberrant fusion ensures Atg9 vesicle targeting to autophagosome formation sites".

(2) The authors propose but do not provide evidence for the claim that Atg9 vesicles fuse with 'non-autophagic' membranes in the absence of Atg23 binding. It is equally possible that Atg9 vesicles homotypically fuse with each other (which could be considered autophagic membranes) to form bigger and potentially less mobile structures, which might be excluded from the current analyses. Such structures may be related to the Atg9 compartments observed upon Atg9 overexpression in Mari et al. 2010 JCB. The authors should at least discuss both possibilities or, better, provide experimental evidence for one or the other or both.

Response:

We agree that we were unable to determine which membranes Atg9 vesicles fuse with in the

absence of the interaction between Atg9 and Atg23, and that homotypic fusion of Atg9 vesicles is also a plausible possibility. Therefore, throughout the manuscript, we have replaced expressions such as “fusion with non-autophagic membranes” with more neutral terms such as “aberrant” or “inappropriate” fusion.

As commented on figure 3A/B below, in this context, it would be important to include the diffusing and immobile Atg9 structures in the analyses, especially, for the Atg9 mutants. Furthermore, as suggested, we have added quantification of immobile puncta to all datasets related to Atg9 vesicle quantification, including those shown in Fig. 3A and 3B.

Response:

In the revised manuscript, we have included quantification of immobile puncta in all datasets related to Atg9 vesicle quantification (Fig. 3A-L and Fig. EV2C, D, G, H).

(3) A strength of the study is the use of a number of clever genetic tools to enable the analysis of intermediate steps in the process of PAS/autophagy protein assembly during autophagosome biogenesis. However, at the same time, this is also a potential weakness, because these genetic tools may or may not fully reflect the intermediates observed in an unperturbed process. Thus, it would be important to complement these approaches and more thoroughly assess the role/effects of Atg23 or Atg9 variants in an otherwise WT background with a direct readout for PAS assembly, autophagosome biogenesis, and autophagy flux.

Response:

We agree with this reviewer's comment. We have examined the PAS localization of Atg2 and

Atg14 in cells expressing Atg9-GNb and Atg23-GFP without additional *ATG* knockout, and the results were consistent with those obtained in the *atg5*Δ background (Fig. EV5B, C, F, G). We have also analyzed autophagic bodies within the vacuole by electron microscopy in *atg23*Δ cells and cells expressing Atg9 mutants (Fig. 4E, F). These analyses revealed significant changes in both the number and size of autophagic bodies in *atg23*Δ and Atg9 mutant cells, demonstrating the importance of Atg23 and the Atg9-Atg23 interaction in autophagosome biogenesis. Furthermore, to more carefully assess autophagy flux, in addition to the Pgk1-GFP processing assay (Fig. 4C, D and Fig. EV3C, D), we performed the GFP-Atg8 processing assay (Fig. EV3E, F) and the Pho8Δ60 alkaline phosphatase (ALP) assay (Fig. EV3G). The results were consistent with those obtained using the Pgk1-GFP processing assay.

(4) Key experiments that, I think, are missing: Do Atg9S19AS948A and/or Atg9S19DS948D affect Atg9-Atg23 colocalization in vivo? Experiments similar to figure 1A/B should be performed. Atg23 has been shown to bind to membranes (Hawkins et al. 2022, s. below), thus, Atg23 might still bind to Atg9SD vesicles.

Response:

We thank the reviewer for this important suggestion. To address this point, we examined the colocalization between Atg9 and Atg23 in vivo by fluorescence microscopy (Fig. EV2P, Q). We observed that most Atg9$^{S19A\ S948A}$-GFP and Atg9$^{S19D\ S948D}$-GFP puncta (Atg9 vesicles) were positive for Atg23-mCherry, similar to Atg9^{WT}-GFP. Thus, as this reviewer anticipated, in vivo, the association of Atg23 with Atg9 vesicles is not abolished even in the Atg9$^{S19D\ S948D}$ mutant, which was suggested by co-immunoprecipitation analysis to lose the interaction with Atg23. We have added a discussion on the possibility that the Atg9$^{S19D\ S948D}$ mutant still retains sufficient

interaction with Atg23 to associate with Atg9 vesicles in the intracellular environment, or that direct Atg23 binding to the membranes of Atg9 vesicles may contribute to this association, citing Hawkins et al. (2022) (Lines 224-232).

How do Atg9S19AS948A and/or Atg9S19DS948D affect Atg9 localization to Atg17-marked PAS? These experiments should also include an atg23 deletion background to test for epistasis relationships between Atg23 and Atg9 mutations.

Response:

We assessed the PAS localization of the Atg9 mutants by quantifying their colocalization with Atg17-mCherry in cells expressing the Atg9 mutants and showed that the mutations did not significantly affect this process (Fig. EV3A, B), suggesting that the interaction between Atg9 and Atg23 does not substantially influence the PAS localization of Atg9 vesicles (given these results, we did not proceed with epistasis analysis with *ATG23* deletion). In contrast, consistent with previous reports (PMID: 25385507 and 35443167), Atg9 PAS localization was markedly reduced in *atg23*Δ cells. This is likely because *ATG23* deletion causes a greater reduction in the number of Atg9 vesicles than the Atg9 mutants (Fig. 3C, D), due to more severe defects in both Atg9 vesicle biogenesis and the blocking of aberrant fusion of Atg9 vesicles before their localization to the PAS.

Detailed comments:

The authors need to indicate how many cells were analyzed in each experiment.

Response:

We have now indicated the number of cells analyzed for each experiment in Source Data.

Figure 1A-C should include the data for non-treated cells to confirm Atg9 and Atg23 colocalize in the absence of autophagy induction.

Response:

We have included data for non-treated cells in Fig. 1A–C. The results showed that Atg9 and Atg23 colocalize even in the absence of autophagy induction.

Figure 2A/B: input protein levels seem to reflect pulldown efficiencies. Authors need to quantify protein input levels with proper loading controls and, if needed, normalize pulldowns accordingly.

Response:

As the reviewer pointed out, the Atg9-GFP band in *atg2*Δ cells appears reduced in the input samples. We considered this to be an apparent reduction caused by Atg9-GFP phosphorylation by Atg1, which caused band smearing. Indeed, as shown in Fig. EV1C, λ-phosphatase treatment of the input samples restored the Atg9-GFP band intensity to a level comparable to that in other cells, including wild-type cells.

Figure 2G and H: It is counterintuitive that the authors treated cells with rapamycin prior to pulldown experiments. Wouldn't these experiments be more informative to test whether these changes in Atg9 are sufficient for affecting Atg23 binding. In the presence of rapamycin, Atg1 is activated and might phosphorylate additional sites aside from the mutated once. These

experiments should be repeated without the addition of rapamycin.

Response:

We performed coimmunoprecipitation experiments using cells without rapamycin treatment and obtained results similar to those in rapamycin-treated cells (Fig. 2G, H).

Figure 2H: It might be worth testing whether deletion of Atg1 affects binding of Atg9 variants to Atg23 examining whether other kinases and/or sites are a factor.

Response:

To address this point, we compared interactions between Atg9 variants and Atg23 in *atg1*Δ cells and found no substantial differences between wild-type Atg9 and the Atg9 mutants (Fig. R1). The result that the S19A S948A mutation did not increase the Atg9-Atg23 interaction in *atg1*Δ cells is consistent with the notion that an additional kinase is unlikely to be involved in the phosphorylation of these Ser residues (if another kinase phosphorylates these residues to downregulate the interaction, the S19A S948A mutation would increase the interaction). Meanwhile, if phosphorylation of other residues by Atg1 also contributes to downregulation of the interaction, the interaction between the S19D S948D mutant and Atg23 might be detected in *atg1*Δ cells. However, this was not the case. Thus, this result does not suggest the involvement of additional phosphorylation sites. As these results do not lead to a clear conclusion, we have not included them in the manuscript.

Fig. R1

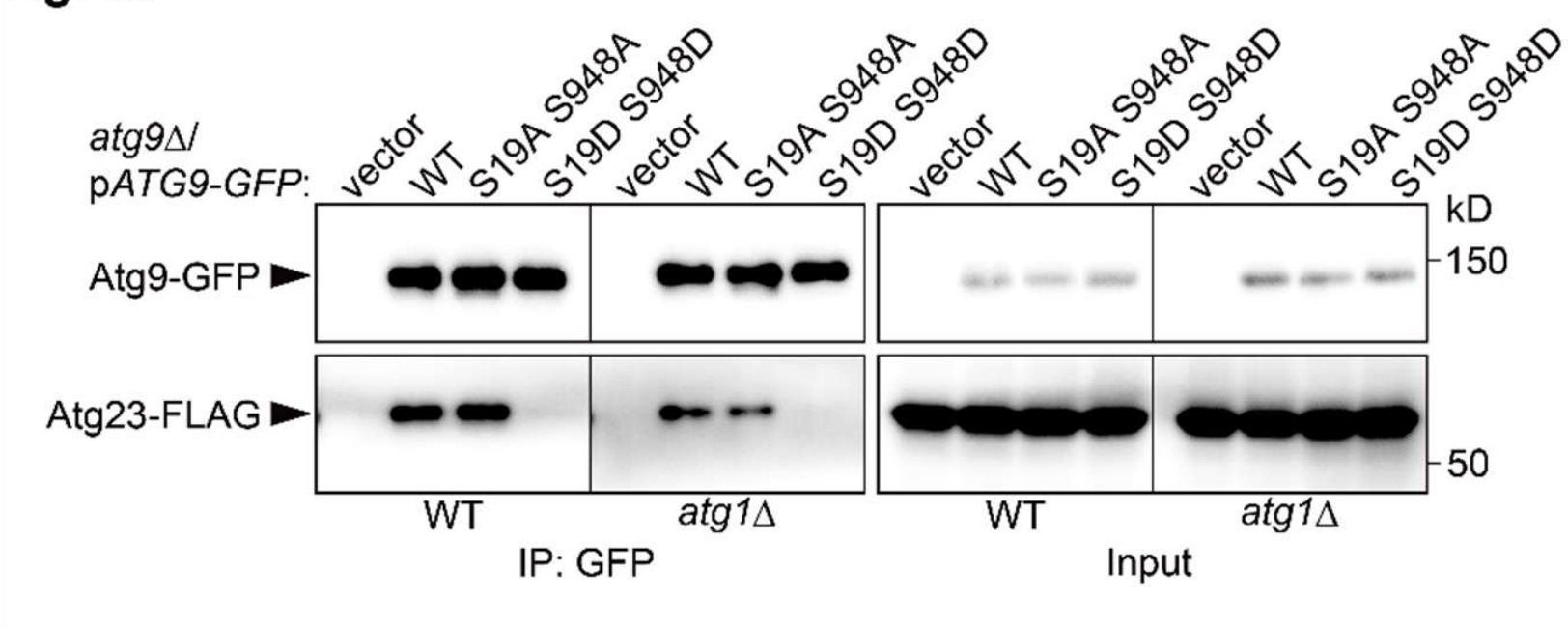

Figure. R1

Cells expressing Atg9-GFP and Atg23-FLAG were treated with rapamycin for 2 h. Cell lysates (Input) were subjected to immunoprecipitation using GNb-conjugated beads. The immunoprecipitates (IP) were analyzed by immunoblotting with antibodies against FLAG and GFP.

Figure 3A/B: What is the logic of removing immobile Atg9 puncta here and not in figure 1? I think it would be helpful if the authors explained their rationale in the text (not just in material and methods).

Response:

In Fig. 1A and B, our aim was to confirm Atg23 association with most Atg9 vesicles, which have been suggested but not directly assessed in previous studies. Since most Atg9 puncta represent Atg9 vesicles in *atg11*Δ *atg17*Δ cells, we did not distinguish between mobile and immobile Atg9 puncta in this analysis. In contrast, in Fig. 3, to more carefully examine the number of Atg9 vesicles (diffusing puncta), we excluded immobile puncta, which likely represent Atg9 localized to the Golgi or endosomes. We have clarified this rationale in the revised manuscript (lines 166-169).

Additionally, the authors should quantify both, immobile and diffusing puncta in all key genotypes. This is particularly important for the atg9 mutant variants because they have not been previously analyzed in this way.

Response:

As requested, we have included representative images and quantification of immobile puncta in all experiments examining the number of Atg9 vesicles (Fig. 3A–L and EV2C, D, G, H).

Line 217-220: "The results showed that atg9S19AS948A mutant cells are significantly defective in autophagosome formation". The data do not allow this conclusion, and it is an overinterpretation of the data. The authors can only conclude that the mutation reduces autophagy turnover. The reason could be autophagosome biogenesis, fusion, or degradation in the vacuole. However, the authors have not tested the different possibilities directly. Thus, the authors should measure the formation of autophagosomes in these strains to support their conclusion.

Response:

We agree that the Pgk1-GFP cleavage assay is insufficient to conclude that the Atg9 mutant is defective in autophagosome formation. In the revised manuscript, we observed autophagic bodies by electron microscopy in cells expressing the Atg9 mutants (Fig. 4E, F). The Atg9 S19A S948A mutation markedly reduced the number of autophagic bodies, along with a decrease in their size. In contrast, cells expressing the Atg9 S19D S948D mutant, which is defective in the interaction with Atg23, exhibited a modest increase in autophagic body size and a reduction in the number, a phenotype similar to that observed in *atg23*Δ cells. We did not observe an

accumulation of autophagosomes in the cytoplasm in these cells. These results suggest that the reduced autophagic activity in these Atg9 mutants is most likely due to defects in autophagosome formation.

Moreover, the atg9S19AS948A mutant displays reduced coimmunoprecipitation for Atg23 in figure 2H compared to Atg9, suggesting increased not decreased dissociation. Thus, these data are in conflict with the conclusion and proposed model. Is the atg9S19AS948A mutant only affected by reduced Atg23 binding or may other factors play a role? The authors should compare Pgk1-GFP turnover in Atg9 and atg9S19AS948A in atg23 deficient cells.
Figure 4C/D should include atg23 mutant background comparisons to test for epistasis.

Response:

Although the original immunoprecipitation data in Fig. 2H appeared to show reduced interaction between the Atg9 S19A S948A mutant and Atg23, in repeated experiments, the interaction was largely unchanged, and we have replaced the data accordingly. Meanwhile, we also more carefully examined the possibility that this mutation affects functions of Atg9 other than its interaction with Atg23. Structural predictions using AlphaFold3 suggest that these serine residues are directly involved in Atg23 binding (Fig. EV1E). Therefore, alanine substitution at these positions is likely to specifically impair the interaction rather than globally affecting the Atg9 structure. In addition, following the referee's suggestion, we performed Pgk1-GFP and GFP-Atg8 processing assays in the *atg23*Δ background (Fig. EV3C–F). Whereas the Atg9 S19A S948A mutant showed reduced activity in these assays compared to wild-type Atg9, we observed little difference in the *atg23*Δ background, suggesting that the reduced autophagic activity in the Atg9 S19A S948A mutant is attributable to the persistent association of Atg23

with Atg9 rather than other defects in Atg9 function.

Figure 4E: the authors need to mention the atg5 deficient strain background being used and explain the rationale in the text.

Response:

We have described the use of the *atg5*Δ background and explained the rationale for this approach (lines 314-315).

The authors should assess Atg9 and Atg2 interactions directly in dependence of Atg23 dissociation. For example, does Atg9 and Atg2 interact less in atg9S19AS948A and more in atg9S19DS948D mutant cells?

Response:

To address this point, we examined the interaction between Atg9 and Atg2 by in cells expressing the Atg9 mutants as well as cells coexpressing Atg9-GFP and Atg23-GNb (Fig. EV5H, I). In both of these cells, we observed no substantial changes in the interaction between Atg9 and Atg2. These results suggest that Atg23 association/dissociation does not directly regulate the Atg9–Atg2 interaction. Thus, the mechanism by which persistent retention of Atg23 at the PAS reduces Atg2 localization to the PAS remains unclear. We have discussed this point as follows: Atg18 binding to PI3P is known to play a pivotal role in the PAS localization of Atg2 (the Atg2-Atg18 complex). Therefore, persistent association of Atg23 may limit Atg18 access to PI3P on Atg9 vesicles, thereby impairing recruitment of the Atg2–Atg18 complex to the PAS (lines 397-407).

Figure 4I/J: the authors need to include ATG9-GNb + GFP or protein-GFP control other than Atg23-GFP to control for unspecific steric inhibition. Additionally, it is important to test atg9S19DS948D -GNb + Atg23-GFP.

Response:

We thank this referee for this important suggestion. Accordingly, we performed additional control experiments. Expression of GFP alone together with Atg9-GNb did not reduce autophagic activity, suggesting that the Atg23 moiety of Atg23-GFP is responsible for autophagy inhibition (Fig. 5C, D). However, Atg23-GFP reduced autophagic activity even in cells expressing Atg9$^{S19D\ S948D}$-GNb, which is defective in interaction with Atg23, similarly to cells expressing wild-type Atg9-GNb (Fig. EV5D, E). These results suggest that Atg23 interferes with PAS recruitment of Atg2 by steric hindrance rather than by inducing a conformational change in Atg9 through its interaction with Atg9. We have added this discussion in lines 323–328.

The authors should discuss the recent publication directly relevant for their study: Hawkins et al. (2022), Cell Reports

Response:

We have citied Hawkins et al. (2022) in the discussion of the membrane-binding ability of Atg23 (lines 230–232) and its structural features (lines 379–382) in the revised manuscript.

Referee #2:

In this study, Takumi et al. elegantly demonstrated how Atg1 and Atg23 cooperate to ensure the proper targeting of Atg9 vesicles for autophagosome biogenesis. Through a series of well-designed and rigorous experiments, the authors addressed a long-standing question in the field of autophagy. Their results revealed that Atg23 suppresses SNARE-mediated fusion of Atg9 vesicles with non-autophagic membranes. Notably, they also uncovered a dynamic regulatory mechanism of autophagy: upon arrival of Atg9 vesicles at the phagophore assembly site, the kinase Atg1 phosphorylates the Atg9 region that interacts with Atg23, thereby promoting the recruitment of the downstream factor Atg2. Overall, the study is conceptually clear, methodologically sound, and supported by solid experimental data. It represents a fascinating and important contribution to the field. However, I have several questions that I would like the authors to clarify.

(1) In Figure 2C, phosphorylation of Atg9 by Atg1 appears to affect the protein level of Atg9, whereas the Atg1 kinase-dead mutant does not. Previous studies have reported that Atg1 indeed regulates the proteasomal degradation of Atg9, and the authors should discuss this point in the Discussion section. Moreover, in Figure 2A, deletion of Atg2 also leads to a marked decrease in Atg9 protein levels. It is unclear whether this observation reflects a true biological effect. Could the loss of Atg2 influence Atg1 kinase activity, or might there be another underlying mechanism?

Response:

First, we thank this referee for the positive evaluation of our study.

Regarding this comment, we considered the possibility that the apparent reduction in Atg9-GFP

levels in cells expressing Atg1-Gcn4^{CC}-GNb is due to band smearing and/or reduced transfer efficiency in immunoblotting caused by phosphorylation. Indeed, as shown in Fig. EV1D, λ-phosphatase treatment of the samples sharpened the Atg9 band and restored the band intensity to a level comparable to that in other strains. In *atg2*Δ cells, Atg9 accumulates at the PAS, where it is phosphorylated by Atg1, leading to the accumulation of phosphorylated Atg9. Similarly, λ-phosphatase treatment restored the Atg9 band intensity in *atg2*Δ cells (Fig. EV1C).

(2) In Figure 1E, the authors should clarify whether the phenotype of the Atg1 kinase-dead mutant resembles that of the Atg1 KO strain. This comparison is essential to confirm that the colocalization of Atg9 and Atg23 with the PAS is indeed regulated through the kinase activity of Atg1.

Response:

We have compared the PAS localization of Atg9 and Atg23 between the Atg1 kinase-dead mutant (Atg1^{D211A}) and the Atg1 knockout strain (vector) (Fig. 1G–I), and showed that as in the Atg1 KO strain, both Atg9 and Atg23 accumulate at the PAS visualized by Atg17-mCherry puncta in the kinase-dead mutant, confirming that the colocalization of Atg9 and Atg23 at the PAS is regulated by the kinase activity of Atg1.

(3) In Figure 2G, the authors show that mimicking Atg1-mediated phosphorylation of Atg9 at S19 and S948 (S19D/S948D) significantly reduces the interaction between Atg9 and Atg23, clearly supporting the model that Atg1 phosphorylation promotes Atg9-Atg23 dissociation. However, it is puzzling that the non-phosphorylatable mutant (S19A-S948A) also shows a notable reduction in Atg23 binding. The authors should address this unexpected result in the

Discussion. Could it be that the S19A-S948A mutation alter the structural conformation or stability of Atg9, thereby indirectly affecting its interaction with Atg23?

Response:

Although the original immunoprecipitation data in Fig. 2H appeared to show reduced interaction between the Atg9 S19A S948A mutant and Atg23, in repeated experiments, the interaction was largely unchanged, and we have replaced the data accordingly. Meanwhile, structural predictions using AlphaFold3 suggest that these serine residues are directly involved in Atg23 binding (Fig. EV1E). Therefore, alanine substitution at these positions is likely to specifically impair the interaction rather than largely altering the Atg9 structure. We have added this discussion in the revised manuscript (lines 392–397).

(4) In Figures 4C and 4D, given that the S19D-S948D mutant markedly reduces Atg9-Atg23 interaction and promotes Atg2 recruitment, one might expect enhanced autophagic activity. However, the autophagy activity of the S19D-S948D mutant appears even lower than that of the wild type. The authors should provide a possible explanation for this discrepancy.

Response:

As this reviewer pointed out, the Atg9 S19D S948D mutant seems to promote the PAS recruitment of Atg2. However, the Atg23-Atg9 interaction is important for the formation of Atg9 vesicles (PMID: 17426440, 22826123, and 35443167). In addition, we showed in this study that this interaction is also important for preventing aberrant fusion of Atg9 vesicles during their localization to the PAS. Consistently, we showed that the number of Atg9 vesicles is reduced in the S19D S948D mutant (Fig. 3C, D). Therefore, this mutation impairs autophagy

probably due to defects in Atg9 vesicle formation and proper trafficking (lines 177–179 and 199–201).

Referee #3:

The manuscript by Kimura et al report a new role of Atg23 in regulating Atg9 vesicle trafficking. The authors noticed that in atg2D cells, although Atg9 accumulated at the PAS consistent with existing knowledge, Atg23 (an interactor of Atg9) did not display such accumulation. The authors hypothesized that Atg23 might dissociate from Atg9 vesicle resulting from weakened interaction, and found that Atg23 co-immunoprecipitated mildly more Atg9 in atg1D, and mildly less Atg9 in atg2D. In addition, Atg9 band displayed some upshift in atg2D, pointing a possibility of phosphorylation. Previous works reported Atg9 as a substrate of Atg1. The authors used a nanobody based system to tether Atg1 to Atg9, and found that forced tethering led to strong phosphorylation of Atg9 and loss of Atg23-Atg9 interaction in CoIP. They further confirmed that S-D substitution of two of the potential phosphorylation sites, S19 and S948, strongly reduced Atg23-Atg9 CoIP.

The authors then explored the potential functional consequence of the phosphorylation. Using the same Atg1 tethering tool, the authors found that forced tethering of Atg1 to Atg9 reduced the number of visible Atg9 vesicles. This reduction was reproduced by the SD mutant, and abolished by the SA mutant, consistent with a role of phosphorylation. Interestingly, inhibiting the function of NSF(Sec18) led to moderate recovery of Atg9 vesicle number in the presence of Atg1 tethering, and in atg23D cells. Knocking out a SNARE protein also partially recovered Atg9 vesicle number. The authors interpreted the data as Atg23 prevents unintended fusion of Atg9 vesicle to non-PAS membrane.

Another function the authors proposed is that the release of Atg23 promotes recruitment of Atg2 to move forward in autophagosome biogenesis. The authors observed in atg2D cells that SA substitutions in Atg9 led to a mild increase in the presence of Atg23 at PAS (marked by Atg17). In atg5D cells, they found a mild decrease of Atg2 dots at PAS with the SA allele. Forced tethering of Atg23 to Atg9 (via the nanobody technique) led to a clear reduction of Atg2 dots at PAS, but did not affect the PI3K complex. The authors also observed a major reduction of autophagic substrate degradation (Pgk1) in SA mutant cells, and a mild reduction in SD mutant cells.

Overall, this is a very interesting story. How various Atg proteins move step by step to participate in autophagosome biogenesis is a challenging and poorly explored aspect in mechanistic studies of autophagy. Here, the authors' work has the potential to make a key contribution. However, the current manuscript contains several weakness that need to be addressed, and feels preliminary in its current form.

Major issues:

1. My biggest confusion is on how important the reported phosphorylation is in the big picture. The authors demonstrated nicely that phosphorylation of Atg9 by Atg2 can strongly inhibit Atg23-Atg9 interaction. This is a very strong effect. It appears that SA allele of Atg9 interacts constitutively with Atg23, and SD allele of Atg9 essentially loses the interaction. The authors also reported a large reduction of Atg9 vesicle number in SD mutant, and a mild reduction of Atg2 dots in SA mutant. On the other hand, it was the SA allele that produced a major reduction in the degradation of Pgk1, with the SD having only a mild reduction. In other words, from the end result, the authors are essentially saying that loss of Atg23 interaction (SD allele, strong reduction of Atg9 vesicle number) is not important for autophagic flux, whereas constitutive

interaction is strongly inhibitive (but without an explanation that match the scale).

Response:

First, we thank this referee for the positive and constructive comments.

Regarding this comment, as the referee pointed out, the S19D S948D mutant showed a strong reduction in the Atg9–Atg23 interaction in coimmunoprecipitation assays, and thus might be expected to cause a more severe defect in autophagy than the S19A S948A mutant. While the immunoprecipitation was performed under membrane-solubilizing conditions, newly added fluorescence microscopy analysis showed that colocalization between Atg9 and Atg23 was largely preserved even in the Atg9 S19D S948D mutant (Fig. EV2P–Q). These results suggest that the interaction between the Atg9 S19D S948D mutant and Atg23 is not completely abolished in cells and/or that Atg23 remains associated with Atg9 vesicles through direct interactions with lipid membranes (PMID: 35443167), thereby compensating the reduced Atg9-Atg23 interaction. As the referee mentioned, the S19D S948D mutant exhibited a significant reduction in the Atg9 vesicle number compared with the S19A S948A mutant. However, we found that Atg9 S19D S948D vesicles localize to the PAS comparably to wild-type Atg9 vesicles (Fig. EV3A–B). Thus, the reduction of autophagy activity in the $Atg9^{S19D\ S948D}$ mutant appears less severe than expected from the reduction in the interaction with Atg23 observed in coimmunoprecipitation analysis. We have described and discussed these results in lines 223–232 and 256–262.

Using a single substrate (Pgk1) to report autophagic flux has the potential to generate bias. I suggest that the authors conduct the more quantitative pho8D60 assay, and the more commonly used GFP-Atg8 processing assay, to allow better interpretation of the resulting autophagic flux

and cross comparison with other yeast autophagy work. The significance of excess Atg23-Atg9 interaction and loss of interaction may appear different with these assays.

Response:

In response to this reviewer's comment, we performed the GFP-Atg8 processing assay and the Pho8Δ60 assay. The results obtained from both assays were consistent with those from the Pgk1-GFP processing assay, although the reduction in autophagic activity in the Atg9 S19D S948D mutant was more pronounced in the Pho8Δ60 assay (Fig. 4C, D and EV3C–G). These results provide a more comprehensive assessment of autophagic flux, further supporting our conclusions.

Along this line, as Atg9 was reported to affect autophagosome number instead of size, I suggest that the authors perform TEM to dissect whether reduced autophagic flux resulted from reduced autophagosome number in the context of disturbed Atg23-Atg9 interaction. To facilitate cross comparison, atg23D should also be included as a control in these assays.

Response:

To address this point, we performed TEM to observe autophagic bodies within vacuoles (Fig. 4E, F). The results showed that the number of autophagic bodies significantly reduced in both the Atg9 S19A S948A and S19D S948D mutants, supporting the idea that reduced autophagic flux resulted from decreased efficiency of autophagosome formation. We also observed small changes in the size of autophagic bodies: a decrease in the S19A S948A mutant and an increase in the S19D S948D mutant. We also showed that the size of autophagic bodies increases in *ATG23* knockout cells. This is consistent with the observation that the S19D S948D mutation

impairs the Atg9-Atg23 interaction. We have described these results in lines 270-283.

2. Assuming that additional assays of autophagic flux suggested above end up reporting results consistent with the Pgk1 result, then I have the question of why a strong reduction of Atg9 vesicle number did not lead to a matching reduction in autophagosome number with the SD allele or Atg1 tethering. The authors should examine the presence of Atg9 vesicle at PAS (the existing data are about non-PAS vesicles) to see if such a level of reduction in total number is actually tolerable for the cells to maintain a close to normal presence of Atg9 at the PAS, which might explain such discrepancy.

Response:

In response to this suggestion, we examined the PAS localization of the Atg9 mutants, and the results showed that both the S19A S948A and S19D S948D mutants localize to the PAS comparably to wild-type Atg9 (Figs. EV3A, B). Therefore, as the referee expected, the reduction in the number of Atg9 vesicles in the S19D S948D mutant would not be sufficient to substantially reduce the amount of Atg9 vesicles localized at the PAS, which may explain why the effect on autophagic flux is relatively modest. We have described these results in lines 243-245 and 259-262.

Note that the authors tend to use various atg knockouts to arrest autophagosome biogenesis (see also latter discussion). It is critical that a comparison under close-to-WT genotype should be included, beside any atg knockouts that may facilitate the observation.

Response:

We have addressed this issue in our response to related comments below, where we include analyses performed in a close-to-WT background.

3. The authors propose that phosphorylation of Atg9 allows the release of Atg23, and in turn recruitment of Atg2. Based on a recent study (PMID: 39715823), there is only a very short time window of about 1 min from the formation of Atg17 dots to the formation of Atg2 dots. The authors' model imply that it is this short period that Atg23 reside at the PAS. To provide direct support for the authors' hypothesis, I suggest that the authors observe by time-lapse imaging to pinpoint when Atg9 and Atg23 arrive and leave the PAS relative to other key Atg proteins, in particular Atg2.

Response:

To address this issue, we performed time-lapse imaging analyses (Fig. EV4A, B). Following the approach described in the study mentioned in this comment, we conducted time-lapse imaging of Atg23-GFP and Atg2-GFP relative to mCherry-Atg8 (we were unable to reliably determine the timing of Atg9 puncta formation). We found that Atg23 puncta appeared transiently prior to the formation of mCherry-Atg8 puncta and disappeared shortly thereafter. In contrast, Atg2 puncta appeared before or concomitantly with mCherry-Atg8 puncta and remained colocalized for a longer period. These results support our model that Atg23 transiently resides at the PAS and dissociates prior to the recruitment of downstream factors such as Atg2.

Minor issues:

1. The coat hypothesis is very interesting, but I have some reservation. The authors should at least provide quantitative data on the number of Atg23 molecules present at each Atg9 vesicle

and see how it compares with the number of Atg9 molecules. There are other possible mechanisms that can explain the current observation. Examining and ruling out the alternatives is outside the scope of this work. But I think the authors need stronger evidence, such as cryoEM, to make such a claim.

Response:

As also pointed out by Reviewer #1, we agree that our current data are not sufficient to conclusively support the coat hypothesis. As suggested by Reviewer #1 and after discussion with the editor, we have decided to moderate our claims. Specifically, we have revised the title to "A mechanism blocking aberrant fusion ensures Atg9 vesicle targeting to autophagosome formation sites" and limited our discussion of the coat hypothesis to the Discussion section.

2. Fig. 3A-N, the authors described a method of removing immobile Atg9 dots in text. But it is not immediately evident what images are displayed here. Are these raw images, or computed images only showing the mobile dots? Also, given the reported number, I guess the authors were quantifying dots per slice, not the total number of Atg9 dots per cell that would require the capturing of z-stacks. In either case, more explicit statements are needed.

Response:

The images shown in Fig. 3A–L (in the revised version) are raw images. The quantification represents the number of puncta per z-slice, rather than the total number per cell. We have clarified these points in the main text (lines 169–175). In addition, following Reviewer #1's suggestion, we have included images and quantification of immobile puncta in all datasets related to Atg9 vesicle quantification (Fig. 3A–L and Fig. EV2C, D, G, H).

3. In Fig. 4, the authors used atg2D and atg5D cells when observing Atg23 and Atg2 localization at PAS. The effect of Atg9 phosphorylation on their presence should also be examined in a close-to-WT genotype to rule out the possibility that this regulation is only important under conditions that arrest autophagosome biogenesis, and much less important under normal conditions (as when measuring autophagic flux). The use of these mutants should be also be clearly stated in the main text when reporting the results (starting from line 205).

Response:

To address this issue, we examined the PAS localization of Atg2 and Atg14 in cells coexpressing Atg9-GNb and Atg23-GFP without additional ATG knockout (Fig. EV5B, C, F, G), and obtained results consistent with those observed in the *atg5*Δ background. We have also stated the use of *atg5*Δ backgrounds and the rationale for their use in the main text (lines 314-315).

4. Please add immunoblots of Atg9 along fluorescent images in Fig.3 to rule out the possibility that the reduction of Atg9 vesicle number is caused by protein instability.

Response:

We confirmed by immunoblotting that the levels of Atg9 are largely unchanged in the cells used in Fig. 3 (Fig. EV2B, I, J, M, N, O).

5. In Fig. EV1A, it appears that the accumulation of Atg9 was much stronger in atg1D cells than in atg2D cells. This matches our general impression when working with them. However, in Fig.

1D, the signal of Atg9 in atg1D cells appeared quite dark instead. The cytosolic background also appeared weak in this image. Did the authors use auto-contrast when generating the image in Fig. 1D? Auto-contrasting function of software will make images with bright objects appear this way. Fig. EV1B may also have this problem. If the authors used any form of auto-contrast/brightness, please go back to raw data and manually set a fixed display threshold, and check other images in the manuscript.

Response:

We did not use auto-contrast when generating these images. However, we noticed that the contrast settings were not consistently applied across images. We have now reprocessed the images from the raw data using consistent display settings and have updated the figures accordingly (Fig. 1D and Fig. EV1B). We thank the reviewer for this helpful comment.

6. Please quantify the total amounts of Atg9 in Fig. 2A. It appears that the amount was lower in atg2D.

Response:

Regarding this issue, we considered the possibility that the apparent reduction in Atg9-GFP levels in *atg2*Δ cells is due to band smearing and/or reduced transfer efficiency in immunoblotting caused by phosphorylation, because in *atg2*Δ cells, Atg9 accumulates at the PAS, where it is phosphorylated by Atg1, leading to the accumulation of phosphorylated Atg9. Indeed, as shown in Fig. EV1C, λ-phosphatase treatment of the samples sharpened the Atg9 band and restored the band intensity to a level comparable to that in other strains. The reduction of Atg9-GFP in cells expressing Atg1-Gcn4^{CC}-GNb can be explained similarly (Fig. EV1D).

7. Line 41-44, the description of the six functional groups is somewhat unconventional here. The Atg16 complex is rarely used as the name for the Atg12 conjugation system. Also, in light of the many functions of Atg1/ULK1 at late stages of autophagosome formation, and its localization pattern that covers autophagosomes instead of focusing on a dot, the continued use of "Atg1 complex" is potentially misleading.

Response:

We have replaced “Atg16 complex” with “Atg12 conjugation system” (line 44–45). We have also replaced “the Atg1 complex” with "the Atg1 kinase and its associated factors (Atg13, Atg17, Atg31, and Atg29) (hereafter referred to as the Atg1 complex)" (line 42–44). However, if the reviewer has a specific suggestion for preferred wording, we would appreciate further guidance.

8. Given the mild effect of Atg9 SA mutants on Atg2 recruitment, and the strong effect in autophagic flux, I suspect that there are other more important processes yet to be discovered. This mismatch should be discussed.

Response:

We thank for this important comment. Accordingly, we have added the following discussion in lines 408-417: “Atg23 dissociation from Atg9 vesicles may also facilitate downstream events during PAS organization, including the recruitment of factors other than Atg2. Previous studies suggested that several Atg9 vesicles assemble at the PAS, serving as a seed for the formation of a single autophagosome (Yamamoto et al, 2012). In addition, COPII vesicles are recruited to the

PAS and eventually contribute to autophagosomal membranes (Shima et al, 2019). These findings suggest that Atg9 vesicles undergo fusion at the PAS in a homotypic and/or heterotypic manner. The release of Atg23 from Atg9 vesicles could also be a prerequisite for these fusion events at the PAS. The pronounced reduction in autophagic activity in the Atg9S19A S948A mutant may reflect defects in these processes in addition to decreased recruitment of Atg2 (and other factors) to the PAS.

9. Can the authors explain why the AID tool OsTIR1 is expressed by the Z4EV promoter (line 188)?

Response:

We have added a description that we used a strain in which expression of OsTIR1 is controlled by the β-estradiol-inducible Z4EV promoter to minimize basal OsTIR1 expression and thereby prevent unintended degradation of Sec18-AID (lines 207-208).

Dear Prof. Nakatogawa,

We have now received re-review reports from two referees, which I have included below. As you will see, you have addressed their concerns satisfactorily; however, I would like you to consider addressing their remaining points in the discussion section. Before I can finally accept the manuscript, there are some remaining editorial points which need to be addressed. In this regard would you please:

- declare employment in a biotech company in the Disclosure and competing interests statement,
- remove the AC/CrediT section from the text,
- rename "Supplemental Figure Legends" as "Expanded View Figure Legends",
- ensure that images in EV2C are correctly selected from the Source Data already uploaded,
- describe the nature of entity for 'n' in the legends of figures 1B,F,I, and
- use the following section order: Title page - Abstract - Introduction - Results - Discussion - Methods - Data Availability - Acknowledgements - Disclosure and Competing Interests Statement - References - Figure Legends - Table(s) - Expanded View Figure Legends.

I am looking forward to receiving your revised manuscript.

EMBO Press is an editorially independent publishing platform for the development of EMBO scientific publications.

Best wishes,

William

William Teale, PhD
Editor
The EMBO Journal
w.teale@embojournal.org

Instructions for preparing your revised manuscript:

Read our guidance for manuscript revisions and related editorial policies: https://link.springer.com/journal/44318/submission-guidelines#cms-Revised-submissions

Please also check that the title and abstract of the manuscript are brief, yet explicit, even to non-specialists.

When assembling figures, please refer to our figure preparation guideline in order to ensure proper formatting and readability in print as well as on screen:
https://media.springernature.com/original/springer-cms/rest/v1/content/27825798/data/v1

IMPORTANT: When you send the revision we will require
- a point-by-point response to the referees' comments, with a detailed description of the changes made (as a word file).
- a word file of the manuscript text.
- individual production quality figure files (one file per figure)
- a complete author checklist
- Expanded View files (replacing Supplementary Information)
- a Reagents and Tools Table as part of the Methods section

Please remember: Digital image enhancement is acceptable practice, as long as it accurately represents the original data and conforms to community standards. If a figure has been subjected to significant electronic manipulation, this must be noted in the figure legend or in the 'Methods' section. The editors reserve the right to request original versions of figures and the original images that were used to assemble the figure.

We realize that it is difficult to revise to a specific deadline. In the interest of protecting the conceptual advance provided by the work, we recommend a revision within 3 months (10th Sep 2026). Please discuss the revision progress ahead of this time with the editor if you require more time to complete the revisions. Use the link below to submit your revision:

https://emboj.msubmit.net/cgi-bin/main.plex

Referee #1:

the reviewers address all of my questions.

Referee #3:

The authors greatly improved their manuscript in the revision, and provided reasonable responses to most of my questions. However, in light of the new data provided in the revision, I suggest that the authors revise their title (and to a certain extent, their abstract) to more accurately outline their findings. To a certain degree, the last paragraph of their introduction section (page 4, line 71-75) is a better summary then their abstract.

The authors essentially had two key findings. One is that Atg23 maintains the right number of Atg9 vesicles by preventing their aberrant fusion. And the other is that phosphorylation by Atg1 weakens Atg23-Atg9 interaction, and contributes to the release of Atg23 and the occurrence of subsequent steps. However, the authors' newly added data also demonstrate that the localization of Atg9 to Atg17 positive PAS per se is not significantly reduced. This interpretation is also supported by the proper localization and activity of the PI3K complex in mutant cells, which is recruited downstream of Atg9 (although the reduced number of autophagosomes indicates some aspect of Atg9 being functionally abnormal). Therefore I am not sure that "A mechanism ... ensures Atg9 vesicle targeting to autophagosome formation sites" is an accurate expression of their findings.

Response to Reviewers' Comments

Referee #3:

The authors greatly improved their manuscript in the revision, and provided reasonable responses to most of my questions. However, in light of the new data provided in the revision, I suggest that the authors revise their title (and to a certain extent, their abstract) to more accurately outline their findings. To a certain degree, the last paragraph of their introduction section (page 4, line 71-75) is a better summary then their abstract.

The authors essentially had two key findings. One is that Atg23 maintains the right number of Atg9 vesicles by preventing their aberrant fusion. And the other is that phosphorylation by Atg1 weakens Atg23-Atg9 interaction, and contributes to the release of Atg23 and the occurrence of subsequent steps. However, the authors' newly added data also demonstrate that the localization of Atg9 to Atg17 positive PAS per se is not significantly reduced. This interpretation is also supported by the proper localization and activity of the PI3K complex in mutant cells, which is recruited downstream of Atg9 (although the reduced number of autophagosomes indicates some aspect of Atg9 being functionally abnormal). Therefore I am not sure that "A mechanism ... ensures Atg9 vesicle targeting to autophagosome formation sites" is an accurate expression of their findings.

Response:

We thank the reviewer for this insightful suggestion and agree that our previous title did not accurately reflect the main findings of the study. As suggested by the reviewer, the central findings of this work are that Atg23 prevents aberrant fusion of Atg9 vesicles and that Atg1-dependent phosphorylation weakens the Atg23-Atg9 interaction at the PAS, leading to the release of Atg23 and progression of subsequent steps. In addition, as pointed out by the

reviewer, our new data showing that the PAS localization of Atg9 tended to be reduced in the Atg9 SD mutant, but the reduction was not statistically significant, do not strongly support the idea that the fusion-preventing function of Atg23 ensures Atg9 vesicle targeting to autophagosome formation sites. We therefore revised the title to:

"Atg23 prevents aberrant fusion of Atg9 vesicles during delivery to autophagosome formation sites."

The second finding could not be explicitly incorporated into the title because of the 100-character limit. The phrase "during delivery to autophagosome formation sites" was intended to indicate that Atg23 functions during the transport of Atg9 vesicles to the PAS rather than ensuring the transport process.

We also revised the abstract and Discussion accordingly, as highlighted in blue in the revised manuscript.

2nd Revision - Editorial Decision 2nd Jul 2026

Dear Hitoshi,

I am pleased to inform you that your manuscript has been accepted for publication in the EMBO Journal.

Congratulations to you and to all involved!

Your manuscript will be processed for publication by EMBO Press. It will be copy edited and you will receive page proofs prior to publication. Please note that you will be contacted by Springer Nature Author Services to complete licensing and payment information.

You may qualify for financial assistance for your publication charges - either via a Springer Nature fully open access agreement or an EMBO initiative. Check your eligibility: https://link.springer.com/journal/44318/how-to-publish-with-us

Should you be planning a Press Release on your article, please notify the production team via this form in order to coordinate publication and release dates: https://forms.cloud.microsoft/e/eH4A8JVP6g

If you have any questions, please do not hesitate to contact the Editorial Office. Thank you for your contribution to The EMBO Journal.

Best wishes,

William

William Teale, PhD
Editor
The EMBO Journal
w.teale@embojournal.org

*****

Please note that it is The EMBO Journal policy for the transcript of the editorial process (containing referee reports and your response letters) to be published as an online supplement to each paper. If you should prefer removal of any referee-only figures included in the point-by-point response(s), e.g. because they may still be used for future publication or because they have been reproduced from published work by others, please do let us know immediately via response email.
More information is available here: https://link.springer.com/partners/embo-press/editorial-policies#Peer%20review

** Click here to be directed to your login page: https://emboj.msubmit.net

EMBO Press Author Checklist

Corresponding Author Name: Hitoshi Nakatogawa	
Journal Submitted to: The EMBO Journal	
Manuscript Number: EMBOJ-2025-122761	

USEFUL LINKS FOR COMPLETING THIS FORM
The EMBO Journal - Author Guidelines
EMBO Reports - Author Guidelines
Molecular Systems Biology - Author Guidelines
EMBO Molecular Medicine - Author Guidelines

Reporting Checklist for Life Science Articles (updated January
This checklist is adapted from Materials Design Analysis Reporting (MDAR) Checklist for Authors. MDAR establishes a minimum set of requirements in transparent reporting in the life sciences (see Statement of Task: 10.31222/osf.io/9sm4x). Please follow the journal's guidelines in preparing your
Please note that a copy of this checklist will be published alongside your article.

Abridged guidelines for figures
1. Data
The data shown in figures should satisfy the following conditions:
- the data were obtained and processed according to the field's best practice and are presented to reflect the results of the experiments in an accurate and unbiased manner.
- ideally, figure panels should include only measurements that are directly comparable to each other and obtained with the same assay.
- plots include clearly labeled error bars for independent experiments and sample sizes. Unless justified, error bars should not be shown for technical
- if n<5, the individual data points from each experiment should be plotted. Any statistical test employed should be justified.
- Source Data should be included to report the data underlying figures according to the guidelines set out in the authorship guidelines on Data

2. Captions
Each figure caption should contain the following information, for each panel where they are relevant:
- a specification of the experimental system investigated (eg cell line, species name).
- the assay(s) and method(s) used to carry out the reported observations and measurements.
- an explicit mention of the biological and chemical entity(ies) that are being measured.
- an explicit mention of the biological and chemical entity(ies) that are altered/varied/perturbed in a controlled manner.
- the exact sample size (n) for each experimental group/condition, given as a number, not a range;
- a description of the sample collection allowing the reader to understand whether the samples represent technical or biological replicates (including how many animals, litters, cultures, etc.).
- a statement of how many times the experiment shown was independently replicated in the laboratory.
- definitions of statistical methods and measures:
  - common tests, such as t-test (please specify whether paired vs. unpaired), simple χ2 tests, Wilcoxon and Mann-Whitney tests, can be unambiguously identified by name only, but more complex techniques should be described in the methods section;
  - are tests one-sided or two-sided?
  - are there adjustments for multiple comparisons?
  - exact statistical test results, e.g., P values = x but not P values < x;
  - definition of 'center values' as median or average;
  - definition of error bars as s.d. or s.e.m.

Please complete ALL of the questions below.
Select "Not Applicable" only when the requested information is not relevant for your study.

Materials

Newly Created Materials	Information included in the manuscript?	In which section is the information available? (Reagents and Tools Table, Materials and Methods, Figures, Data Availability Section)
New materials and reagents need to be available; do any restrictions apply?	Not Applicable	

Antibodies	Information included in the manuscript?	In which section is the information available? (Reagents and Tools Table, Materials and Methods, Figures, Data Availability Section)
For **antibodies** provide the following information:<br>- Commercial antibodies: RRID (if possible) or supplier name, catalogue number and or/clone number<br>- Non-commercial: RRID or citation	Yes	

DNA and RNA sequences	Information included in the manuscript?	In which section is the information available? (Reagents and Tools Table, Materials and Methods, Figures, Data Availability Section)
**Short novel DNA or RNA including primers, probes**: provide the sequences.	Not Applicable	

Cell materials	Information included in the manuscript?	In which section is the information available? (Reagents and Tools Table, Materials and Methods, Figures, Data Availability Section)
**Cell lines:** Provide species information, strain. Provide accession number in repository **OR** supplier name, catalog number, clone number, and/**OR** RRID.	Not Applicable	
**Primary cultures:** Provide species, strain, sex of origin, genetic modification status.	Not Applicable	
Report if the cell lines were recently **authenticated** (e.g., by STR profiling) and tested for mycoplasma contamination.	Not Applicable	

Experimental animals	Information included in the manuscript?	In which section is the information available? (Reagents and Tools Table, Materials and Methods, Figures, Data Availability Section)
**Laboratory animals or Model organisms:** Provide species, strain, sex, age, genetic modification status. Provide accession number in repository **OR** supplier name, catalog number, clone number, **OR** RRID.	Not Applicable	
**Animal observed in or captured from the field:** Provide species, sex, and age where possible.	Not Applicable	
Please detail **housing and husbandry conditions**.	Not Applicable	

Plants and microbes	Information included in the manuscript?	In which section is the information available? (Reagents and Tools Table, Materials and Methods, Figures, Data Availability Section)
**Plants:** provide species and strain, ecotype and cultivar where relevant, unique accession number if available, and source (including location for collected wild specimens).	Not Applicable	
**Microbes:** provide species and strain, unique accession number if available, and source.	Yes	

Human research participants	Information included in the manuscript?	In which section is the information available? (Reagents and Tools Table, Materials and Methods, Figures, Data Availability Section)
If collected and within the bounds of privacy constraints report on age, sex and gender or ethnicity for all study participants.	Not Applicable	

Core facilities	Information included in the manuscript?	In which section is the information available? (Reagents and Tools Table, Materials and Methods, Figures, Data Availability Section)
If your work benefited from core facilities, was their service mentioned in the acknowledgments section?	Yes	

Design

Study protocol	Information included in the manuscript?	In which section is the information available? (Reagents and Tools Table, Materials and Methods, Figures, Data Availability Section)
If study protocol has been **pre-registered, provide DOI in the manuscript**. For clinical trials, provide the trial registration number **OR** cite DOI.	Not Applicable	
Report the **clinical trial registration number** (at ClinicalTrials.gov or equivalent), where applicable.	Not Applicable	

Laboratory protocol	Information included in the manuscript?	In which section is the information available? (Reagents and Tools Table, Materials and Methods, Figures, Data Availability Section)
Provide DOI OR other citation details if **external detailed step-by-step protocols** are available.	Not Applicable	

Experimental study design and statistics	Information included in the manuscript?	In which section is the information available? (Reagents and Tools Table, Materials and Methods, Figures, Data Availability Section)
Include a statement about **sample size** estimate even if no statistical methods were used.	Yes	
Were any steps taken to minimize the effects of subjective bias when allocating animals/samples to treatment (e.g. **randomization procedure**)? If yes, have they been described?	Not Applicable	
Include a statement about **blinding** even if no blinding was done.	Not Applicable	
Describe **inclusion/exclusion criteria** if samples or animals were excluded from the analysis. Were the criteria pre-established?<br><br>If sample or data points were omitted from analysis, report if this was due to attrition or intentional exclusion and provide justification.	Not Applicable	
For every figure, are **statistical tests** justified as appropriate? Do the data meet the assumptions of the tests (e.g., normal distribution)? Describe any methods used to assess it. Is there an estimate of variation within each group of data? Is the variance similar between the groups that are being statistically compared?	Not Applicable	

Sample definition and in-laboratory replication	Information included in the manuscript?	In which section is the information available? (Reagents and Tools Table, Materials and Methods, Figures, Data Availability Section)
In the figure legends: state number of times the experiment was **replicated** in laboratory.	Yes	
In the figure legends: define whether data describe **technical or biological replicates**.	Yes	

Ethics

Ethics	Information included in the manuscript?	In which section is the information available? (Reagents and Tools Table, Materials and Methods, Figures, Data Availability Section)
Studies involving **human participants**: State details of **authority granting ethics approval** (IRB or equivalent committee(s), provide reference number for approval.	Not Applicable	
Studies involving **human participants**: Include a statement confirming that **informed consent** was obtained from all subjects and that the experiments conformed to the principles set out in the WMA Declaration of Helsinki and the Department of Health and Human Services Belmont Report.	Not Applicable	
Studies involving **human participants:** For publication of **patient photos**, include a statement confirming that consent to publish was obtained.	Not Applicable	
Studies involving experimental **animals**: State details of **authority granting ethics approval** (IRB or equivalent committee(s), provide reference number for approval. Include a statement of compliance with ethical regulations.	Not Applicable	
Studies involving **specimen and field samples:** State if relevant **permits** obtained, provide details of authority approving study; if none were required, explain why.	Not Applicable	

Dual Use Research of Concern (DURC)	Information included in the manuscript?	In which section is the information available? (Reagents and Tools Table, Materials and Methods, Figures, Data Availability Section)
Could your study fall under dual use research restrictions? Please check biosecurity documents and list of **select agents and toxins** (CDC): https://www.selectagents.gov/sat/list.htm	Not Applicable	
If you used a select agent, is the security level of the lab appropriate and reported in the manuscript?	Not Applicable	
If a study is subject to dual use research of concern regulations, is the name of the **authority granting approval and reference number** for the regulatory approval provided in the manuscript?	Not Applicable	

Reporting

The MDAR framework recommends adoption of discipline-specific guidelines, established and endorsed through community initiatives. Journals have their own policy about requiring specific guidelines and recommendations to complement MDAR.

Adherence to community standards	Information included in the manuscript?	In which section is the information available? (Reagents and Tools Table, Materials and Methods, Figures, Data Availability Section)
State if relevant guidelines or checklists (e.g., **ICMJE, MIBBI, ARRIVE, PRISMA**) have been followed or provided.	Not Applicable	
For **tumor marker prognostic studies,** we recommend that you follow the **REMARK** reporting guidelines (see link list at top right). See author guidelines, under 'Reporting Guidelines'. Please confirm you have followed these guidelines.	Not Applicable	
For **phase II and III randomized controlled trials**, please refer to the **CONSORT** flow diagram (see link list at top right) and submit the CONSORT checklist (see link list at top right) with your submission. See author guidelines, under 'Reporting Guidelines'. Please confirm you have submitted this list.	Not Applicable	

Data Availability

Data availability	Information included in the manuscript?	In which section is the information available? (Reagents and Tools Table, Materials and Methods, Figures, Data Availability Section)
Have **primary datasets** been deposited according to the journal's guidelines (see 'Data Deposition' section) and the respective accession numbers provided in the Data Availability Section?	Yes	
Were **human clinical and genomic datasets** deposited in a public access-controlled repository in accordance to ethical obligations to the patients and to the applicable consent agreement?	Not Applicable	
Are **computational models** that are central and integral to a study available without restrictions in a machine-readable form? Were the relevant accession numbers or links provided?	Not Applicable	
If publicly available data were reused, provide the respective **data citations in the reference list**.	Not Applicable	
