# Supplementary material for: Atg23 prevents aberrant fusion of Atg9 vesicles during delivery to autophagosome formation sites
Source: EMBO J. 2026 Jul 20;45(17):6086–114. doi: 10.1038/s44318-026-00867-0 (PMC13534743; doi:10.1038/s44318-026-00867-0)

FigEV2N

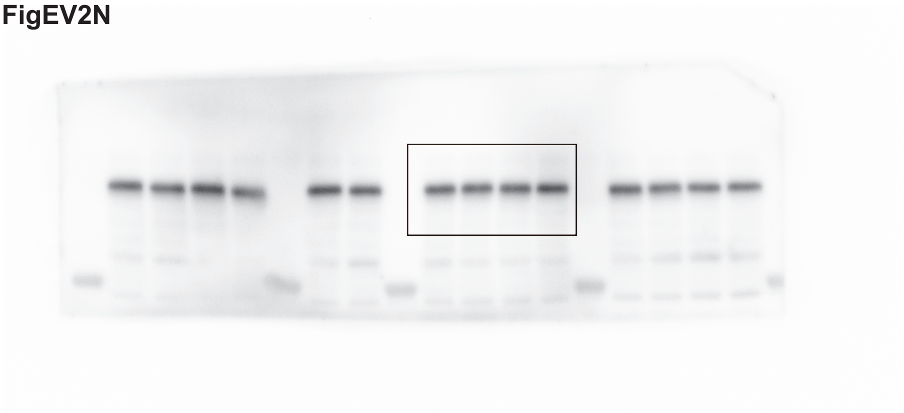

Supplement: Supplementary file 4 [file 44318_2026_867_MOESM11_ESM.zip › Fig EV2-2/N/FigEV2N.pdf]
