# Supplementary material for: Atg23 prevents aberrant fusion of Atg9 vesicles during delivery to autophagosome formation sites
Source: EMBO J. 2026 Jul 20;45(17):6086–114. doi: 10.1038/s44318-026-00867-0 (PMC13534743; doi:10.1038/s44318-026-00867-0)

FigEV5H

Atg9-GFP IP:GFP

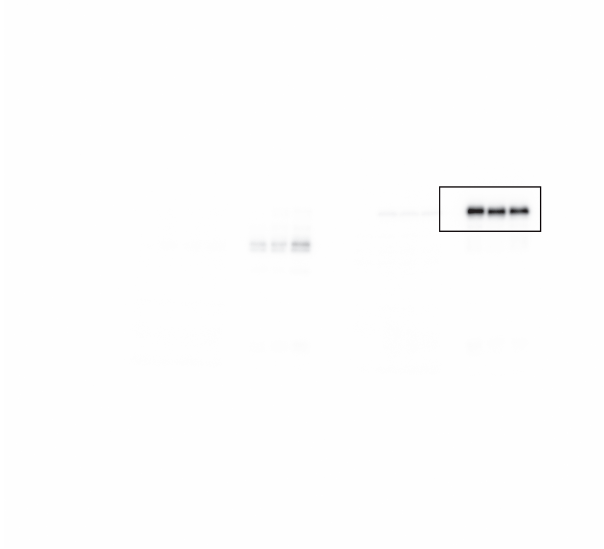

Atg9-GFP Input

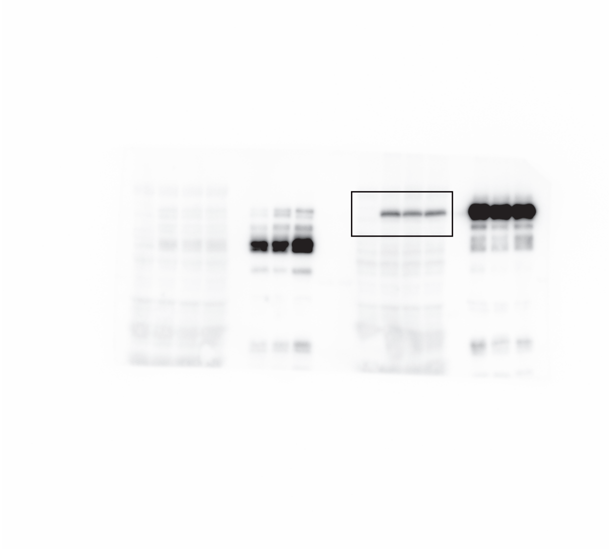

Atg2 IP: GFP

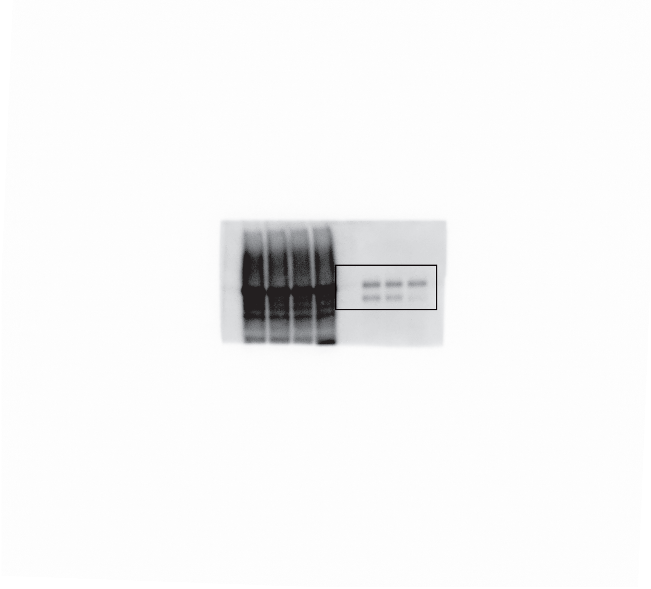

Atg2 Input

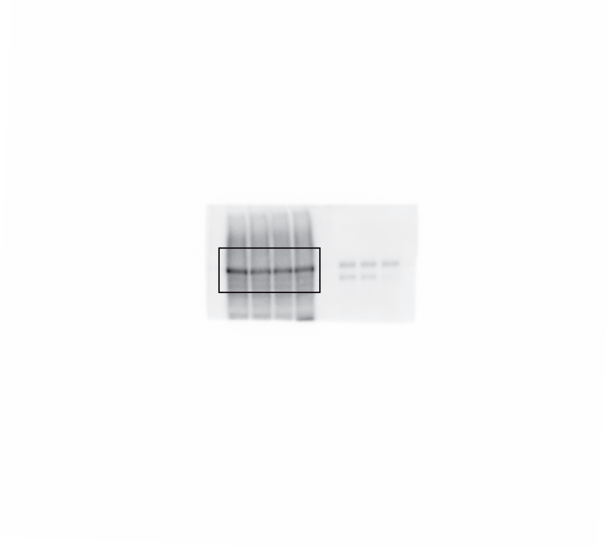

Atg23-FLAG IP: GFP

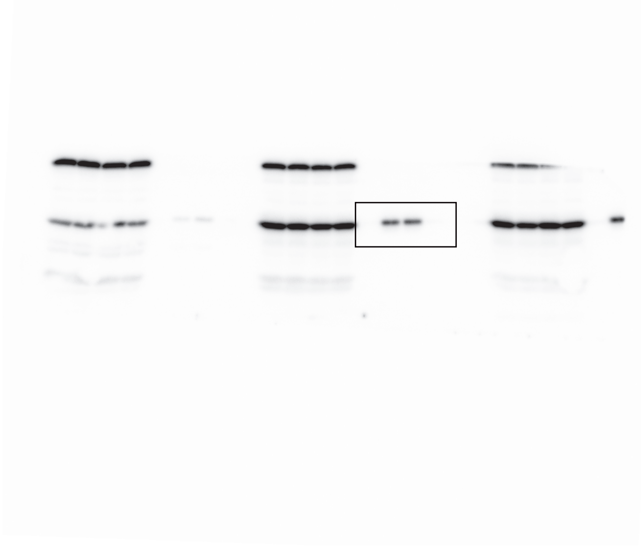

Atg23-FLAG Input

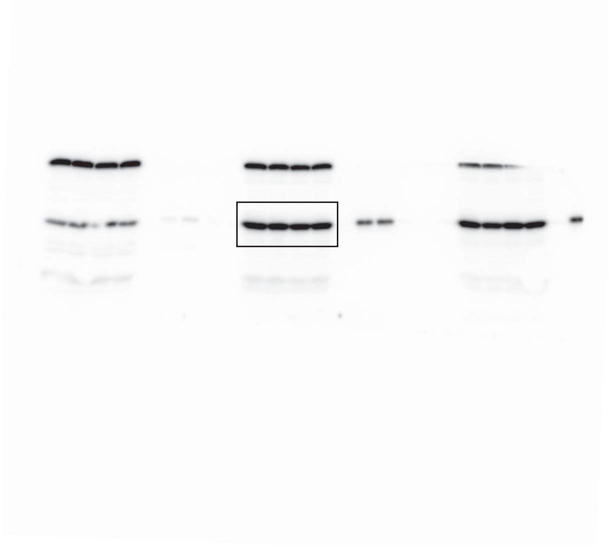

Supplement: Supplementary file 7 [file 44318_2026_867_MOESM14_ESM.zip › Fig EV5/H/FigEV6H.pdf]
