# Supplementary material for: Atg23 prevents aberrant fusion of Atg9 vesicles during delivery to autophagosome formation sites
Source: EMBO J. 2026 Jul 20;45(17):6086–114. doi: 10.1038/s44318-026-00867-0 (PMC13534743; doi:10.1038/s44318-026-00867-0)
Supplement: Supplementary file 7 [file 44318_2026_867_MOESM14_ESM.zip › Fig EV5/K/FigEV5K.pdf]

C

FigEV5K

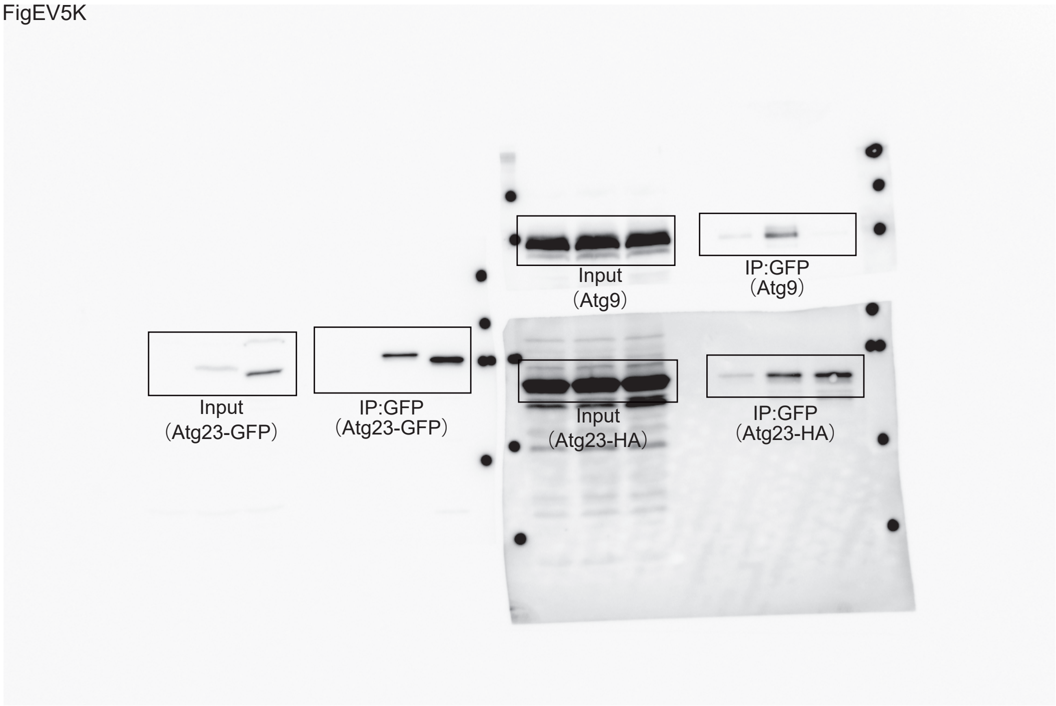
Input
(Atg9)
IP:GFP
(Atg9)
Input
(Atg23-GFP)
IP:GFP
(Atg23-GFP)
Input
(Atg23-HA)
IP:GFP
(Atg23-HA)
