# Supplementary material for: Atg23 prevents aberrant fusion of Atg9 vesicles during delivery to autophagosome formation sites
Source: EMBO J. 2026 Jul 20;45(17):6086–114. doi: 10.1038/s44318-026-00867-0 (PMC13534743; doi:10.1038/s44318-026-00867-0)

Fig2F

Atg1-Gcn4^{CC}-FLAG

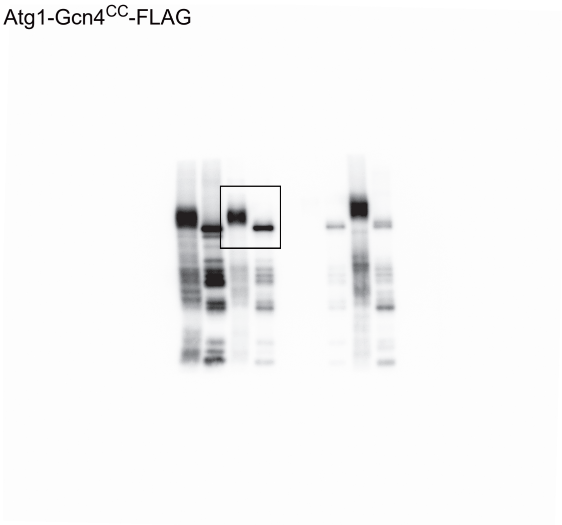

Supplement: Supplementary file 9 [file 44318_2026_867_MOESM3_ESM.zip › Fig2/F/Fig2F.pdf]
