# Supplementary material for: Atg23 prevents aberrant fusion of Atg9 vesicles during delivery to autophagosome formation sites
Source: EMBO J. 2026 Jul 20;45(17):6086–114. doi: 10.1038/s44318-026-00867-0 (PMC13534743; doi:10.1038/s44318-026-00867-0)
Supplement: Supplementary file 9 [file 44318_2026_867_MOESM3_ESM.zip › Fig2/H/Fig2H.pdf]

Fig 2H

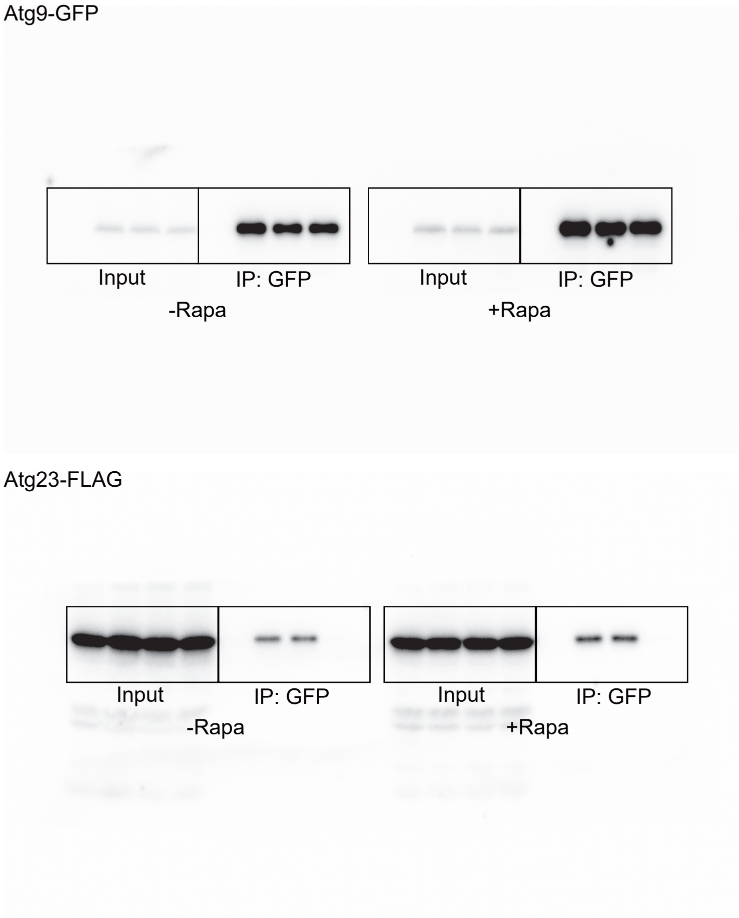
Atg9-GFP
Input
IP: GFP
-Rapa
Input
IP: GFP
+Rapa
Atg23-FLAG
Input
IP: GFP
-Rapa
Input
IP: GFP
+Rapa
